# Assessment and determinants of depression and anxiety on a global sample of sexual and gender diverse people at high risk of HIV: a public health approach

**DOI:** 10.1186/s12889-023-17493-8

**Published:** 2024-01-18

**Authors:** Erik Lamontagne, Vincent Leroy, Anna Yakusik, Warren Parker, Sean Howell, Bruno Ventelou

**Affiliations:** 1 UNAIDS, 20 Ave Appia, 1211 Geneva, Switzerland; 2 Aix-Marseille University, CNRS, EHESS, Centrale Marseille, Aix-Marseille School of Economics, 5-9 Boulevard Maurice Bourdet 13205, Marseille, France; 3 Aix Marseille Univ, Inserm, IRD, SESSTIM, Sciences Economiques & Sociales de La Santé & Traitement de L’Information Médicale, ISSPAM, Marseille, France; 4 Imperial College London, Faculty of Medicine, School of Public Health, London, SW7 2AZ England; 5 University of KwaZulu-Natal, Durban, South Africa; 6 LGBT+ Foundation, San Francisco, CA USA

**Keywords:** LGBT, Gay, Bisexual, Transgender, Queer, Depression, Anxiety, HIV, Homophobia, Stigma and discrimination, PHQ-4

## Abstract

**Background:**

Sexual and gender diverse people face intersecting factors affecting their well-being and livelihood. These include homophobic reactions, stigma or discrimination at the workplace and in healthcare facilities, economic vulnerability, lack of social support, and HIV. This study aimed to examine the association between such factors and symptoms of anxiety and depression among sexual and gender diverse people.

**Methods:**

This study is based on a sample of 108,389 gay, bisexual, queer and questioning men, and transfeminine people from 161 countries collected through a cross-sectional internet survey. We developed a multinomial logistic regression for each group to study the associations of the above factors at different severity scores for anxiety and depression symptoms.

**Results:**

Almost a third (30.3%) of the participants reported experiencing moderate to severe symptoms of anxiety and depression. Higher severity scores were found for transfeminine people (39%), and queer or questioning people (34.8%). Severe symptoms of anxiety and depression were strongly correlated with economic hardship for all groups. Compared to those who are HIV-negative, those living with HIV were more likely to report severe symptoms of anxiety and depression, and the highest score was among those who do not know their HIV status. Transfeminine people were the most exposed group, with more than 80% higher risk for those living with HIV suffering from anxiety and depression. Finally, homophobic reactions were strongly associated with anxiety and depression. The relative risk of severe anxiety and depression was 3.47 times higher for transfeminine people facing transphobic reactions than those with no symptoms. Moreover, anxiety and depression correlate with stigma or discrimination in the workplace and healthcare facilities.

**Conclusions:**

The strong association between the severity of anxiety and depression, and socioeconomic inequality and HIV status highlights the need for concrete actions to meet the United Nations' pledge to end inequalities faced by communities and people affected by HIV. Moreover, the association between stigma or discrimination and anxiety and depression among sexual and gender diverse people is alarming. There is a need for bold structural public health interventions, particularly for transfeminine, queer and questioning people who represent three communities under the radar of national HIV programmes.

**Supplementary Information:**

The online version contains supplementary material available at 10.1186/s12889-023-17493-8.

## Background

Stigma and discrimination based on sex, gender identity and sexual orientation significantly impact all aspects of the lives of sexual and gender diverse people [1], including people who identify as lesbian, gay, bisexual, transgender, queer or questioning, and other sexual, sex- and gender-diverse (LGBT) people and people with intersex traits [2–5]. Under International Human Rights Law, discrimination on the basis of sexual orientation is a human rights violation [6]. The first-ever United Nations resolution on sexual orientation and gender identity was published in November 2011. It requested a report by the Office of the High Commissioner for Human Rights, which stated: "Homophobic and transphobic violence has been recorded in all regions. Such violence may be physical (including murder, beatings, kidnappings, rape, and sexual assault) or psychological (including threats, coercion and arbitrary deprivations of liberty). These attacks constitute a form of gender-based violence driven by a desire to punish those seen as defying gender norms" [7]. In this study, we adopted t [8]he definition of stigma from Link and Phelan, who conceptualise stigma as the co-occurrence of labelling, stereotyping, separation ("us" from "them"), status loss and discrimination [9] in which power is exercised [10]. Discrimination happens at an individual level, where one faces unequal treatment [11, 12] and at the structural level, where societies constrain a person's opportunities, resources and well-being [4]. Homophobic reactions entail emotional, intellectual and behavioural reactions [13] towards sexual and gender diverse people. In this study, we consider homophobia as a particular case of stigma or discrimination based on sexual orientation and gender identity [14]. Discrimination based on sexual orientation intersects with other forms of discrimination towards various groups, including those related to race [15, 16], gender identity [17], age [18], HIV status [19], disability, and socioeconomic status [20, 21].

Evidence suggests that stigma and discrimination impede the health and well-being of sexual and gender diverse people [22]. According to the Joint United Nations Programme on HIV/AIDS (UNAIDS) National Commitments and Policy Instrument (NCPI), 70 countries have discriminatory and punitive laws that criminalise people who engage in same-sex sexual relations, and 20 countries criminalise or prosecute transgender people [23]. Consequently, they are less likely to access health services due to stigma and discrimination [24] and bear a disproportionate burden of adverse physical and mental health outcomes [25]. Evidence suggests that factors, such as stigma, discrimination and violence based on sexual orientation and gender identity, and the criminalisation of same‐sex sexual behaviour, lead to elevated rates of emotional distress and adverse mental health conditions [18], hindering the availability, access and uptake of prevention, testing, treatment and care for HIV, sexually transmitted infections (STIs), and mental health services [19]. The criminalisation of same-sex sexual behaviour in Africa was found to be correlated with lower rates of HIV testing and higher HIV prevalence among gay men and other men who have sex with men [26]. A study of transgender women in Argentina showed that those who had experienced discrimination in healthcare settings were three times more likely to avoid healthcare settings than those who had not [27].

Sexual and gender diverse people face overlapping forms of vulnerabilities related to mental health conditions [28]. They are at higher risk of anxiety, depression, suicidal ideation, substance misuse, and deliberate self-harm than heterosexual people [29]. Mental health conditions further increase the risk of HIV infection, and people living with and affected by HIV have an increased risk of these conditions, which are, in turn, associated with lower retention in HIV care, increased risk behaviours and lower engagement with HIV prevention [6]. Evidence suggests that sexual and gender diverse adolescents and young people -experience higher rates of depression and anxiety and are disproportionately at risk of self-harm and suicide than other adolescents and young people [30]. The prevalence of depression across surveys of people living with HIV in sub-Saharan Africa is estimated at 24%, compared with less than 3% for the general population [31, 32].

Efforts to improve data collection in sexual and gender diverse people are important for monitoring healthcare outcomes and designing healthcare services and programmes. While sex disparities are well documented in public health through nationally notifiable surveillance data, population studies and sentinel surveillance, they stratify by binary sex and only include male or female sex as assigned at birth, which leads to an incomplete understanding of the burden of disease in sexual and gender diverse communities and limits the effectiveness of health and HIV prevention and care programmes [33]. Moreover, the quantitative evidence of the factors driving mental health disparities in sexual and gender diverse people is particularly deficient in low- and middle-income countries.

This study aims to examine the association between the severity of the symptoms of anxiety and depression and factors affecting the well-being and livelihood of sexual and gender diverse people. Using quantitative methods and the data from a global LGBT survey, the study assesses this relationship for specific factors such as homophobic reactions, stigma or discrimination at the workplace and in healthcare facilities, economic vulnerability, lack of social support, and HIV status among people who self-identify as gay men, bisexual men, transfeminine, and queer or questioning men. The study explores two questions: is there a link between the mental health distress measured by the severity of anxiety and depression symptoms in sexual and gender diverse people and the above socioecological factors? If there is a link, does it differ between selected LGBT communities?

## Methods

The analysis presented here draws on the results of the LGBT + Happiness Survey, which collected data from sexual and gender diverse people aged 18 years and older without geographical restrictions. The survey aimed to generate data for sexual and gender diverse people across countries, providing a snapshot of the population's characteristics of interest and outcomes. It captured information on demographics, economic situation, factors influencing happiness, well-being, health, HIV, stigma or discrimination. The survey also considered challenges faced by sexual and gender diverse populations across countries, such as symptoms of depression and anxiety, and experiences of or apprehension about HIV-related discrimination, including in healthcare or the workplace. The survey design was developed collaboratively by UNAIDS, the LGBT + Foundation, the University of Aix-Marseille, the Medical School of the University of Minnesota, and representatives of the LGBT community.

Consenting sexual and gender diverse participants were recruited between May 2019 and January 2020 through social networks, LGBT activists, more than 300 global, regional, and national LGBT community-based organisations, and development partners. Participation was through an anonymous, self-administered, and encrypted Internet-based questionnaire in 32 languages. Questions could be answered using a computer, mobile phone, tablet, or another Internet-linked device. Community-based organisations provided access to the Internet in several African and Caribbean countries where access to the Internet was limited. The survey purposefully did not use cookies, geographic or other identifiers, thus ensuring anonymous and safe participation. This was important for participants who fall within socially marginalised or stigmatised groups in their country and for people who wish to exercise their right to privacy.

Participants were provided with five options for sexual orientation: attracted to men or who identify as gay; attracted to women or who identify as lesbian; attracted to both women and men or who identify as bisexual; those who identify straight or heterosexual; those who do not know or identify as questioning. Regarding gender identity, options were man, woman, transmasculine, transfeminine, or non-binary. Options for sex at birth were male, female, or person with intersex traits.

More than 115,000 participants from more than 200 countries and territories responded to the survey over the May to December 2019 study period. The attrition rate was 2.2% following a review of completed questionnaires—for example, removing participants under the age of 18. The study's final sample was 197 countries with 112,053 sexual and gender diverse participants, including those who identify themselves as lesbian, gay, bisexual, transgender, queer or questioning, other sexuality, sex- and gender-diverse (LGBT) people, and people with intersex traits. A small proportion of the sample (2%) indicated they were living with intersex traits. The detailed research protocol and the questionnaire (English version) are available in the Supplementary material.

### Participants

Sexuality and gender are fluid concepts and include internalised and externalised aspects of gender expression and sexual orientation. The present analysis considers a subsample of sexual and gender diverse people at higher risk of HIV, including people whose biological sex, sexuality, gender identity or gender expression depart from majority norms. This includes those who identify as (i) gay cisgender men; (ii) bisexual cisgender men; (iii) transfeminine people; and (iv) queer/questioning cisgender men. All categories include people with intersex traits. These categories were built from three standardised questions on sex at birth, self-identification of gender identity and self-identification of attraction (without discriminating between sexual, emotional, or physical attraction). The questions were neither mandatory nor prescriptive, i.e., the survey did not provide a definition for each response, acknowledging that participants are best placed to inform how they identify themselves based on their culture, background and self-perception. In addition, the questions were translated and proof-reviewed in more than 32 languages by members from different sexual and gender diverse communities. Appropriate and respectful language was used for each question and answer. The questionnaire was tested and piloted in a dozen countries to ensure the questions, answers, and vocabulary followed the local interpretations and cultural specificities. The questionnaire is available with this link, and translated versions available upon request.

There were 108,329 participants aged 18 and above in this subsample. The sociodemographic characteristics of the participants are presented in Table 1 and Supplementary Material S1 presents their repartition per country and per gender and sexual identity.

**Table 1:** Sociodemographic characteristics of the participants

	**Gay cisgender men or with intersex traits**		**Bisexual cisgender men or with intersex traits**		**Transfeminine people**		**Queer or questioning cisgender men or with intersex traits**	
	**(*N* = 74 730)**		**(*N* = 14 872)**		**(*N* = 4 461)**		**(*N* = 14 326)**	
	**%**	**N**	**%**	**N**	**%**	**N**	**%**	**N**
**Individual characteristics**								
**Symptoms of anxiety and depression**								
**None**	30.8%	22,999	30.6%	4,552	20.1%	898	26.4%	3,787
**Mild**	39.3%	29,423	40.0%	5,949	39.5%	1,762	37.7%	5,394
**Moderate**	16.8%	12,578	17.3%	2,569	21.0%	940	19.7%	2,826
**Severe**	12.4%	9,274	11.5%	1,705	17.9%	798	15.1%	2,160
**Missing**	0.6%	456	0.7%	97	1.4%	63	1.1%	159
**Age groups**	(chi2(2) = 3.8exp03, *p* = 0.000)							
**Young adults (18–24)**	27.37%	20,450	35.59%	5293	37.46%	1,671	35.31%	5,059.00
**Adults (25–34)**	37.68%	28,161	38.01%	5653	38.31%	1,709	39.17%	5,612.00
**Older adults (35 +)**	34.83%	26,029	26.22%	3900	23.74%	1,059	25.18%	3,607.00
**Missing**	0.12%	90	0.17%	26	0.49%	22	0.34%	48
**Education**	(chi2(2) = 458.80, *p* = 0.000)							
**From none to primary education**	2.6%	1926	3.6%	541	7.4%	330	6.4%	909
**Secondary education**	24.2%	18,053	30.3%	4,508	37.7%	1,680	31.1%	4,460
**Post-secondary or University degree**	72.9%	54,504	65.6%	9,754	52.6%	2,345	61.2%	8,773
**Missing**	0.3%	247	0.46%	69	2.4%	106	1.2%	184
**Social and economic inequalities**								
**Subjective socioeconomic status**	(chi2(2) = 266.74, *p* = 0.000)							
**Lower tercile**	24.7%	18,467	27.4%	4,067	36.0%	1,606	31.4%	4,504
**Middle tercile**	38.8%	28,991	38.1%	5,667	35.8%	1,597	34.5%	4,947
**Higher tercile**	35.8%	26,784	33.6	4,990	25.0%	1,116	32.3%	4,623
**Missing**	0.65%	488	1.0%	148	1.8%	142	1.8%	252
**HIV**								
**HIV status**	(chi2(3) = 2.0exp + 03, *p* = 0.000)							
**Negative**	54.6%	40,769	50.3%	7,480	45.3%	2,020	42.3%	6,063
**Positive**	10.9%	8,161	5.7%	853	8.1%	363	8.4%	1,202
**I don't know**	17.0%	12,771	22.8%	3,391	22.4%	1,001	22.0%	3,149
**I don't want to answer**	3.2%	2,400	4.0%	593	5.5%	246	5.7%	814
**Missing**	14.2%	10,629	17.1%	2,555	18.6%	831	21.6%	3,098
**Stigma and discrimination**								
**Homophobic reactions**	(chi2(1) = 6.7843, *p* = 0.009)							
**No**	28.5%	21,303	37.7%	5,605	23.6%	1,046	31.6%	4,509
**Yes**	71.4%	53,367	62.3%	9,247	76.3%	3,373	68.4%	9,757
**Missing**	0.08%	60	0.13%	20	0.94%	42	0.42%	60
**Homophobia at the workplace**	(chi2(1) = 0.7730, *p* = 0.379)							
**No**	82.8%	61,910	82.3%	12,241	65.1%	2,906	76.9%	11,023
**Yes**	13.1%	9,823	11.4%	1,695	24.1%	1,079	18.6%	2,670
**Missing**	4%	2,997	6.3%	936	10.7%	476	4%	633
**S&D in healthcare**	(chi2(1) = 44.7508, *p* = 0.000)							
**No**	94.6%	70,668	93%	13,830	84.6%	3,776	89.2%	12,773
**Yes**	5.4%	4,062	7%	1,042	15.3%	685	10.8%	1,553
**Missing**	-	-	-	-	-	-	-	-
**Perceived social support**								
**Family**	(chi2(4) = 292.79, *p* = 0.000)							
**Strongly disagree**	10.1%	7,605	11.8%	1,755	18.5%	826	12.4%	1,774
**Disagree**	16.1%	12,089	16.4%	2,445	19.0%	847	15.0%	2,158
**Don't know**	18.6%	13,877	25.8%	3,840	21.9%	978	21.7%	3,111
**Agree**	30.3%	22,618	27.7%	4,122	23.8%	1,060	27.4%	3,925
**Strongly agree**	24.6%	18,377	18.0%	2,675	15.7%	702	22.9%	3,287
**Missing**	0.2%	164	0.2%	35	1.0%	48	0.5%	71
**Friend**	(chi2(4) = 157.05, *p* = 0.000)							
**Strongly disagree**	5.4%	4,090	6.9%	1,018	10.1%	451	7.1%	1,012
**Disagree**	10.9%	8,168	11.8%	1,752	14.4%	643	12.7%	1,824
**Don't know**	10.1%	7,529	15.3%	2,268	17.6%	785	16.9%	2,421
**Agree**	37.4%	27,958	37.1%	5,516	33.0%	1,473	34.4%	4,933
**Strongly agree**	35.9%	26,850	28.8%	4,283	23.8%	1,063	28.4%	4,071
**Missing**	0.2%	135	0.2%	35	1.0%	46	0.5%	65

### Description of variables

#### Dependent variable

We explored the predictors of symptoms of anxiety and depression among sexual and gender diverse people. Depression and anxiety are the most common mental disorders and frequently occur together. We used the Patient Health Questionnaire (PHQ-4), a cross-cultural validated [31, 34–41] four-item questionnaire set up to detect symptoms of depression and anxiety manifestations among our subsample's sexual and gender diverse groups [42, 43]. It comprises two items on depression: feeling down, depressed, or hopeless; having little interest or pleasure in doing things, and two items on anxiety: feeling nervous, anxious or on edge; not being able to stop or control worrying. Possible answers follow a 4-item Likert scale ranging from 0 (not at all), 1 (several days), 2 (more than half the days) and 3 (nearly every day). The possible scores ranged from 0 to 12. The index was categorised into none (0–2), mild (3–5), moderate (6–8) and severe (9–12). We tested the robustness of the index using the Cronbach alpha coefficient. The value of the scale reliability coefficient was 0.86, which validates the internal consistency of our index.

#### Independent variables

A set of socioeconomic control variables were first included for each of the sexual and gender identities included in this study, such as age, education (no education/primary school, secondary school, higher education); Economic vulnerability (struggling on present income, neither struggling nor comfortable, living comfortably on present income) and the recency of HIV test as a proxy of possible risk exposure to HIV [44]. Respondents had to choose between the following options: within the last 6 or 12 months, more than 12 months or never.

Thus, two dichotomous variables related to discrimination and stigmatisation of sexual and gender diverse people: the experience of homophobic or transphobic reactions, considered whether the respondent had ever been intimidated/stared at, and/or verbally insulted, and/or physically assaulted during the last 12 months or more, or not. Stigma and discrimination at the workplace considered whether, in the last 12 months, the respondent had their application refused, was harassed or ridiculed at the workplace, was not promoted, was told not to show them being a member of the sexual and gender diverse people, or was denied certain work-related benefits, because of who they are. Stigma and discrimination in healthcare considered whether, in the last 12 months, the respondent experienced verbal or physical abuse, was given a condition (requirement) to change their sexual behaviour or gender identity, or was refused services.

Finally, models included two sources of perceived social support [45]: family and friend support. These variables were assessed with the following questions: "My family accepts me as I am" and "There is someone I can count on if things go wrong". Possible answers followed a 4-item Likert. The variables were dichotomised between those who agree or strongly agree and those who disagree or strongly disagree.

### Statistical model

The intuitive model to address the research question would be the ordered logistic model. However, such model is conditional on the proportional odds assumption (or parallel regression assumption). This key assumption says that the slope of the logistic function is the same for all category cut-offs of the outcome variable [46]. In the present case, the Brant test [47] concluded that the parallel regression assumption was not met, i.e., the slopes of the four stages of the Patient Health Questionnaire are not parallel. In other words, the differences between each stage were not identical. The test results are presented in Supplementary Material S2. Therefore, we developed a multinomial logistic model to adequately reflect the variable characteristics [48]. This technical choice further enabled us to capture the evolution of the independent variables at different severity scores for depression and anxiety symptoms. The base category was no symptoms of anxiety or depression (score 0). The multinomial logistic regression model detects determinants that increase or decrease the relative risk for a participant to suffer from symptoms of anxiety or depression. Considering the possible biases inherent to convenience-based online sampling methods where participants tend to be younger and more educated [49, 50], we included variables age, education and geographical/continental as covariates in the regressions.

We applied a conservative approach to the sample size, using the criteria of total completion, i.e., the regression models only considered participants who informed all variables in the models. We did not impute missing variables. In addition, we successfully tested whether the participants who did not inform their HIV status could create a systematic bias on the dependent variable. See Supplement S3.

We performed the model for the four sexual and gender diverse groups studied. For each model, we assess the validity of the results. The likelihood ratio showed that the independent variables contribute significantly to the predictions of the model. All statistical regressions and tests were performed on Stata 17, and the results were considered significant at *p* < 0.05.

### Ethical approval

The design of the Global LGBT Happiness Survey used in this study was developed collaboratively by UNAIDS, the LGBT Foundation, the University of Aix-Marseille, the Medical School of the University of Minnesota, representatives of the LGBT community and other stakeholders. The Survey was approved by the Research Board of Ethics of Aix-Marseille University (No. 2019–14-03–004) and the Research Ethics Review Committee of the World Health Organization (No. ERC.0003175). All study methods followed the guidelines and principles of the Declaration of Helsinki and the Sex and Gender Equity in Research (SAGER). Participants had to provide their informed consent prior to accessing the survey. The survey protocol fully complied with the European Union's General Data Protection Regulation (GDPR). Participation was voluntary, and no monetary incentive was given to complete the questionnaire. Participants could skip questions or exit at any stage of the questionnaire. Participants who did not provide a numeric value for age or below 18 were excluded from the study. The survey did not collect any identifier or geolocation data although participants could self-report their country of residence.

### Role of the funding source

The funders of this study had no role in study design, data collection, data analysis, or data interpretation. All authors had full access to the data in the study. EL and BV had final responsibility for the decision to submit for publication.

## Results

### Descriptive statistics

Table 1 presents the sociodemographic characteristics of the sample. Among the 108,389 participants, more than two third (68.9%) identified as gay. Bisexual and queer or questioning participants represent about a seventh of the sample, and transfeminine people account for four per cent of the participants.

The proportion of participants suffering from moderate or severe symptoms of anxiety and depression represents almost a third (30.3%) of the whole sample. We noticed that transgender women are the most affected, with 39% reporting moderate to severe symptoms of anxiety and depression. Participants who identified as queer or questioning are the second most affected group, with 34.8% reporting moderate to severe symptoms of anxiety and depression.

Over a third (37.7%) of the participants are adults aged 25 to 34. The highest proportion among young participants (18- to 24-year-old) are those identifying as transfeminine (37.5%). Most participants hold a post-secondary degree, with gay men having the highest proportion holding a post-secondary degree (72.9%) while transfeminine participants have lower education achievement and the highest proportion (7.4%) having only a primary degree or no formal education. In terms of subjective socioeconomic status, gay men are those with the largest proportion (35.8%) of them in the highest tercile, while transfeminine reported the lowest socioeconomic status, with more than a third (36.0%) of them in the lowest socioeconomic tercile and only a quarter of them in the highest tercile.

The self-reported HIV prevalence among all participants is 9.8%, with the highest HIV prevalence being reported among gay men (10.9%) and the lowest among bisexual men (5.7%). These figures should be considered together with the high proportion of participants unaware of their HIV status (18.7%). Gay men have the lowest rate of unknown HIV status (17.0%). In comparison, more than a fifth of bisexual (22.8%), transfeminine (22.4%) and queer or questioning (22.0%) participants are unaware of their HIV serostatus.

Homophobic, stigmatising, or discriminatory reactions are largely prevalent among all sexual and gender diverse groups, with seven in ten participants reporting having faced such reactions. Bisexual men are the community reporting the lowest percentage (62.3%) of such reactions, while more than three-quarters (76.3%) of transfeminine participants reported facing such reactions.

More than one in seven (14.1%) participants declared facing stigma or discrimination at the workplace. The transfeminine persons reported the highest proportion of stigma or discrimination at the workplace, with a quarter of them (24.1%) facing such stigma or discrimination in the last 12 months. Almost one in five (18.6%) queer or questioning participants declared facing stigma or discrimination at the workplace.

Overall, 6.8% of participants indicated they had suffered from stigma or discrimination at health facilities. Transfeminine persons are the community declaring the highest proportion of stigma or discrimination at a healthcare facility, with 15.3% confronted with it, followed by queer or questioning participants (10.8%).

Gay men reported the highest perceived social support, with more than half (54.9%) having their family supporting them and nearly three quarter (73.9%) benefiting from the support of their friends. Transfeminine reported the lowest level of support from their family (39.5%) and friends (56.8%).

### Statistical models on the severity of anxiety and depressive symptoms

**Fig. 1:**
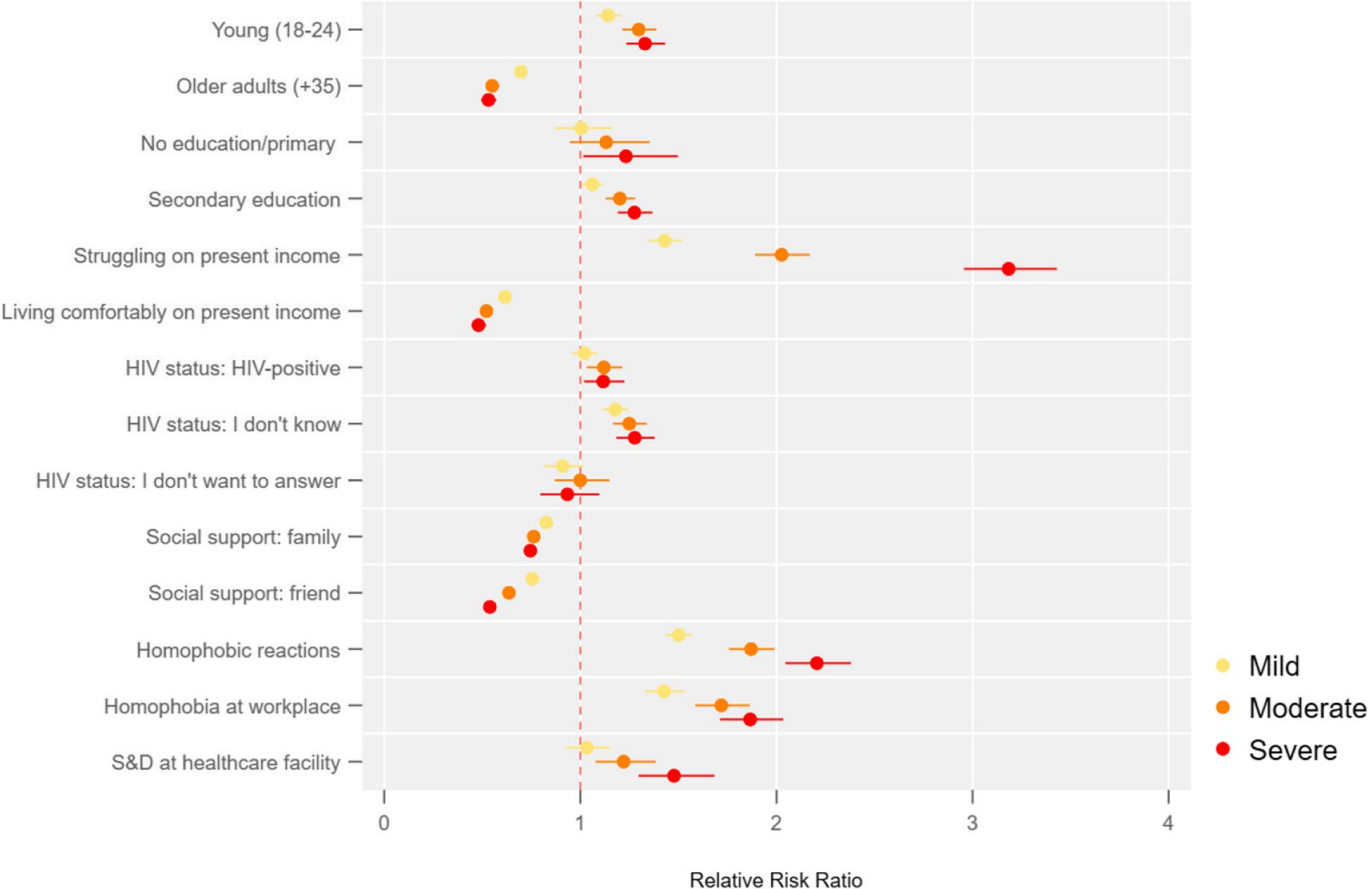
Gay cisgender men or living with intersex traits: association between the severity of symptoms of anxiety and depression, and socioeconomic factors

**Fig. 2:**
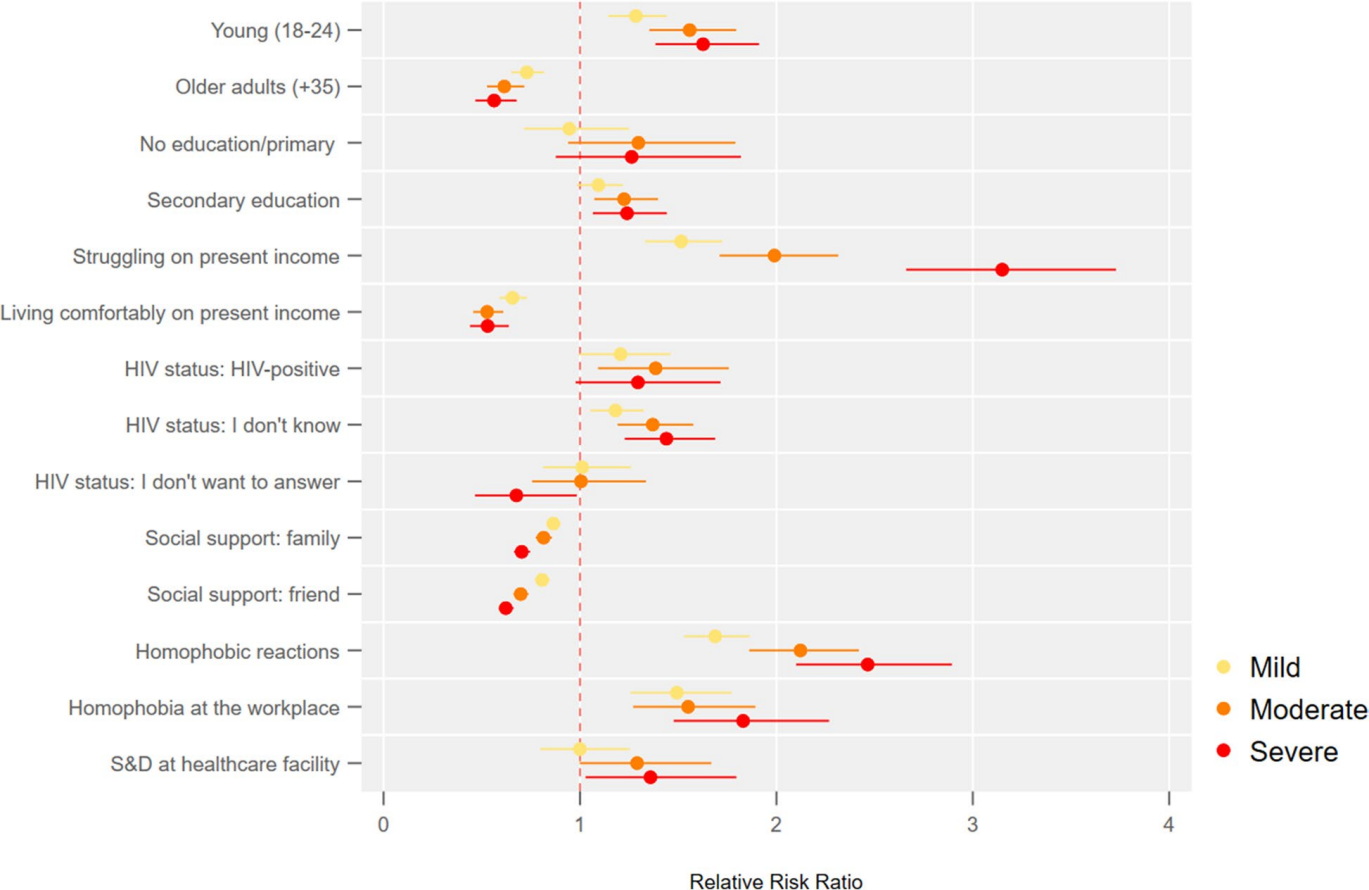
Bisexual cisgender men or living with intersex traits: association between the severity of symptoms of anxiety and depression and socioeconomic factors

**Fig. 3:**
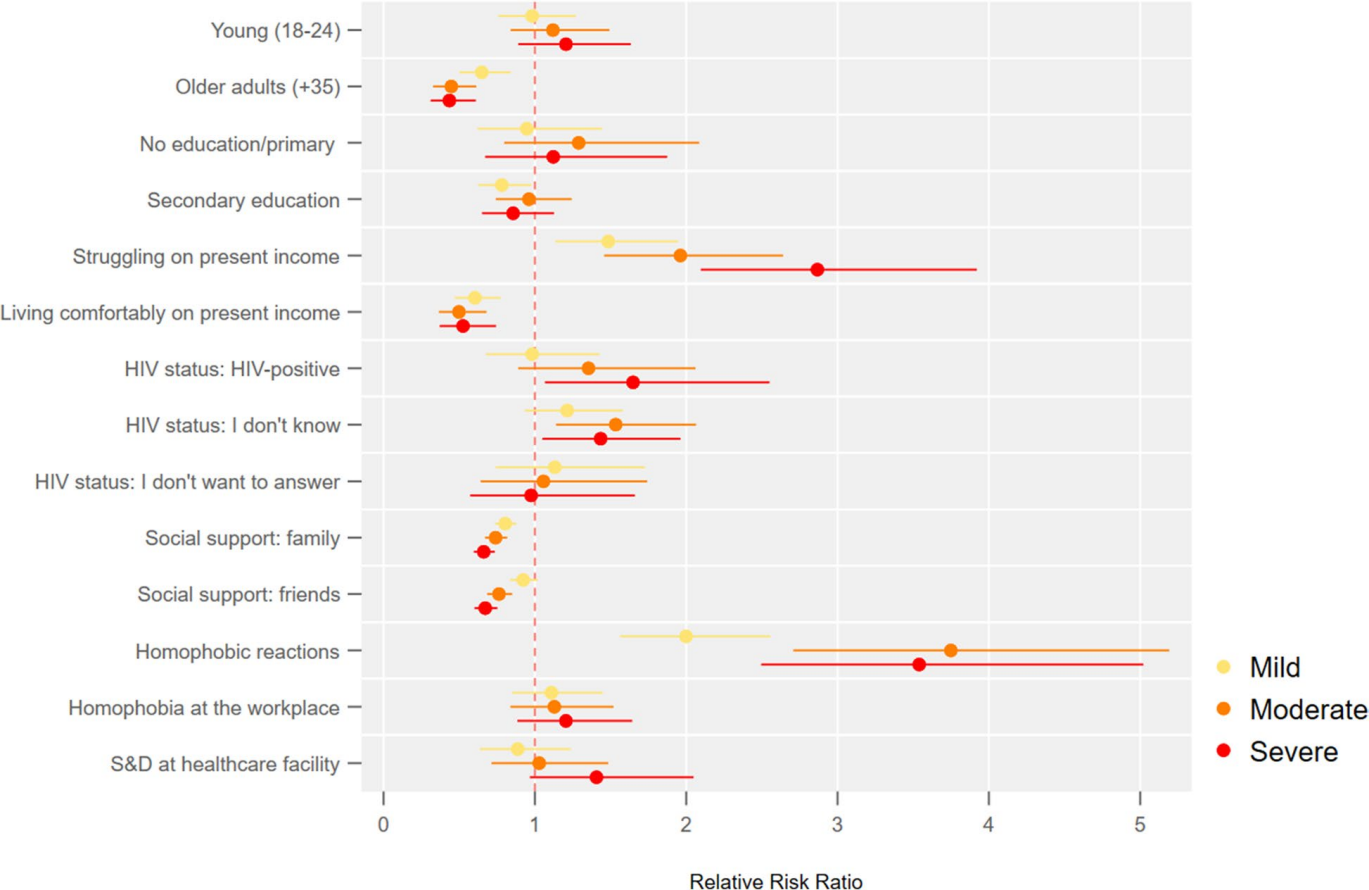
Transfeminine people: association between the severity of symptoms of anxiety and depression and socioeconomic factors

**Fig. 4:**
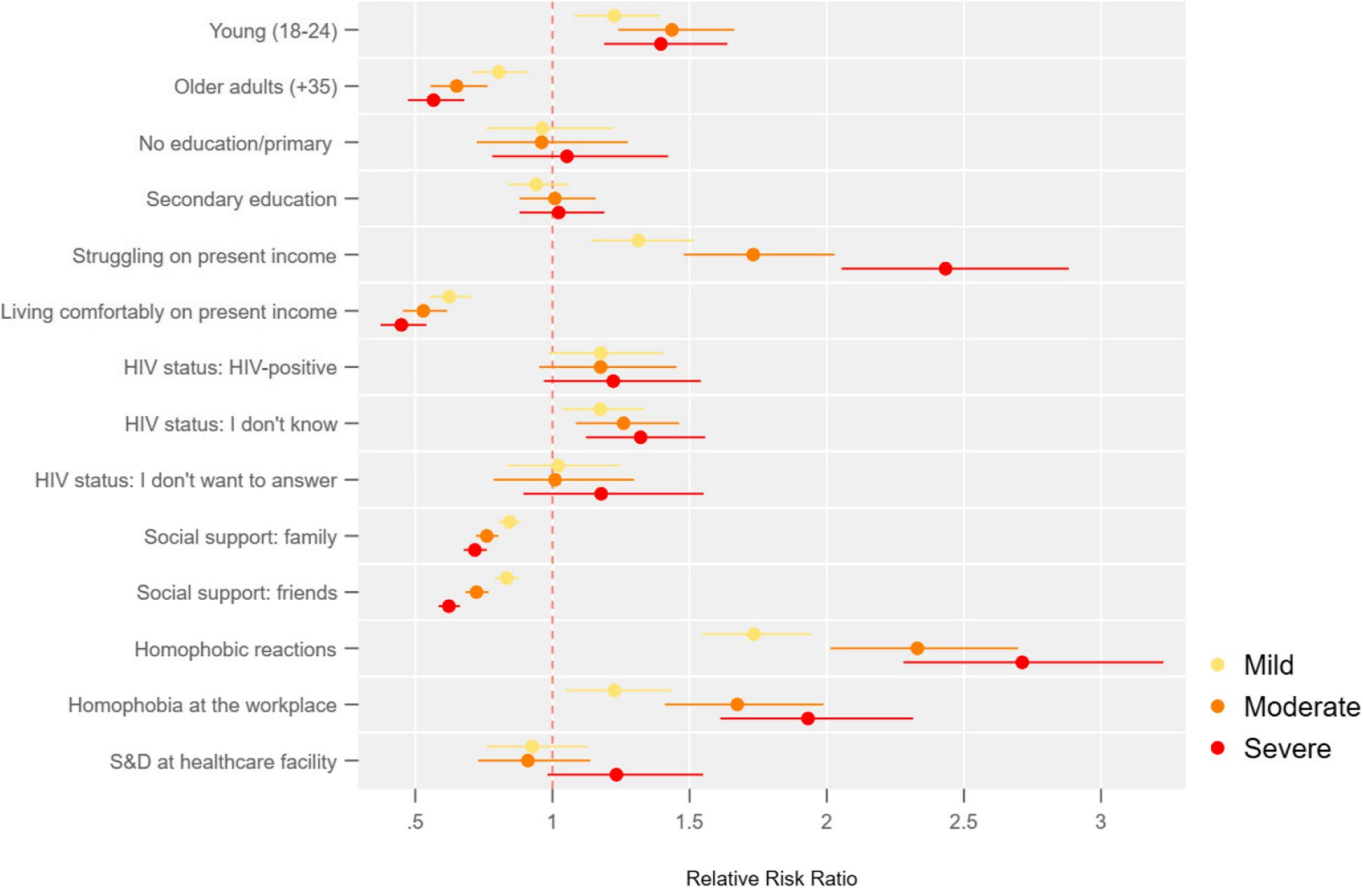
Queer or questioning cisgender men or living with intersex traits: association between the severity of symptoms of anxiety and depression and socioeconomic factors

Figures 1, 2, 3 and 4 below present the results of the multinomial regressions for respectively: (i) gay cisgender men or living with intersex traits; (ii) bisexual cisgender men or living with intersex traits people; (iii) transfeminine people; and (iv) queer or questioning cisgender men or living with intersex traits people. The complete regressions tables can be found in Supplement S4.

For gay men, the risk of having severe symptoms of anxiety and depression is 11%% higher (95%CI 1.02—1.23) for those living with HIV. It increases to 27%% (95%CI 1.18—1.38) for those who do not know their HIV status.

Regarding economic vulnerability, the risk of having severe symptoms of anxiety and depression for gay men struggling with present income is 3.18 times that of those living neither comfortably nor struggling on present income. The risk of having severe symptoms is reduced by 48% (95%CI 0.44—0.52) for those living comfortably on their present income. In other terms, economic empowerment is associated with lower symptoms of anxiety and depression, whereas poverty is associated with increased symptoms of anxiety and depression.

Access to social support, whether through family or friend support, decreases the relative risk of having symptoms of anxiety and depression. For each 1-unit increase in family support (ranked from 1 to 5), the risk of having severe symptoms of anxiety and depression decreases by 25.5%. Similarly, for each 1-unit increase in support from friends, the risk of having severe symptoms decreases by 46.2%.

Facing homophobic reactions is strongly associated with increased symptoms of anxiety and depression among gay men. The risk of having severe symptoms is 2.20 times higher (95%CI 2.05—2.38) for gay men who have faced homophobic reactions. Facing homophobia at the workplace increase the risk of suffering of severe symptoms of anxiety and depression as well, with a RRR of 1.87 (95%CI 1.71—2.03).

Figure 2 shows that most results for bisexual men are similar to those of gay men, with one notable exception. The risk of having severe symptoms of anxiety and depression is 42% (95%CI 1.22—1.68) higher for those unaware of their HIV status.

Figure 3 shows that transfeminine people facing economic hardship are 2.87 times (95%CI 2.10—3.93) higher risk of having severe symptoms of anxiety and depression relative to those with no symptoms. The risk of having severe symptoms of anxiety and depression is 67% higher (95%CI 1.08—2.59) for those living with HIV. Transfeminine people who do not know their HIV status have a 41% higher (95%CI 1.03—1.93) risk of severe symptoms than those with no symptoms.

As the stigmatising and discriminatory reactions faced by transfeminine people increase by one unit, the risk of having severe symptoms of anxiety and depression increases by a factor of 3.47 (95%CI 2.44—4.92) relative to those with no symptoms.

Figure 4 shows that the relative risk of queer or questioning participants having severe symptoms of anxiety and depression is 2.42 times (95%CI 2.04—2.87) that of those living neither comfortably nor struggling on present income.

The risk for queer or questioning people having severe symptoms of anxiety and depression is 31% higher (95%CI 1.11—1.54) for those who do not know their HIV status compared to those who are HIV-negative.

Like transfeminine people, queer or questioning people facing homophobic, stigmatising and discriminatory reactions have a high risk of reporting severe symptoms of anxiety and depression relative to those with no symptoms. Their risk of severe symptoms is multiplied by a factor of 2.72 (95%CI 2.28—3.23) for those who faced homophobic reactions and by 1.91 (95%CI 1.6 – 2.29) for those facing stigma or discrimination at the workplace.

We conducted two post-estimation tests to assess the robustness of our results. First, we have investigated potential multicollinearity issues between the independent variables. The variance inflation factor (VIF) was 1.22, showing that multicollinearity did not threaten our model. We conducted a likelihood ratio test using "mlogtest", a user command [51] for Stata. Results have shown that every independent variable contributes significantly to the model's predictions.

## Discussion

This study explored the role of stigma, discrimination, economic vulnerability, and HIV status in the severity of depression and anxiety symptoms among 108,329 participants, including those living with intersex traits, from 161 countries who identify as transfeminine people, gay, bisexual, and queer or questioning men. Four multinomial logistic regressions enabled us to study the evolution of each predictor on the symptoms of anxiety and depression for each sexual and gender diverse group.

We found that almost a third (30.3%) of the sexual and gender-diverse participants reported suffering from moderate to severe symptoms of anxiety and depression. This proportion rose to almost four in ten (39%) among transfeminine people and more than a third (34.8%) for queer or questioning men. These scores of the severity of anxiety and depression are substantially higher than the ones for the general population, which is around 4% [52].

Overall, we found that for each sexual and gender diverse population group, younger age (except for transfeminine people), low education and income level, seropositivity, homophobic experiences and stigma or discrimination at the workplace and healthcare are associated with a greater risk of suffering from or experiencing severe symptoms of anxiety and depression. In contrast, respondents who have a comfortable income, high levels of family and friends support and who are older tend to have a lesser risk of suffering from severe symptoms of anxiety and depression.

The findings demonstrate the strong association between the severity of depression and anxiety symptoms and economic hardship in all four categories of sexual and gender diverse people. This finding is corroborated by other studies among sexual and gender diverse populations [53, 54] and the general population [55, 56]. This association is particularly acute for gay and bisexual men than for transfeminine people and queer or questioning participants, keeping in mind that participants from the latter two communities are also skewed in the lowest socioeconomic tercile.

The relationship between mental health and HIV risk behaviours has been documented [57], including among sexual and gender-diverse people [58–60]. The study found that the likelihood of reporting severe symptoms of anxiety and depression is significantly higher for those living with HIV [61]. Transfeminine people are the most exposed group, with more than 80% higher likelihood for those living with HIV to suffer from severe symptoms of anxiety and depression. The study further found that gay and bisexual men with severe symptoms of anxiety and depression are statistically more likely to ignore their HIV status. These findings matter as poor mental health is associated with increased HIV risk behaviours such as unprotected anal intercourse, increased number of sex partners, poor HIV continuum of care [62] and negative physical health decisions [63]. It highlights the importance of including mental health support in HIV programmes [61]. This is essential for countries' health systems to reach the sustainable development goal 3 "Ensure healthy lives and promote well-being for all at all ages.

The survey demonstrated that the social support provided by family and friends are two essential components associated with no or low symptoms of anxiety and depression. These findings confirm earlier studies [45, 57, 58, 64, 65]. Lower symptoms of anxiety and depression are associated with support from friends among all sexual and gender categories studied. The relation between the two is particularly strong among transfeminine people. Lower symptoms of anxiety and depression among bisexual, queer or questioning men are also strongly associated with support from family. These findings suggest that public health interventions on mental health with sexual and gender diverse people should increasingly consider the importance of social support from family and friends.

The study examined three forms of stigma and discrimination: *i*) homophobic reactions such as being stared at or intimidated, verbally insulted, or physically assaulted because someone knew or presumed one's sexual orientation or gender identity. *ii*) stigma or discrimination at the workplace; and *iii*) stigma at healthcare facilities. We found that the severity of the symptoms of anxiety and depression was strongly associated with stigma or discrimination based on sexual and gender diversity, corroborating findings from other studies [22, 66, 67]. Considering that homophobia is also associated with a reduction in life expectancy of sexual and gender diverse people [68], these findings call for effective measures and legislation to eliminate homophobia, stigma or discrimination at the workplace and in healthcare services as it contributes to better health outcomes and economic growth [14].

The study has several limitations. The first one relates to representativeness. The participants were recruited through online social networks and community-based organisations at global and country-level. It is based on a non-probabilistic, convenience sampling method that is not meant to represent the sexual and gender diverse population of the countries participating in the study. It is generally acknowledged that convenience sampling methods are subject to selection biases compared to probabilistic samples [69, 70]. The degree and direction in which the selection bias of internet convenience sampling may under or overestimate the relationship between sample characteristics and measured outcomes are difficult to predict and control [3, 71]. We identified and included demographic covariates associated with the potential bias described above to reduce but not eliminate the potential bias [72–74]. The survey intended to include the different sexual and gender diverse people. To our knowledge, it is the first time a global survey has tried to reflect this diversity. Nonetheless, the study had to merge population groups into larger categories for the analysis. This is the case, for example, for the queer or questioning category. Following consultations with sexual and gender diverse representatives, this category included participants who identified themselves as gender and sexual diverse people but not as gay and men who have sex with men, bisexual, or transgender people. Additional studies on specific population groups are required to reflect the extraordinary diversity and the cultural differences of the large spectrum of sexual and gender diverse people. To that effect, data are available to researchers upon request. A second limitation relates to the fact that the survey questionnaire was self-administered, making it impossible to assess the accuracy of the information provided by participants. This includes the self-declared HIV status, amongst others. Nevertheless, the participation was voluntary, anonymous and without any incentive. Therefore, we assume participants had little motive to conceal their preference and HIV serostatus. Third, it is worth noting that some instruments used in the study refer to different periods. For example, the last homophobic reaction is measured in months, and the PHQ-4 for symptoms of anxiety and depression refers to the last two weeks. These validated instruments are built based on the expected frequency of the occurrences of the different events they intend to measure. Finally, the statistical model has an underlying limitation. The current model analysed the compounding effect of independent factors on symptoms of anxiety and depression. The size of the effect of each factor reflects its role, considering the existence of the other factors. This is a contribution to the research question; it does not address how these factors intersect and overlap. Future studies could explore the intersectionality of HIV, homophobia, and economic precarity on the levels of anxiety and depression of LGBT communities.

## Conclusions

This study found that severe anxiety symptoms and depression among sexual and gender diverse people were associated with factors such as low education, economic vulnerability, and socioeconomic status. More importantly, the study also identified a strong relationship between HIV-positive status and the severity of symptoms of anxiety and depression. Moreover, the association between severe symptoms of anxiety and depression is the highest among those who ignore their HIV status. These two findings call for urgent and concrete actions to meet the United Nations' Global Targets to End HIV End Inequality. Both findings are particularly acute among transfeminine, queer and questioning people, three communities that are often under the radar of national HIV programmes. The findings also argue for better, more integrated mental health and HIV services. Finally, the association between stigma and discrimination and the severity of symptoms of anxiety and depression among sexual and gender diverse people is alarming and pushes for bold structural public health interventions incorporating community-level interventions alongside health care provision. Decision-makers and practitioners must pursue and intensify their efforts for inclusive public health policies that promote well-being without discrimination.

### Supplementary Information

**Additional file 1.** Research protocol.

**Additional file 2: Supplement S1.** Final sample per country and per sexual and gender identity. **S2.** Parallel regression assumption. **Supplement S3.** Tests for the exclusion of participants who did not inform their HIV status from the multinomial regressions. **Supplement S4.** Multinomial logistic regressions per sexual and gender-diverse categories.

## Data Availability

The data supporting the findings will be available from the corresponding author upon request following a 6-month embargo from the publication date. Requests will be examined and considered on a case-by-case basis.

## Abbreviations

CI: Confidence interval
COVID-19: Coronavirus infectious disease 2019
HIV: Human Immunodeficiency Virus
LGBT: Lesbian, gay, bisexual and transgender. Short acronym representing all sexual and gender diverse people
PHQ-4: Patient health questionnaire-4
RRR: Relative risk ratio
UNAIDS: Joint United Nations Programme on HIV and AIDS
WHO: The World Health Organization

## References

[1] Müller A (2016). Health for all? Sexual orientation, gender identity, and the implementation of the right to access to health care in South Africa. Health Hum Rights.

[2] Hatzenbuehler ML, Major B, Dovidio JF, Link BG (2017). Structural stigma and health. The Oxford Handbook of Stigma.

[3] Pachankis JE, Hatzenbuehler ML, Hickson F, Weatherburn P, Berg RC, Marcus U (2015). Hidden from health: structural stigma, sexual orientation concealment, and HIV across 38 countries in the European MSM Internet Survey. AIDS (London, England).

[4] Hatzenbuehler ML, Phelan JC, Link BG (2013). Stigma as a fundamental cause of population health inequalities. Am J Public Health.

[5] Augustinavicius JL, Baral SD, Murray SM, Jackman K, Xue Q-L, Sanchez TH (2020). Characterizing cross-culturally relevant metrics of stigma among men who have sex with men across 8 sub-Saharan African countries and the United States. Am J Epidemiol.

[6] Muntarbhorn V, UN Human Rights Council-Independent Expert on Protection against Violence and Discrimination based on Sexual Orientation and Gender Identity, UN Human Rights Council Secretariat. Report of the Independent Expert on Protection against Violence and Discrimination based on Sexual Orientation and Gender Identity: note / by the Secretariat. Report of the Special Procedure of the Human Rights Council. 2017;A/HCR/35/36.

[7] Blondeel K, de Vasconcelos S, García-Moreno C, Stephenson R, Temmerman M, Toskin I. Violence motivated by perception of sexual orientation and gender identity: a systematic review. Bull World Health Organ. 2018;96(1):29–41l. 10.2471/BLT.17.197251 PMC5791869 29403098

[8] Shifrer D, Frederick A (2019). Disability at the intersections. Sociology. Compass.

[9] Link BG, Phelan JC (2001). Conceptualizing stigma. Annu Rev Sociol.

[10] Link BG, Phelan J (2014). Stigma power. Soc Sci Med.

[11] Burgess D, Lee R, Tran A, Van Ryn M (2008). Erratum: Effects of perceived discrimination on mental health and mental health services utilization among gay, lesbian, bisexual and transgender persons (Journal of LGBT Health Research (2007) 3: 4 (1–14)). J LGBT Health Res.

[12] Burgess D, Lee R, Tran A, Van Ryn M (2007). Effects of perceived discrimination on mental health and mental health services utilization among gay, lesbian, bisexual and transgender persons. J LGBT Health Res.

[13] O’Donohue W, Caselles C, Wright RH, Cummings NA (2005). Homophobia: Conceptual, definitional, and value issues. Destructive trends in mental health: the well-intentioned path to harm.

[14] Lamontagne E, d’Elbée M, Ross MW, Carroll A, Plessis Ad, Loures L (2018). A socioecological measurement of homophobia for all countries and its public health impact. Eur J Public Health.

[15] Collins PH (2015). Intersectionality’s definitional dilemmas. Ann Rev Sociol.

[16] Cummings JL, Braboy JP (2008). Race, Gender, and SES Disparities in Self-Assessed Health, 1974–2004. Res Aging.

[17] Sekoni AO, Jolly K, Gale NK (2022). Hidden healthcare populations: using intersectionality to theorise the experiences of LGBT+ people in Nigeria. Africa Global Public Health.

[18] Erwin A, Ma Z, Popovici R, Salas O’Brien EP, Zanotti L, Zeballos Zeballos E (2021). Intersectionality shapes adaptation to social-ecological change. World Dev.

[19] Hagai EB, Annechino R, Young N, Antin T (2020). Intersecting sexual identities, oppressions, and social justice work: Comparing LGBTQ Baby Boomers to Millennials who came of age after the 1980s AIDS epidemic. J Soc Issues.

[20] UNAIDS. HIV and gay men and other men who have sex with men. Human rights fact sheets series. 2021;3(UNAIDS/JC3024E).

[21] UNAIDS. HIV and transgender and other gender-diverse people. Human rights fact sheet series. 2021;4(UNAIDS/JC3024E).

[22] Moagi MM, van der Wath AE, Jiyane PM, Rikhotso RS (2021). Mental health challenges of lesbian, gay, bisexual and transgender people: An integrated literature review. Health SA Gesondheid.

[23] Alencar Albuquerque G, de Lima GC, da Silva QG, Alves MJ, Belém JM, dos Santos Figueiredo FW (2016). Access to health services by lesbian, gay, bisexual, and transgender persons: systematic literature review. BMC Int Health Hum Rights.

[24] Fish JN, Turpin RE, Williams ND, Boekeloo BO (2021). Sexual identity differences in access to and satisfaction with health care: findings from nationally representative data. Am J Epidemiol.

[25] Medina-Martínez J, Saus-Ortega C, Sánchez-Lorente MM, Sosa-Palanca EM, García-Martínez P, Mármol-López MI (2021). Health inequities in lgbt people and nursing interventions to reduce them: A systematic review. Int J Environ Res Public Health.

[26] Stannah J, Dale E, Elmes J, Staunton R, Beyrer C, Mitchell KM, et al. HIV testing and engagement with the HIV treatment cascade among men who have sex with men in Africa: a systematic review and meta-analysis. Lancet HIV. 2019;6(11):e769–87. 10.1016/S2352-3018(19)30239-5 PMC6993044 31601542

[27] Socías ME, Marshall BDL, Arístegui I, Romero M, Cahn P, Kerr T, et al. Factors associated with healthcare avoidance among transgender women in Argentina. Int J Equity Health. 2014;13(1):81. 10.1186/s12939-014-0081-7 PMC4220051 25261275

[28] Williams AJ, Jones C, Arcelus J, Townsend E, Lazaridou A, Michail M. A systematic review and meta-analysis of victimisation and mental health prevalence among LGBTQ+ young people with experiences of self-harm and suicide. PLoS One. 2021;16(1):e0245268. 10.1371/journal.pone.0245268 PMC7822285 33481862

[29] King M, Semlyen J, Tai SS, Killaspy H, Osborn D, Popelyuk D (2008). A systematic review of mental disorder, suicide, and deliberate self harm in lesbian, gay and bisexual people. BMC Psychiatry.

[30] Ream GL (2019). What’s unique about lesbian, gay, bisexual, and transgender (LGBT) youth and young adult suicides? Findings from the National Violent Death Reporting System. J Adolesc Health.

[31] Patel P, Rose CE, Collins PY, Nuche-Berenguer B, Sahasrabuddhe VV, Peprah E (2018). Noncommunicable diseases among HIV-infected persons in low-income and middle-income countries: a systematic review and meta-analysis. AIDS.

[32] Global Burden of Disease Study 2019 (GBD 2019) [Internet]. Institute for Health Metrics and Evaluation (IHME), University of Washington. 2020 [cited 28/08/2022]. Available from: https://vizhub.healthdata.org/gbd-results/.

[33] Shover CL, DeVost MA, Beymer MR, Gorbach PM, Flynn RP, Bolan RK (2018). Using sexual orientation and gender identity to monitor disparities in HIV, sexually transmitted infections, and viral hepatitis. Am J Public Health.

[34] Christodoulaki A, Baralou V, Konstantakopoulos G, Touloumi G (2022). Validation of the Patient Health Questionnaire-4 (PHQ-4) to screen for depression and anxiety in the Greek general population. J Psychosom Res.

[35] Mendoza NB, Frondozo CE, Dizon JIWT, Buenconsejo JU. The factor structure and measurement invariance of the PHQ-4 and the prevalence of depression and anxiety in a Southeast Asian context amid the COVID-19 pandemic. Curr Psychol. 2022:1–10. 10.1007/s12144-022-02833-5 PMC8887801 35250246

[36] Kim H-W, Shin C, Lee S-H, Han C (2021). Standardization of the Korean version of the Patient Health Questionnaire-4 (PHQ-4). Clin Psychopharmacol Neurosci.

[37] Materu J, Kuringe E, Nyato D, Galishi A, Mwanamsangu A, Katebalila M (2020). The psychometric properties of PHQ-4 anxiety and depression screening scale among out of school adolescent girls and young women in Tanzania: a cross-sectional study. BMC Psychiatry.

[38] Siriwardhana C, Abas M, Siribaddana S, Sumathipala A, Stewart R (2015). Dynamics of resilience in forced migration: a 1-year follow-up study of longitudinal associations with mental health in a conflict-affected, ethnic Muslim population. BMJ open.

[39] Kocalevent R-D, Finck C, Jimenez-Leal W, Sautier L, Hinz A (2014). Standardization of the Colombian version of the PHQ-4 in the general population. BMC Psychiatry.

[40] Monahan PO, Shacham E, Reece M, Kroenke K, Ong’or WO, Omollo O (2009). Validity/reliability of PHQ-9 and PHQ-2 depression scales among adults living with HIV/AIDS in western Kenya. J Gen Intern Med.

[41] Hirshfield S, Wolitski RJ, Chiasson MA, Remien RH, Humberstone M, Wong T (2008). Screening for depressive symptoms in an online sample of men who have sex with men. AIDS Care.

[42] Kroenke K, Spitzer RL, Williams JB, Löwe B (2009). An ultra-brief screening scale for anxiety and depression: the PHQ–4. Psychosomatics.

[43] Löwe B, Wahl I, Rose M, Spitzer C, Glaesmer H, Wingenfeld K (2010). A 4-item measure of depression and anxiety: Validation and standardization of the Patient Health Questionnaire-4 (PHQ-4) in the general population. J Affect Disord.

[44] Lamontagne E, Howell S, Yakusik A, Bollinger J, Bouvard L, Ross MW. In: The global internet survey on happiness, health and well-being among sexual and gender minority: design and methods. 2022. (under review).

[45] Zimet GD, Dahlem NW, Zimet SG, Farley GK (1988). The multidimensional scale of perceived social support. J Pers Assess.

[46] McNulty K (2021). Handbook of Regression Modeling in People Analytics: With Examples in R and Python: Chapman and Hall/CRC.

[47] Brant R. Assessing proportionality in the proportional odds model for ordinal logistic regression. Washington, USA. Biometrics. 1990;46(4):1171–8. 2085632

[48] Warner P (2008). Ordinal logistic regression. J Fam Plann Reprod Health Care.

[49] European Centre for Disease Prevention and Control, Hickson F, Schmidt A, Reid D, Weatherburn P, Marcus U, et al. EMIS-2017 – The European men-who-have-sex-with-men Internet survey – Key findings from 50 countries. 2019(TQ-03-19-440-EN-N).

[50] Lamontagne E, Howell S, Yakusik A, Bollinger J, Bouvard L, Ross MW. The global internet survey on happiness, health and well-being among sexual and gender minority: design and methods. 2023 (under review).

[51] Freese J, Long JS, editors. Post-estimation commands for regression models for categorical and count outcomes. North American Stata Users' Group Meetings 2001; 2001: Stata Users Group.

[52] World Health Organization. Depression and other common mental disorders: Global health estimates. Geneva: World Health Organization; 2017. 24 p

[53] Kia H, Robinson M, MacKay J, Ross LE (2021). Poverty in Lesbian, Gay, Bisexual, Transgender, Queer, Two-Spirit, and Other Sexual and Gender Minority (LGBTQ2S+) Communities in Canada: Implications for Social Work Practice. Res Soc Work Pract.

[54] Ross LE, O’Gorman L, MacLeod MA, Bauer GR, MacKay J, Robinson M (2016). Bisexuality, poverty and mental health: A mixed methods analysis. Soc Sci Med.

[55] Ridley M, Rao G, Schilbach F, Patel V (2020). Poverty, depression, and anxiety: Causal evidence and mechanisms. Science.

[56] Richardson T, Maguire N (2020). Poverty, depression, and anxiety: Causal evidence and mechanisms. Science.

[57] Fang L, Chuang D-M, Al-Raes M (2019). Social support, mental health needs, and HIV risk behaviors: a gender-specific, correlation study. BMC Public Health.

[58] Yan Z-H, Lin J, Xiao W-J, Lin K-M, McFarland W, Yan H-J (2019). Identity, stigma, and HIV risk among transgender women: a qualitative study in Jiangsu Province, China. Infect Dis Poverty.

[59] Silvestrini M, Hoff CC, Witkovic YD, Madriles C (2020). 3 HIV/AIDS and Mental Health among Sexual and Gender Minority Populations. The Oxford Handbook of Sexual and Gender Minority Mental Health.

[60] Logie CH, Earnshaw V, Nyblade L, Turan J, Stangl A, Poteat T, et al. A scoping review of the integration of empowerment-based perspectives in quantitative intersectional stigma research. Glob Public Health. 2021:1–16. 10.1080/17441692.2021.1934061 34061710

[61] Tomar A, Spadine MN, Graves-Boswell T, Wigfall LT (2021). COVID-19 among LGBTQ+ individuals living with HIV/AIDS: psycho-social challenges and care options. AIMS public health.

[62] Jeffries WL, Flores SA, Rooks-Peck CR, Gelaude DJ, Belcher L, Ricks PM (2020). Experienced Homophobia and HIV Infection Risk Among U.S. Gay, Bisexual, and Other Men Who Have Sex with Men: A Meta-Analysis. LGBT Health.

[63] Logie CH, Perez-Brumer A, Mothopeng T, Latif M, Ranotsi A, Baral SD (2020). Conceptualizing LGBT Stigma and Associated HIV Vulnerabilities Among LGBT Persons in Lesotho. AIDS Behav.

[64] Smit PJ, Brady M, Carter M, Fernandes R, Lamore L, Meulbroek M (2012). HIV-related stigma within communities of gay men: a literature review. AIDS Care.

[65] Zimet GD, Powell SS, Farley GK, Werkman S, Berkoff KA (1990). Psychometric Characteristics of the Multidimensional Scale of Perceived Social Support. J Pers Assess.

[66] Pachankis JE, Hatzenbuehler ML, Mirandola M, Weatherburn P, Berg RC, Marcus U (2017). The geography of sexual orientation: Structural stigma and sexual attraction, behavior, and identity among men who have sex with men across 38 European countries. Arch Sex Behav.

[67] Drabish K, Theeke LA (2022). Health impact of stigma, discrimination, prejudice, and bias experienced by transgender people: a systematic review of quantitative studies. Issues Ment Health Nurs.

[68] Hatzenbuehler ML, Bellatorre A, Lee Y, Finch BK, Muennig P, Fiscella K (2014). Structural stigma and all-cause mortality in sexual minority populations. Soc Sci Med.

[69] Prah P, Hickson F, Bonell C, McDaid LM, Johnson AM, Wayal S (2016). Men who have sex with men in Great Britain: comparing methods and estimates from probability and convenience sample surveys. Sex Transm Infect.

[70] Drabble LA, Trocki KF, Korcha RA, Klinger JL, Veldhuis CB, Hughes TL (2018). Comparing substance use and mental health outcomes among sexual minority and heterosexual women in probability and non-probability samples. Drug Alcohol Depend.

[71] Pequegnat W, Rosser BRS, Bowen AM, Bull SS, DiClemente RJ, Bockting WO (2007). Conducting Internet-based HIV/STD prevention survey research: considerations in design and evaluation. AIDS Behav.

[72] Steiner PM, Cook TD, Shadish WR, Clark MH (2010). The importance of covariate selection in controlling for selection bias in observational studies. Psychol Methods.

[73] Trutschel D, Palm R, Holle B, Simon M (2017). Methodological approaches in analysing observational data: a practical example on how to address clustering and selection bias. Int J Nurs Stud.

[74] Joyal-Desmarais K, Stojanovic J, Kennedy EB, Enticott JC, Boucher VG, Vo H (2022). How well do covariates perform when adjusting for sampling bias in online COVID-19 research? Insights from multiverse analyses. Eur J Epidemiol.

