# Supplementary material for: Assessment and determinants of depression and anxiety on a global sample of sexual and gender diverse people at high risk of HIV: a public health approach
Source: BMC Public Health. 2024 Jan 18;24:215. doi: 10.1186/s12889-023-17493-8 (PMC10795213; doi:10.1186/s12889-023-17493-8)
Supplement: Supplementary file 1 — Additional file 1. Research protocol. [file 12889_2023_17493_MOESM1_ESM.pdf]

Global LGBTI Internet Survey

Research Proposal

Consolidated final version: 29/04/2019

Content

Abstract ... 3
Research Protocol ... 4
Introduction ... 4
Background ... 4
Rationale for the research ... 4
Methods and Design ... 5
a) The scientific design of the study ... 5
b) Relevance of the research to the health needs of the target population ... 10
c) Sampling strategy, recruitment and voluntary informed consent ... 10
d) Justification of the research design ... 11
e) Gender considerations relevant to the project ... 11
f) The benefits that the research will likely produce ... 11
g) A description of physical, social, psychological and economic risks to both individuals and communities ... 12
h) Vulnerability to harm or to risk of exploitation ... 12
i) Steps that will be taken to minimise the risks to participants, including confidentiality and privacy ... 12
j) Adverse events ... 13
k) Details on how the study will be monitored ... 13
l) Sample size ... 14
m) Compensation of immediate medical or other assistance ... 15
n) Incentives offered to participants ... 15
o) Any information that will be provided to participants following their participation ... 15
p) Limitations of the survey ... 15
Appendix 1: Online Questionnaire ... 17
Appendix 2: Research Board of Ethics ... 33
Appendix 3: Timeline ... 35
Appendix 4: Curriculum vitae ... 37
Appendix 5: Declaration of Confidentiality ... 98
References ... 100

Abstract

Background Worldwide, lesbian, gay, bisexual, transgender and intersex (LGBTI) people contend with high risks for HIV infection, stigma and discrimination, and for exclusion from HIV and other health services. In many low- and middle-income countries, LGBTI people are often hidden, marginalised and difficult to assess as part of general population-based surveys. Thus, assessing their exposures and outcomes and burden of HIV disease is extremely challenging. This presents a dilemma for HIV surveillance as their omission from surveillance systems leaves significant gaps in our understanding of HIV epidemics.

Objective This research project is aimed at conducting an observational, non-interventional, global cross-sectional internet survey in LGBTI participants to examine how various demographic, economic, socioecological, homophobic, psychosocial, attitudinal and behavioural variables potentiate HIV risk behaviour. We likewise aim to examine self-reported HIV prevalence, exposure to HIV related stigma and discrimination, and to study the association between these socioecological parameters and HIV care and treatment coverage in the survey participants.

Methods We imply internet sampling method to conduct the cross-sectional survey recruiting consenting adult LGBTI participants. We select internet sampling for it demonstrated to be particularly useful for hard-to-sample populations, higher number of respondents among hard-to-reach populations, faster recruitment, lower operational cost, greater level of anonymity and security provided to participants. Acknowledging selection bias, we may apply techniques, e.g. prediction modelling, to correct for the bias that results from differential access to, and use of, the internet. To safeguard the data privacy, rights and welfare of the research participants, the access to the online survey will be provided via anonymous weblink. The survey will be opened for three (3) months. Our cross-sectional study is observational by nature and it does not alter the exposure status of the consenting respondents and thus warrants minimal to no risk for the participants. We will examine the outcome and the exposures in the target population and will study their association. Our survey questionnaire provides essential components to fulfil the above objective and was developed using selected standard protocols.

Key words LGBTI, stigma, discrimination, HIV, health, socioecological, cross-sectional, behavioural, Internet, survey

Research Protocol

Introduction

Background

An understanding of HIV burden, risk factors, and coverage of prevention and treatment services is critical for combatting the HIV epidemic. Behavioural surveys assessing these parameters are integral components of a national HIV strategy and surveillance system. Countries that implement repeated behavioural surveys can monitor changes in their populations' risks for HIV, determinants of those risks, and access to prevention and treatment over time.

Many individuals at high risk for HIV are socially marginalized, experience stigma and discrimination, and may not identify themselves as such when accessing services. This makes it difficult to track them in HIV programme registers and hinders efforts to assess the effectiveness of services, their exposures and outcomes. Such populations include LGBTI people, who are at increased risk for HIV infection compared with the general population at large. The underlying reason is that HIV prevalence among their sexual or needle-sharing network is already high. Although members of the general population also engage in frequent vaginal and anal sex without condoms, their chance of having an HIV-infected partner is much lower. Additionally, global prevalence of HIV among MSM is 19 times higher than in the general population, and prevalence among transgenders is 48 times higher.

Rationale for the research

Behavioural surveillance among LGBTI populations is a priority in all epidemic settings. HIV control efforts directed at high-risk populations can have a substantial impact on the epidemic. HIV policies and programmes will be more effective if they are informed by measures of HIV prevalence among those populations, trends in their HIV-related risk behaviours, and the extent to which they access HIV and other health related services. This makes this survey a critical undertaking for monitoring the HIV epidemic and evaluating HIV control efforts.

Policies that criminalize behaviours of high-risk populations or limit their access to HIV and other health related services may further elevate their risk of acquiring and transmitting HIV. The infringement of human rights, verbal and physical violence and denial of health services associated with stigma and discrimination based on sexual orientation and gender identity is a demonstrated public health issue. Stigma is a central driver of morbidity and mortality at a population level[1]. Communities facing enacted sexual stigma are more likely to engage in sexual risk behaviours[2, 3]. They are less likely to adhere to their antiretroviral treatment[4] and have lower HIV testing rates[4-10]. Internalized homonegativity is associated with lower levels of HIV testing and lower levels of condom use[11]. A socio-ecological approach to health-related behaviour[12] allows for studies of the role of stigma related to sexual orientation and gender identity in their broader structural and social dimensions[1, 13, 14]. Most studies of homophobia have focused on the individual level. Being able to identify the level of homophobia at country level is important to guide public health policies as recent evidence shows that reductions in the homophobic climate are associated with improved health outcomes[15]. Data related to structural factors, such as the existence of legislation against LGBTI human rights, are generally available, although unevenly among low- and middle-income countries. But there is a lack of individual and interpersonal data on members of the LGBTI community in low- and middle-income countries.

The ultimate purpose of this research it to facilitate the collection and the analysis of socioecological data in consenting 18+ year-old LGBTI participants that could inform public health action in favour of these communities. This research proposal describes design and methods for conducting this cross-sectional web-based behavioural survey. The project is using a socioecological approach to data analysis, where the behaviour is viewed as being determined by individual, interpersonal and structural factors[12, 16-19].

Methods and Design

a) The scientific design of the study

The survey objectives

This research project is aimed to:

- conduct a cross-sectional observational, non-interventional, internet survey in consenting 18+ year-old LGBTI participants using an anonymous web-based self-administered questionnaire without any geographical restrictions;
- examine correlates of HIV infection to understand how survey variables, such as demographic, economic, socioecological, homophobic, psychosocial, attitudinal and behavioural, potentiate HIV risk behaviour and access to and uptake of HIV care and treatment services;
- provide evidence in favour of more LGBTI-inclusive health and HIV service delivery, advocacy, and policymaking.

Methods

Investigators

The research project involves the Joint United Nations Programmes on HIV/AIDS (UNAIDS), the LGBT Foundation, the University of Aix-Marseille and the Medical School of the University of Minnesota. The investigators are:

- Erik Lamontagne (Senior economist, Strategic Information, UNAIDS): Principal Investigator, in charge of data collection, lead modelling and analysis.
- Sean Howell (President and CEO of The LGBT Foundation): Principal Investigator, owner of the data, in charge of data collection, modelling, analysis.
- Anna Yakusik (Economist, MSc, MBA, Strategic Information, UNAIDS): data collection, modelling, statistical analysis, quality assurance.
- Bruno Ventelou (Professor, Research Director, CNRS, University of Aix-Marseille): modelling, econometric analysis.
- Michael W. Ross (Professor, MD, PhD, MPH, MHPEd, MSt and Chair of Sexual Health Education, University of Minnesota Medical School): modelling, sexuality and psychology analysis.

Scientific peer review

The research proposal has been reviewed and accepted by a scientific peer review group independent from the research project team. The comments from the scientific review group have been incorporated into the final proposal (see appendix 1).

The reviewers are (alphabetical order):

- Abhina Ahder (Associate Director, Sexuality, Gender and Rights, India HIV/AIDS Alliance)
- Martine Audibert (Director of Research, Clermont Auvergne University)
- Lee Badgett (University of Massachusetts Amherst), Asa Radix (Callen-Lorde CHC),
- Stefen Baral (Director, Key Population Program, John Hopkins University)
- Adam Bourne (Director, Australian Research Centre in Sex, Health & Society, La Trobe University)
- Richard Burzynski (Senior Adviser, Civil Society Networking, UNAIDS)
- Alex Garner (Public Policy Office, The Foundation for AIDS Research)
- Peter Ghys (Director, Strategic Information, UNAIDS)
- Asa E. Radix (Senior Director of Research and Education, Callen-Lorde Community Health Center)
- Peter Godfrey-Faussett (Scientific Adviser, UNAIDS)
- Roman Levchenko, Communication Officer, Multimedia, UNAIDS),
- Christoforos Mallouris (Senior Adviser, UNAIDS)
- Keith Sabin (Senior Adviser, Epidemiology, UNAIDS)
- Marc Sangnier (professor, Aix-Marseille School of Economics, Aix-Marseille University
- Bruno Spire (Researcher, National Institute for Medical Research)
- Laurel Sprague (Special adviser, Community Mobilization, UNAIDS)

Target population support for the survey

We engaged with the LGBT Foundation and the members of the target population to seek their input on survey content to ensure that the survey design is consistent with on-the-ground realities, and to further encourage target population participation.

Ethical consideration

The study protocol was assessed and approved by the Research Board of Ethics of the University of Aix-Marseille, and by the WHO Research Ethics Review Committee (ERC). See appendix 2.

Funding

The LGBT Foundation is supporting the research with in-kind contribution of USD 565.000. UNAIDS is providing an additional USD 45,240 from its core budget.

Timing and intervals between the survey rounds

This is the first edition of the global LGBTI survey. Future annual edition of the survey will be determined after the evaluation of the 2019 edition. A tentative timeline for this survey can be found in appendix 3.

One interest of eventual annual editions of the survey is to study the changes and the evolution of key variables over time per country. The study does not track respondents year after year. It will analyse how indicators such as well-being, mental health, HIV self-prevention, homophobia for example are evolving following changes in LGBTI legislation, in government, or in the economy at country level.

This is similar as other surveillance. It typically does not include the same participants in repeat surveys. Ideally, behavioural surveys should be conducted at intervals in order to capture changes in risk behaviours and the HIV epidemic over time. This survey can therefore serve as a baseline for any future undertakings.

Questionnaire development

The questionnaire of the survey contains established instruments for collecting survey data among key populations, such as LGBTI people, using validated questions and scores. These instruments include questions that have been used throughout the world; thus, their continued use will enable researchers to compare survey results across countries. The standardized instruments provided with these guidelines were designed to be self-administered using electronic or web-based data collection methods.

The questionnaire was built on the following four principles:

1. The process must preserve the complete anonymity of respondents. The privacy and security of respondents must be ensured at every step of the survey;
2. Questionnaire should be as short as possible. Only questions that are necessary should be included;
3. Filling out the online questionnaire should expose participants to no more than minimal risk, such as minimal discomfort, minimal anxiety;
4. Questions selected should have their reliability and validity already demonstrated and their collection should enable triangulation with other surveys.

We followed the following **key steps in questionnaire development**:

- decided on methods for questionnaire administration;
- determined investigation topics (questionnaire domains);
- developed and adapted the questionnaire;
- conducted cognitive testing;
- pre-tested the questionnaire;
- pilot tested the survey tool.

The content of the questionnaire is harmonized with standard indicators and protocol checklists used in behavioural surveillance. The questionnaire is organised as modules focused on topics, such as:

- demographics
- time preference, risk aversion
- socioeconomic status, including a question on financing hormones for transgender people
- behaviours increasing vulnerability to HIV infection (e.g. unprotected receptive anal intercourse, buying sex, selling sex, sharing nonsterile injecting equipment, alcohol and drug use)
- stigma and discrimination in the community, at workplace and health facility (sexual orientation, gender identity, HIV)
- internalized homophobia
- violence
- general health status
- mental health, including depression
- social support network, including family
- HIV prevalence, access to HIV and other health services, HIV services uptake:
    - knowledge of serostatus
    - eligible but not on HIV treatment
    - viral load suppression
    - unsuppressed viral load
    - currently in care
    - currently on antiretroviral therapy

  - discussed pre-exposure prophylaxis
  - use of pre-exposure prophylaxis
  - health-care stigma

The consensus questionnaire (see appendix 1) consists of three modules:

- 1st module for LGBTI people, comprising 45 questions;
- 2nd module for MSM and transgender people, comprising 15 questions;
- 3rd module on HIV stigma, comprising 22 questions.

It was our preference to start and finish the survey with relatively comforting questions and statements to reduce any unforeseen discomfort in the participants.

Languages and translations

The survey will be translated in the following additional languages to increase the participation and the representativity of the LGBTI communities: Arabic, Bengali, Chinese, English, Farsi, French, Gujarati, Hindi, Japanese, Marathi, Portuguese, Russian, Spanish, Thai, Turkish, Ukrainian, and Urdu.

Translations are performed from the original English version by native speakers with knowledge in the field of HIV and LGBTI. Translated documents are retranslated to English by a second translator and discrepancies are discussed with the research team and fixed.

Data management

The questionnaire was transferred to an online survey tool, SurveyMonkey ensuring anonymized participation. The initial online transfer was subject to pre-testing. We recruited ten MSM through UNAIDS and community and asked them to work their way through the survey and to express what they were thinking, how the questions struck them, and any ideas that occurred as they proceeded. In response to user comments we adjusted very many small details of the survey, including correcting typographic errors; correcting routing errors; standardising the way individual questions were served; re-ordering modules; reordering flow of questions; rewording/clarification of question stems; reordering of response sets; and expansion of response sets. No questions (items) were cut in this process, although notes were made on what could be considered for the next round of the survey.

We performed a survey consultation to test and provide their feedback on the survey. Issues and question raised were considered and discussed with the consultees. The process resulted few minor changes to the survey. Following the processing of all comments, an item analysis was constructed, and discussion occurred among the core design team to identify the least essential questions while retaining as much topic balance as possible. This version of the survey was subject to an online time-trial.

The LGBT Foundation performed a small-scale test of the online survey with a random set of respondents, in order to estimate the average completion times, possible rejection through disproportionately skipped questions or attrition at a particular point in the completion of the questionnaire. This first online test went adequately and confirmed the interest of respondents in going through the complete survey. The average completion time spent on the survey is 11 minutes.

The online survey is expected to be rolled-out once received the clearance of the WHO ERC, tentatively end of April 2019. For rolling out the survey, we are aiming to establish a network with the following partners for the survey promotion on regional and national online networks:

- The LGBT Foundation Communications Office (https://lgbt.foundation/)
- UNAIDS Press Office (http://www.unaids.org/en/resources/presscentre);

- UNAIDS Office of Community Mobilisation (http://www.unaids.org/en/ourwork/programmebranch/rightsgenderpreventioncommunitymobilizationdepartment/officecommunitymobilization);
- UNAIDS Regional Offices (http://www.unaids.org/en/regionscountries/regions);
- UNAIDS Country Offices (http://www.unaids.org/en/regionscountries/countries).

The survey will be available online for a duration of 12 weeks (3 months). Following which, unidentified data will be provided to the investigators for quality assurance, cleaning and organisation of the database.

Data analysis, use and dissemination

We selected internet sampling for it demonstrated higher number of respondents among hard-to-reach populations, faster recruitment, lower operational cost, greater level of anonymity provided to participants. Acknowledging selection bias, we may apply techniques, e.g. prediction modelling, to correct for the bias that results from differential access to, and use of, the internet. See also section (p) limitations below.

Our cross-sectional study is observational by nature and non-interventional. It does not alter the exposure status of the consenting respondents and thus warrants minimal to no risk for the participants. We will measure the outcome and the exposures in the target population and will study their association to fulfil the study objectives.

Data collected during the survey are anonymised and will be managed in a way that protects the privacy of participants and the confidentiality of the information they provided. We will take measures to prevent the unintended collection of IP addresses.

Data will be owned by the LGBT Foundation. Data will be stored on LGBT Foundation and UNAIDS in their on-premise file storage, protected with always-on real-time firewall and antivirus. Access to the anonymised individual data folder will be password protected and restricted to authorised users, after completion of a Declaration of Confidentiality and Data Privacy. Data are not and will not be monetised. Any data shared outside the survey team – such as with a third party conducting secondary analysis – will be anonymized. When disseminating results, the data will be aggregated at a higher level to ensure that the information does not enable to suspect or reveal the identity of any individual participant or group of participants.

The team will likewise work on the econometric models to answer a first set of two research questions using a socioecological approach:

- Does the homophobic climate increase the vulnerability to HIV infection for LGBTI people?
- Are perceived social and economic inequalities correlated with the incidence of HIV in LGBTI community?

The data can also be used for academic purpose, such as doctoral or post-doctoral articles by the research team. E. Lamontagne might use some of these data for a PhD thesis at Aix-Marseille University.

Country and region-specific analysis

Additional questions may be looked at following this first phase and might include country-specific questions in order to increase the relevance of HIV prevention, care and treatment interventions, zero discrimination interventions and advocacy, or human rights interventions, among others.

Feedbacks of results to communities

Dashboards on key survey indicators will be prepared at country level and posted on the Key Population Atlas web-site and on the LGBT Foundation web-site.

Results and key findings for countries and regions will be prepared in English and made available to LGBTI communities at country, regional and global levels.

Advocacy material will also be prepared by UNAIDS, the LGBTI Foundation and their partners, aiming at promotion of a more inclusive environment for LGBTI, zero discrimination campaign, "Know your status" campaign for HIV prevention, International day against homophobia (IDAHO) and others.

b) Relevance of the research to the health needs of the target population

The number of new HIV infections is decreasing worldwide but incidence is still increasing among the transgender people as well as among the gay and other men having sex with men[20]. Some surveys[14, 21], essentially in high-income countries, suggest that stigma, discrimination, homophobia are associated with poorer mental health and riskier behaviour in terms of vulnerability to HIV infection but data is lacking to study the level of this relationship in low- and middle-income countries and prohibit analysis across countries.

This research is relevant as it aims to fill this information gap in low- and middle-income countries. Findings can be used by partners, LGBTI civil society organisations and UNAIDS country offices for advocacy for more LGBTI-inclusive public health programs.

Furthermore, the survey can provide additional added value thanks to the econometric analysis of socioecological variables together with behavioural variables. To the best of our knowledge, this is the first time such a multi-country data collection on lesbian, gay, bisexual, transgender and intersex people and analysis will be performed. If demonstrated, findings could help addressing socioeconomic inequalities correlated with increased vulnerability to HIV and STI infections.

To our knowledge, this is the first survey provided the scope, focus, and sampling design. Repeated every year this cross-sectional survey could provide useful information on the changing patterns across variables and monitor trends in exposures and outcomes in LGBTI communities. Thus, this study can become a baseline for future research. This study is sought to provide information for public health planning, monitoring, and evaluation at country, regional and global levels.

c) Sampling strategy, recruitment and voluntary informed consent

The target population of the survey are 18+ year-old LGBTI people. The survey can be accessed using a web link that will be provided at LGBTI social networks, e.g. the LGBT Foundation social networks, as well as national and regional LGBTI community networks. The survey will be opened for a duration of 3 months. Recruitment will not involve any direct recruitment and is anonymized. Participation in the survey is not incentivised. We are aware of the limitations associated to these voluntary recruitment channels and we are exploring, with LGBTI civil society organisations and UNAIDS teams at country and regional levels, all additional opportunities that could increase the participation of marginalised or remote communities and individuals.

It was considered that participants who are afraid to share information about risk behaviours or HIV status may drop out of the survey or falsify their responses. To successfully survey LGBTI people, these biases are being minimized by obtaining fully informed voluntary consent at the start of the survey from participants and ensuring their absolute confidentiality.

The informed consent of voluntary participants is summarised at the introduction of the survey and the following information is provided through a link:

- a concise description of the survey and the organization conducting it, the survey objectives, the populations being invited to participate;
- a clear indication that participation is voluntary and that participants have the right of withdrawal from the survey at any time, or skip any sensitive question/s;
- a description of the participant's role in the survey, including duration of participation, the general nature of the questions;
- a description of the possible discomforts of participation;
- a description of the benefits to the community that are expected to come from the survey
- a description of how the findings will be made available to the respondents and the LGBTI community
- measures made to protect the privacy and confidentiality of the participant and any information that the person provides;
- documentation that the protocol was reviewed and approved by Research Ethics Review Committee *(to be completed once WHO ERC approval)*;
- how the results of the survey will be used. This includes explicitly the possible use of the data for scholar purpose, such as doctoral or post-doctoral productions by the research team
- contact information and procedures for contacting the investigators in case of further queries and/or questions.

Only 18+ year-old participants can participate in the survey provided an informed consent. Participants are required to acknowledge their informed consent to enter the survey by clicking on the "OK" button (question 1, mandatory).

d) Justification of the research design

Worldwide, LGBTI people contend with exceptionally high risks for HIV infection, stigma and discrimination, and for exclusion from HIV and other health services. LGBTI people are often hidden and difficult to assess as part of general population-based surveys. Thus, assessing their exposures and outcomes and burden of HIV disease is extremely challenging and necessary. This presents a dilemma for HIV surveillance as their omission from surveillance systems leaves significant gaps in our understanding of HIV epidemics. We aim to fulfil the knowledge and apply web-based community surveying to protect person's privacy and ensure data confidentiality during and after the survey.

e) Gender considerations relevant to the project

The online survey is about people with various sexual orientation and gender identities. We made sure the gender diversity is acknowledged and adequately reflected in the survey.

The survey was provided for review to the members of the LGBTI community with various sexual orientation and gender identities. Their feedback and comments were considered and analysed at various stages of proposal development. The comments were considered and incorporated it the final version of the questionnaire.

f) The benefits that the research will likely produce

To research participants and the LGBTI community

To applied scientific knowledge

The main benefit of the online survey is to inform how the quality of life, stigma -both perceived and enacted-, the discrimination, the social status, the economic vulnerabilities the Lesbian, Gay, Bisexual, Transgender and Intersex People (LGBTI) living in low- and middle-income countries are facing is associated with their vulnerability to HIV infection and to their health status.

The analysis that will follow will support advocacy for more inclusive society and better programme design for LGBTI people in low- and middle-income countries.

g) A description of physical, social, psychological and economic risks to both individuals and communities

The safety and data security of the target population is a top priority in planning and implementation of our survey. We learned from the experience of the previous web-based surveys (e.g. EMIS, etc.), and consulted with the community-based organizations, including the LGBT Foundation. The survey will be conducted anonymously, meaning that personal identifiers will not be recorded on any forms. Unique survey codes will be used in all records and constructed without the use of personal identifying information such as any identifiable numbers.

By design this survey is a cross-sectional survey, where the investigators will measure the outcome and the exposures not altering the exposure status.

The survey instruments were previously administrated in communities and proved minimal to no psychological risk for the respondents. We still acknowledge that some sensitive personal questions may trigger anxiety in some respondents. This can be the case with the following questions and/or statements:

- When was your last HIV test?
- Do you know your HIV status?
- Thinking about your last anal sex partner without any kind of HIV prevention method (that is without condom, PrEP or an undetectable viral load), did you know his HIV status?
- I feel comfortable being sexually attracted to other men.
- I have lost friends by telling them I have HIV.
- The questions of the patient health questionnaire on anxiety and depression (PHQ-4).

However, we anticipate minimal to no risk for the study participants. To monitor any related cases, we created a mailbox to respond quickly and appropriately on any query and/or event related to this survey.

h) Vulnerability to harm or to risk of exploitation

The survey does not imply any person-to-person contact. It does not collect any personal information and records are unidentifiable. The questions, taken all together, do not reveal any information that could make respondents vulnerable to harm or to risk of exploitation. The questionnaire does not use or collect geolocation data, it does not install any tracker cookies on the device of the respondents. We anticipate no harm or risk of exploitation.

i) Steps that will be taken to minimise the risks to participants, including confidentiality and privacy

As described in the section above, the online questionnaire should not entail no more than minimal risk in terms of potential stress and/or anxiety while responding to the questionnaire. The online

questionnaire compiles questions previously used in other surveys and, to the best of our knowledge, do not have any physical risk.

The data privacy and protection of information obtained from participants is important for this survey. To protect the anonymity and data privacy of the participants, the following steps have been taken:

- The study is made available to LGBTI population through a secure, SSL encrypted connection link
- The link does not require any personal identifier, email, phone or person-to-person contact
- Data in transit (ex: while responding online) are encrypted using secure TLS cryptographic protocols
- The collection tool, Survey Monkey, has certified its compliance with the EU-U.S. Privacy Shield Framework (GDPR) and Swiss-U.S. Privacy Shield
- The questionnaire does not collect any personal information. Questions, even taken all together, do not reveal any information that could enable to identify the respondents or his/her geolocation
- The collection tool does not install any targeting or advertising cookies
- IP addresses are instantly decoupled from the questionnaire, encrypted and deleted at the end of the online survey (3 months) by the survey tool
- Data records are unidentifiable

We have, to the best of our knowledge, taken all the measures to prevent any identification of the respondents by the research team as well as by any authority.

We believe we have taken all the necessary steps to ensure data privacy and to protect the confidentiality of information obtained from participants, and to protect them from social or economic discrimination or stigmatization following their participation to the online questionnaire.

Furthermore, as mentioned above, a mailbox representing the participating agencies will be provided to address any query and/or event related to this survey.

j) Adverse events

Building on earlier experiences of non-medical internet surveys (e.g. EMIS, LGBT Survey, LAMIS), the risk of adverse or serious adverse events is considered unlikely and minimal. We are taking considerate steps to safeguard confidentiality for the consenting research participants and data privacy. We anticipate that the risks to the research participants are minimal in relation to potential benefits for the LGBTI community. We will provide adequate provision for oversight and monitoring the safety and data. Also, as mentioned above, we guarantee engagement and will create a mailbox representing the participating agencies to address any query and/or event related to this survey. As such we are aimed to ensure follow up care for participants with any query and/or event related to this survey.

k) Details on how the study will be monitored

The survey is planned to be made available for completion online for 12 weeks. The research team will monitor the situation of the online questionnaire. The research team will monitor the response rate per country and will use country and regional LGBTI websites to post reminders in countries where lower responses than projected are observed.

The mail address for requesting information and right of withdrawal will be opened during all the duration of the survey. This mail address will be administered by a staff from the LGBT Foundation. This staff will not be involved in the survey itself and has completed the Declaration of Confidentiality and Data Privacy. In the case of a request of the right of withdrawal of a participant, the staff in charge will ask if the respondent accepts to reveal, confidentially, her/his IP address. The staff will contact SurveyMonkey to request that any information on the secured SurveyMonkey website, related to this IP address, including the encrypted IP is permanently removed from the survey.

l) Sample size

Various population size estimate of the different LGBTI groups are available of most low- and middle-income countries. The present survey does not pretend to collect a statistically representative sampling of these population groups. The modelling, analyses and findings of this survey will be careful in extrapolating conclusions to the entire population groups of a said country. Rather, we would like to provide statistically robust modelling of the correlates of HIV infection of the participants to the survey. This is in line with current practice in surveillance for key populations as it does not aim at a nationally representative sample of a country's population.

We set the minimum *a priori* sample size of 100 respondents per country studied, corresponding to a 0.10 (.90) probability level for a five predictors multiple regression.

The sample size for multiple regressions will vary for each equation in our model. For example, for an anticipated effect size (f^2) =0.15, a desired statistical power level of 0.90 and a probability level of 0.05 (0.95), a regression with five predictors would require a sample size of 116 respondents.

We study the combination of answers at the level of the individuals. Missing data can be estimated with multiple imputations. We use polychoric matrices for ordinal and categorial data. Multiple choice questions will be treated on a case by case basis. Detailed description of statistical methods will be provided with findings.

Exclusion and Non-qualifiers

The online questionnaire is built in three modules: A socioecological module; a module specific for men having sex with other men; and a module on HIV. Respondents must complete a module and click on 'next' in order to have her/his responses recorded. Any respondent leaving the survey before completing a module is *de facto* excluded from the survey. Respondent choosing to click on the 'exit' button are also excluded from the survey.

To increase the quality of the dataset, the research team will construct discrepancy flags which indicate whether respondents had supplied inconsistent data in different areas.

Non-qualifiers are respondents who did not meet the criteria for inclusion in the study. Non-qualifying cases include:

- Consent not provided
- Not providing a numeric value for age
- Not providing an age of 18 or higher
- Not belonging to any LGBTI groups

Non-respondents

Reflecting the experience of non-respondents is a challenge in such survey where participants are responding on a voluntary and anonymous basis. The proposal acknowledges this limitation. The report will be careful in extrapolating the findings. The report will mention the number of non-

respondents, i.e. people who did not meet the eligibility criteria and people who did not complete the survey.

m) Compensation of immediate medical or other assistance

This research is non-medical by nature and any medical or other assistance is unlikely. Still, we guarantee engagement and will create a mailbox representing the participating agencies to address any query and/or event related to this survey.

n) Incentives offered to participants

We do not use any type of incentives in this research.

o) Any information that will be provided to participants following their participation

The following elements are provided to respondents before they leave the survey:

- A thank you message for their participation and reminder of the anonymity of the survey;
- A weblink to the LGBTI Foundation where additional information on the survey, periodical updates, the general findings and next steps will be provided;
- An advocacy message encouraging participants to get tested for HIV infection.

The survey being anonymous, and the IP addresses being decoupled and encrypted automatically during the time the survey is online (3 months), there is no possibility to provide further direct information to participants after their completion of the questionnaire.

p) Limitations of the survey

This survey will be performed online. This channel is a demonstrated channel enabling higher number of respondents among hard-to-reach populations, faster recruitment, lower operational cost, and greater level of anonymity provided to participants. There are nevertheless limitations that should be acknowledged.

First is the statistical representativity of the survey. There is a selection bias in using social media and social networks. We will apply available techniques to correct for the bias that results from differential access to, and use of, the internet. Like other surveillance for key populations, the survey does not aim of a nationally representative sample of the LGBTI population of a said country.

Second is the access to the internet. Some members of the LGBTI community in low- and middle-income countries may face financial of material constraints to access the internet and thus the survey. UNAIDS Country teams will share the link of the survey with local LGBTI networks, encouraging them to see how they can facilitate an internet access to participants from their community, but it should be acknowledged that members of the LGBTI groups might not be represented.

The third limitation relates to the possible variations between languages. When available, we used validated translations of questions. When translations were not available, we made sure the translations were as close as possible from the original English version. This was achieved by performing translation and retranslation to English as proofreading. Despite of that, the specificities of the different languages make it almost impossible to ensure an exact correspondence. For

example, languages such as Japanese are highly contextual and situational and will thus require more words to describe “being comfortable”.

Finally, due to anonymity, self-reported responses such as country of residence or age cannot be verified by other means. In addition to the informed consent, the question on the age includes the choice “under 18”. Respondents selecting this answer will be considered as non-qualifiers.

Appendix 1: Online Questionnaire

The LGBT Foundation and the United Nations (UNAIDS) partnered with the Universities of Aix-Marseille and Minnesota for this quick survey on happiness, sex and quality of life. You may find more information on the survey, its purpose and first findings on: *(to be completed asap)*

Your responses are completely anonymous.

You can choose to skip any question you would prefer not to answer.

You must be at least 18-year-old to perform this survey. To be in touch, make any information requests, alert us on eventual risks, or exercise your right of withdrawal: research@foundation.lgbt.com

exit

An exit button is always available at the beginning of each module. Respondent can always scroll back to it

☐ OK *(click is required)*

1. *I consent and wish to enter the study anonymously (*required)
   - ☐ yes
   - ☐ no

2. What sex were you assigned at birth?
   - ☐ intersex / ambiguous
   - ☐ female
   - ☐ male

3. How do you define your gender identity? (tick as many as apply)
   - ☐ man
   - ☐ trans man (female to male)
   - ☐ trans woman (male to female)
   - ☐ woman
   - ☐ non-binary gender (Third gender, Hijra)

4. How do you afford your hormones therapy or sex affirmation surgeries? (tick as many as apply)
   - ☐ does not apply to me
   - ☐ most of it is covered by the medical scheme of my country
   - ☐ my private health insurance covers most of it
   - ☐ from my own pocket
   - ☐ I'm working extra hours to afford it
   - ☐ I'm doing sex work to afford it
   - ☐ I regularly sacrifice a meal to pay for it
   - ☐ I can't afford it
   - ☐ I don't need / don't want hormones or sex affirmation surgery for the time being
   - ☐ it is illegal in my country
   - ☐ other

5. How masculine or feminine are you perceived to be by those around you?
   - ☐ very masculine
   - ☐ masculine
   - ☐ neither masculine or feminine
   - ☐ feminine
   - ☐ very feminine

6. What country do you currently live in? **Drop down menu**

7. Which of the following best reflects why you live in this country: (tick as many as apply)

- ☐ was born here, or my parents moved here
- ☐ to study
- ☐ to work
- ☐ to follow a partner
- ☐ to live more openly as gay/bi/lesbian/trans
- ☐ to seek asylum
- ☐ I came as a refugee
- ☐ other

8. How old are you? **Drop down menu**

9. Would you say that you are open (out) as gay, lesbian, bisexual, or trans [answer 1 for not at all open (in) and answer 5 for open (out to all or most people, you know), or answer some place in between]:

- ☐ 1 not at all open (*in*)
- ☐ 2
- ☐ 3
- ☐ 4
- ☐ 5 open (out) to all or most people I know

10. In general, would you say that your health is

- ☐ excellent
- ☐ very good
- ☐ good
- ☐ fair
- ☐ poor

[Section on wellbeing and happiness (note: text in blue does not appear in the questionnaire)]

11. On the whole, I am satisfied with myself.

- ☐ strongly agree
- ☐ agree
- ☐ disagree
- ☐ strongly disagree
- ☐ I don't know

12. How willing are you to take risks, in general?

- ☐ 10 very willing
- ☐ 9
- ☐ 8
- ☐ 7
- ☐ 6
- ☐ 5
- ☐ 4
- ☐ 3
- ☐ 2
- ☐ 1
- ☐ 0 very unwilling

[single-item on time preference]

13. I only act to satisfy immediate concerns, figuring that I will take care of future problems that may occur at a later date

- ☐ extremely characteristic of me
- ☐ somewhat characteristic of me
- ☐ uncertain
- ☐ somewhat uncharacteristic of me
- ☐ extremely uncharacteristic of me

[sub-section: PHQ4]

For the next four questions, think back over the last 2 weeks, how often have you been bothered by the following problems:

14. Feeling down, depressed or hopeless

- ☐ not at all
- ☐ several days
- ☐ more than half the days
- ☐ nearly every day

15. Little interest or pleasure in doing things

- ☐ not at all
- ☐ several days
- ☐ more than half the days
- ☐ nearly every day

16. Feeling nervous, anxious or on edge

- ☐ not at all
- ☐ several days
- ☐ more than half the days
- ☐ nearly every day

17. Not being able to stop or control worrying

- ☐ not at all
- ☐ several days
- ☐ more than half the days
- ☐ nearly every day

18. Imagine a ladder with steps representing happiness in life.
On which step of the ladder do you stand at this time? *(Answer 1 for worst possible life for me, answer 10 for best possible life, or choose a step in between that correspond to you)*

- ☐ 10 best possible life for me
- ☐ 9
- ☐ 8
- ☐ 7
- ☐ 6
- ☐ 5
- ☐ 4
- ☐ 3
- ☐ 2
- ☐ 1
- ☐ 0 worst possible life for me

[Section on social support network]

19. My family accepts me as I am

- ☐ strongly agree
- ☐ agree
- ☐ disagree
- ☐ strongly disagree
- ☐ I don't know

20. There is someone I can count on if things go wrong

- ☐ strongly agree
- ☐ agree
- ☐ disagree
- ☐ strongly disagree
- ☐ I don't know

21. There are people around me who like the same activities I do

- ☐ strongly agree
- ☐ agree
- ☐ disagree
- ☐ strongly disagree
- ☐ I don't know

22. There is someone who counts on me to lend a hand when they need it

- ☐ strongly agree
- ☐ agree
- ☐ disagree
- ☐ strongly disagree
- ☐ I don't know

[Section on body appearance]

23. I consider myself physically attractive

- ☐ strongly agree
- ☐ agree
- ☐ disagree
- ☐ strongly disagree
- ☐ I don't know

24. Most people would consider me good-looking

- ☐ strongly agree
- ☐ agree
- ☐ disagree
- ☐ strongly disagree
- ☐ I don't know

25. I think I am:

- ☐ very underweight
- ☐ somewhat underweight

☐ about normal weight
☐ somewhat overweight
☐ very overweight

[Section on experience of homophobic reactions]

26. Have you ever been **stared at or intimidated** because someone knew or presumed your sexual orientation or your gender identity? If yes, when was the last time?

☐ no, never
☐ yes, within the last 12 months
☐ yes, more than 12 months ago

27. Have you ever heard **verbal insults** directed at you because someone knew or presumed your sexual orientation or your gender identity? If yes, when was the last time?

☐ no, never
☐ yes, within the last 12 months
☐ yes, more than 12 months ago

28. Have you ever been **physically assaulted** because someone knew or presumed your sexual orientation or your gender identity? If yes, when was the last time?

☐ no, never
☐ yes, within the last 12 months
☐ yes, more than 12 months ago

[sub-section on access to healthcare services]

29. When did you last have an HIV test?

☐ within the last 6 months
☐ within the last 12 months
☐ more than 12 months ago
☐ never

30. In the last 12 months, have you avoided going to or delayed going to a health care facility for sexual health or for HIV-related services? (tick as many as apply)

☐ would be too risky for me
☐ I can't pay
☐ I'm embarrassed
☐ last time I felt stigmatised
☐ I'm worried someone may learn about my sexual orientation
☐ it's not convenient (transport, time, distance)
☐ I could not find the times
☐ it was not important to me
☐ I did not avoid or delay my health consultation
☐ other

31. Did you experience any of the following the last time you went to a health facility (tick as many as apply)

☐ I felt welcomed
☐ I had no negative experience

- ☐ verbal abuse (yelling, scolding, name calling, …)
- ☐ physical abuse (pushing, hitting, physically hurt…)
- ☐ was given a condition (requirement) to change my sexual behaviour prior to treatment
- ☐ was given a condition (requirement) to change to change my gender identity prior to treatment
- ☐ refused to help me
- ☐ other

[sub-section on stigma & discrimination at workplace]

32. In the last 12 months, have you experienced any of the following at your current work or when you applied for a job? (tick as many as apply)
    - ☐ I faced no discrimination
    - ☐ my application was refused because I am gay, bi, lesbian, trans
    - ☐ I was harassed or ridiculed at the workplace
    - ☐ I was not promoted because I am gay, bi, lesbian, trans
    - ☐ I was told not to show me being gay, bi, lesbian, trans
    - ☐ I was denied certain work-related benefits because I am gay, bi, lesbian, trans
    - ☐ I was told not to work with clients
    - ☐ I'm not working
    - ☐ I didn't apply because I'm gay, bi, lesbian, trans
    - ☐ other

33. At work, do you earn less than your nearest heterosexual (straight) counterpart who does the same kind of work
    - ☐ yes
    - ☐ no
    - ☐ don't know
    - ☐ does not apply

[Section on quality of sex]

34. Over the past three months, how satisfied are you with the quality of your sexual life?
    - ☐ 10 really satisfied
    - ☐ 9
    - ☐ 8
    - ☐ 7
    - ☐ 6
    - ☐ 5
    - ☐ 4
    - ☐ 3
    - ☐ 2
    - ☐ 1 really not satisfied

35. I would like to have sex
    - ☐ a lot more often
    - ☐ a little more often
    - ☐ I have enough sex as is
    - ☐ a little less often
    - ☐ a lot less often

[Section on sociodemographic data]

36. Your relationship situation

- ☐ single
- ☐ in a relationship with a man
- ☐ in a relation with a transgender person
- ☐ in a relationship with a woman
- ☐ both with a man and a woman
- ☐ I don't know

37. Education (highest degree completed):

- ☐ none
- ☐ primary school
- ☐ secondary /high school
- ☐ university first degree
- ☐ masters/doctorate

38. Current employment status:

- ☐ employed
- ☐ self-employed
- ☐ doing casual or part-time work
- ☐ unemployed
- ☐ student
- ☐ retired
- ☐ other

[Section on economic situation]

39. Which comes closest to your feelings about your income these days?

- ☐ living really comfortably on present income
- ☐ living comfortably on present income
- ☐ neither comfortable nor struggling on present income
- ☐ struggling on present income
- ☐ really struggling on present income

40. Considering all debts or loans of whatever type, were you behind with payments by more than 3 months at any time during the last 12 months?

- ☐ never
- ☐ rarely
- ☐ occasionally
- ☐ most of the time
- ☐ all the time
- ☐ does not apply

41. How often have there been times in your life when you have lived in poverty by the standards of that time?

- ☐ never
- ☐ rarely
- ☐ occasionally
- ☐ most of the time

[Section on social situation]

42. Think of a ladder representing where people stand in **your country**

At the **top** of the ladder are the people who are the **best off**. At the **bottom** are the people who are the **worst off.** Where would you place yourself on this ladder at this moment?

- ☐ 10 among those having most money, most education and

most respected jobs

- ☐ 9
- ☐ 8
- ☐ 7
- ☐ 6
- ☐ 5
- ☐ 4
- ☐ 3
- ☐ 2
- ☐ 1 among those having the least money, least education and least respected jobs or no job

43. Now, think of a ladder representing where people stand in your **local community**
Where would you place yourself on this ladder at this moment?

- ☐ 10 highest social standing in my community
- ☐ 9
- ☐ 8
- ☐ 7
- ☐ 6
- ☐ 5
- ☐ 4
- ☐ 3
- ☐ 2
- ☐ 1 lowest social standing in my community

44. You would say you live in:

- ☐ a major city
- ☐ a medium- or small-size city
- ☐ a village
- ☐ a farm or an isolated house

45. People are different in their sexual attraction to other people. Which best describes your feelings? are you…

- ☐ attracted to other men or gay
- ☐ attracted to other women or lesbian
- ☐ attracted to both men and women or bisexual
- ☐ Straight or heterosexual
- ☐ I don't know

Note: respondents answering 1 (gay), 3 (bisexual) or 5 (I don't know) will be offered to complete module 2

Respondents answering 2 (lesbian) or 4 (straight) will be redirected to question 61 (do you know your HIV status?)

[module 2 for gays, bisexuals, transgenders or men attracted to other men]

exit

An exit button is always available at the beginning of each module. Respondent can always scroll back to it

The following few questions are more specific for gay, bi, transgender and men attracted to other men

☐ OK *(click is required)*

[Section on HIV prevention and exposure to HIV infection]

46. In the past 3 months, with how many **different** sexual partners did you have **anal sex without any kind of HIV prevention method**? That is without condoms, PrEP or an undetectable HIV viral load?

☐ 0
☐ 1, steady
☐ 1, casual
☐ 2
☐ 3 to 10
☐ more than 10
☐ Does not apply to me

47. Thinking about your last **anal sex partner without any kind of HIV prevention method** (that is without condom, PrEP or an undetectable viral load), how did it go?

☐ my partner fucked me (I was receptive)
☐ I fucked him/her (I was insertive)
☐ I both fucked and got fucked (I was both receptive and insertive)
☐ does not apply to me

48. Thinking about your last **anal sex partner without any kind of HIV prevention method** (that is without condom, PrEP or an undetectable viral load), did you know his HIV status? tick as many as apply

☐ I didn't ask
☐ s/he's HIV negative
☐ s/he's HIV positive and I don't know his/her viral load
☐ s/he's HIV positive AND undetectable
☐ I was on PreP
☐ s/he was on PreP
☐ I don't remember
☐ I had more than one partner and I do not know everyone's HIV status
☐ does not apply to me

49. In the last 3 months, how much of the sex you've had with men has been under the influence of alcohol or any other drug?
   - ☐ none of it
   - ☐ almost none of it
   - ☐ less than half
   - ☐ about half
   - ☐ more than half
   - ☐ almost all of it
   - ☐ all of it
   - ☐ does not apply to me

50. In the past 3 months, did you share drug injecting equipment (needles, syringes) with someone else?
   - ☐ yes, once
   - ☐ yes, several times
   - ☐ no, I don't share my equipment
   - ☐ no, I don't inject drugs

51. What do you do if your potential date/sexual partner tells you they are is HIV positive? (tick as many as you like)
   - ☐ I do not see them again
   - ☐ I am not comfortable having sex with them
   - ☐ I am comfortable having sex with them because we use a condom, I am on PrEP and/or he is undetectable
   - ☐ I'm HIV positive too

52. In the last 12 months, how often have **you paid someone** to have sex with you?
   - ☐ never
   - ☐ 1-2
   - ☐ 3-10
   - ☐ 11-50
   - ☐ more than 50 times

53. In the last 12 months, how often have **you been paid** to have sex
   - ☐ never
   - ☐ 1-2
   - ☐ 3-10
   - ☐ 11-50
   - ☐ more than 50 times

54. I feel comfortable in gay bars
   - ☐ 7 strongly agree
   - ☐ 6
   - ☐ 5
   - ☐ 4 undecided
   - ☐ 3
   - ☐ 2
   - ☐ 1 strongly disagree
   - ☐ does not apply

55. Social situations with gay men make me feel uncomfortable
   - ☐ 7 strongly agree

- ☐ 6
- ☐ 5
- ☐ 4 undecided
- ☐ 3
- ☐ 2
- ☐ 1 strongly disagree
- ☐ does not apply

56. I feel comfortable being seen in public with an obviously gay person
    - ☐ 7 strongly agree
    - ☐ 6
    - ☐ 5
    - ☐ 4 undecided
    - ☐ 3
    - ☐ 2
    - ☐ 1 strongly disagree
    - ☐ does not apply

57. I feel comfortable discussing homosexuality in a public situation
    - ☐ 7 strongly agree
    - ☐ 6
    - ☐ 5
    - ☐ 4 undecided
    - ☐ 3
    - ☐ 2
    - ☐ 1 strongly disagree
    - ☐ does not apply

58. I feel comfortable being sexually attracted to other men
    - ☐ 7 strongly agree
    - ☐ 6
    - ☐ 5
    - ☐ 4 undecided
    - ☐ 3
    - ☐ 2
    - ☐ 1 strongly disagree
    - ☐ does not apply

59. Homosexuality is morally acceptable to me
    - ☐ 7 strongly agree
    - ☐ 6
    - ☐ 5
    - ☐ 4 undecided
    - ☐ 3
    - ☐ 2
    - ☐ 1 strongly disagree
    - ☐ does not apply

60. Even if I could change my sexual orientation, I wouldn't
    - ☐ 7 strongly agree

- ☐ 6
- ☐ 5
- ☐ 4 undecided
- ☐ 3
- ☐ 2
- ☐ 1 strongly disagree
- ☐ does not apply

61. Do you know your HIV status?

- ☐ I'm HIV-negative
- ☐ I'm HIV-positive
- ☐ I don't know
- ☐ I don't want to answer

- ☐ PREV *(clicking here brings the respondent to earlier module in case she/he would like to change)*
- ☐ DONE *(brings the respondent to a thank you message)*

Note: participants that have answered "I'm HIV-positive" at question will be offered to participate to module 3 (see next page)

(Thank you message)

Thank you very much for your support.

Sign up to get the results of this study and participate in future studies, your information is completely anonymous, any IP address is decoupled, encrypted and deleted at the end of the study. https://lgbt-token.org/research_signupYou may share the survey via: https://www.research.net/r/LGBTHappinessResearch

Get tested for HIV regularly. For more information: https://hornet.com/about/know-your-status/

(Only participant who answered I'm HIV positive are offered to participate to module 3 below)

exit

An exit button is always available at the beginning of each module. Respondent can always scroll back to it

Module 3: HIV-related stigma and discrimination

If you are HIV-positive, you may face stigma. Let's talk about it

Choose how you feel about each statement. We understand some questions might sound disturbing. Please share your experience. It will help to create a more inclusive environment for everyone

- ☐ OK *(click is required)*

62. I am not as comfortable as I used to be in dating since I learned I have HIV
    - ☐ strongly agree
    - ☐ agree
    - ☐ disagree
    - ☐ strongly disagree
    - ☐ I don't know
63. I disclosed my HIV status on my online profile in the main app I'm using
    - ☐ yes
    - ☐ no
64. If no, is there any reason you don't mention your HIV status? (tick as many as apply)
    - ☐ I revealed it before and couldn't meet anybody
    - ☐ I'm afraid of rejection
    - ☐ I say I am looking for safe sex (like with a condom) and I do not need to tell them my HIV+ status
    - ☐ I don't need to mention it
    - ☐ I'm undetectable
    - ☐ I don't want to mention it
    - ☐ other
    - ☐ does not apply to me (I disclosed my HIV+ status on my profile)
65. I changed the way I have sex since I know I'm HIV positive
    - ☐ yes
    - ☐ no
    - ☐ I don't know
    - ☐ does not apply
66. Since I've known that I'm HIV-positive, I have sex with:
    - ☐ people regardless of their HIV status
    - ☐ only HIV-positive partners
    - ☐ I don't have sex anymore
    - ☐ does not apply

67. Some people avoid touching me once they know I have HIV

- ☐ strongly agree
- ☐ agree
- ☐ disagree
- ☐ strongly disagree
- ☐ I don't know

68. People I care about stopped contacting me after learning I have HIV

- ☐ strongly agree
- ☐ agree
- ☐ disagree
- ☐ strongly disagree
- ☐ I don't know

69. I have lost friends by telling them I have HIV

- ☐ strongly agree
- ☐ agree
- ☐ disagree
- ☐ strongly disagree
- ☐ I don't know

70. Telling someone I have HIV is risky

- ☐ strongly agree
- ☐ agree
- ☐ disagree
- ☐ strongly disagree
- ☐ I don't know

71. I work hard to keep my HIV a secret

- ☐ strongly agree
- ☐ agree
- ☐ disagree
- ☐ strongly disagree
- ☐ I don't know

72. I am very careful whom I tell that I have HIV

- ☐ strongly agree
- ☐ agree
- ☐ disagree
- ☐ strongly disagree
- ☐ I don't know

73. People with HIV are treated like outcasts

- ☐ strongly agree
- ☐ agree
- ☐ disagree
- ☐ strongly disagree
- ☐ I don't know

74. Most people believe a person who has HIV is dirty

- ☐ strongly agree

- ☐ agree
- ☐ disagree
- ☐ strongly disagree
- ☐ I don’t know

75. Most people are uncomfortable around someone with HIV
    - ☐ strongly agree
    - ☐ agree
    - ☐ disagree
    - ☐ strongly disagree
    - ☐ I don’t know

76. I feel guilty because I have HIV
    - ☐ strongly agree
    - ☐ agree
    - ☐ disagree
    - ☐ strongly disagree
    - ☐ I don’t know

77. People’s attitudes about HIV make me feel worse about myself
    - ☐ strongly agree
    - ☐ agree
    - ☐ disagree
    - ☐ strongly disagree
    - ☐ I don’t know

78. I feel I’m not as good a person as others because I have HIV
    - ☐ strongly agree
    - ☐ agree
    - ☐ disagree
    - ☐ strongly disagree
    - ☐ I don’t know

79. In the last 12 months, have you ever been excluded from social gatherings or activities (e.g., weddings, funerals, parties, clubs) because of your HIV status?
    - ☐ yes, once
    - ☐ yes, several times
    - ☐ no
    - ☐ I don’t know
    - ☐ it doesn’t apply to me

80. Are you currently taking HIV (antiretroviral) treatment? (choose one)

- ☐ yes
- ☐ no, medication is not available at the clinic or pharmacy
- ☐ no, medication is not affordable for me
- ☐ no, I am unable to collect medications at the clinic or pharmacy
- ☐ no, I cannot tolerate medication side effects or am worried about taking the pills
- ☐ no, I do not feel treatment is needed
- ☐ no, I am worried someone would find out my HIV status
- ☐ no, I am not ready to deal with my HIV infection
- ☐ no, I am worried the healthcare workers would treat me badly or disclose my HIV status without my consent
- ☐ no, I do not qualify for treatment in my country because my CD4s are too high
- ☐ no, for other reasons

81. In the last 12 months, have you been told you have an undetectable viral load or are virally suppressed ("good viral load")?

- ☐ yes
- ☐ no, I have not had a viral load test in the last 12 months
- ☐ no, I had a viral load test and am waiting for the results
- ☐ no, the virus was detectable/I am not virally suppressed
- ☐ I don't know what viral load or viral suppression are
- ☐ I don't have treatment

82. Living with HIV can open new horizons and positively affects us deep inside. Which of the following have been positively affected by your HIV status (tick as many as apply)

- ☐ My self confidence
- ☐ My self-respect
- ☐ My ability to respect others
- ☐ My ability to cope with stress
- ☐ My ability to better take care of my health
- ☐ My ability to contribute to my community
- ☐ Other
- ☐ None of the above

☐ PREV *(clicking here brings the respondent to earlier module in case she/he would like to change)*

☐ DONE *(brings the respondent to a thank you message)*

(Thank you message) ***see p 12***

Appendix 2: Research Board of Ethics

Research Ethics Committee of Aix-Marseille University

Aix Marseille université

Comité d'éthique de l'université d'Aix-Marseille

Objet : Avis du Comité d'éthique.
N/Réf dossier : 2019-14-03-004
Dossier suivi par :DRV-Audrey Janssens

Pièce(s) jointe(s) : 1 document

Marseille, le samedi 16 mars 2019

Le projet de recherche présenté par l'investigateur principal Dr. Ventelou Bruno, Directeur de recherche au Laboratoire AMSE, UMR AMU CNRS EHESS de l'Université Aix-Marseille et l'investigateur secondaire Lamontagne Erik, PhD et Economiste principal-ONUSIDA , intitulé **«Enquête sur les inégalités sociales et économiques et les facteurs de risque à l'infection au VIH de la communauté LGBT.»** a été soumis pour avis au Comité d'éthique en sa séance du jeudi 14 mars 2019.

Après audition des rapporteurs, le comité a jugé que le projet ne pose pas de problème éthique ou règlementaire.

Le Comité d'éthique de l'Université d'Aix-Marseille émet donc un avis favorable.

Le Président du Comité d'éthique

Pierre-Jean Weiller

Université d'Aix-Marseille - Jardin du Pharo - 58 boulevard Charles Livon - 13284 Marseille cedex 07 - France
Tél. : +33 (0)4 91 39 65 00 - Fax : +33 (0)4 91 31 31 36 - www.univ-amu.fr

WHO Research Ethics Review Committee

From: ERC Secretariat <no-reply-ercsec@who.int>
Sent: 29 April 2019 11:34
To: LAMONTAGNE, Erik <lamontagnee@unaids.org>
Cc: YAKUSIK, Anna <YakusikA@unaids.org>
Subject: ERC.0003175 - Global LGBTI Internet Survey... (France)

Dear Colleague,

We would like to inform you that your project ERC.0003175 has received Final Approval by the Ethics Review Committee. The approval summary can be found in the database (https://extranet.who.int/ercweb/login.php) by clicking on: Review outcome available and then selecting the corresponding protocol at the bottom of the page.

Please note that this approval is valid for one year only. You will receive a reminder for submitting a progress report and continuing review form 2 months before the end of this approval. If you have any questions, please do not hesitate to contact us at ercsec@who.int.

Kind regards,

ERC Secretariat.

Link to the WHO-ERC database: https://extranet.who.int/ercweb/

MSG_COD:f_rev_ro

Appendix 3: Timeline

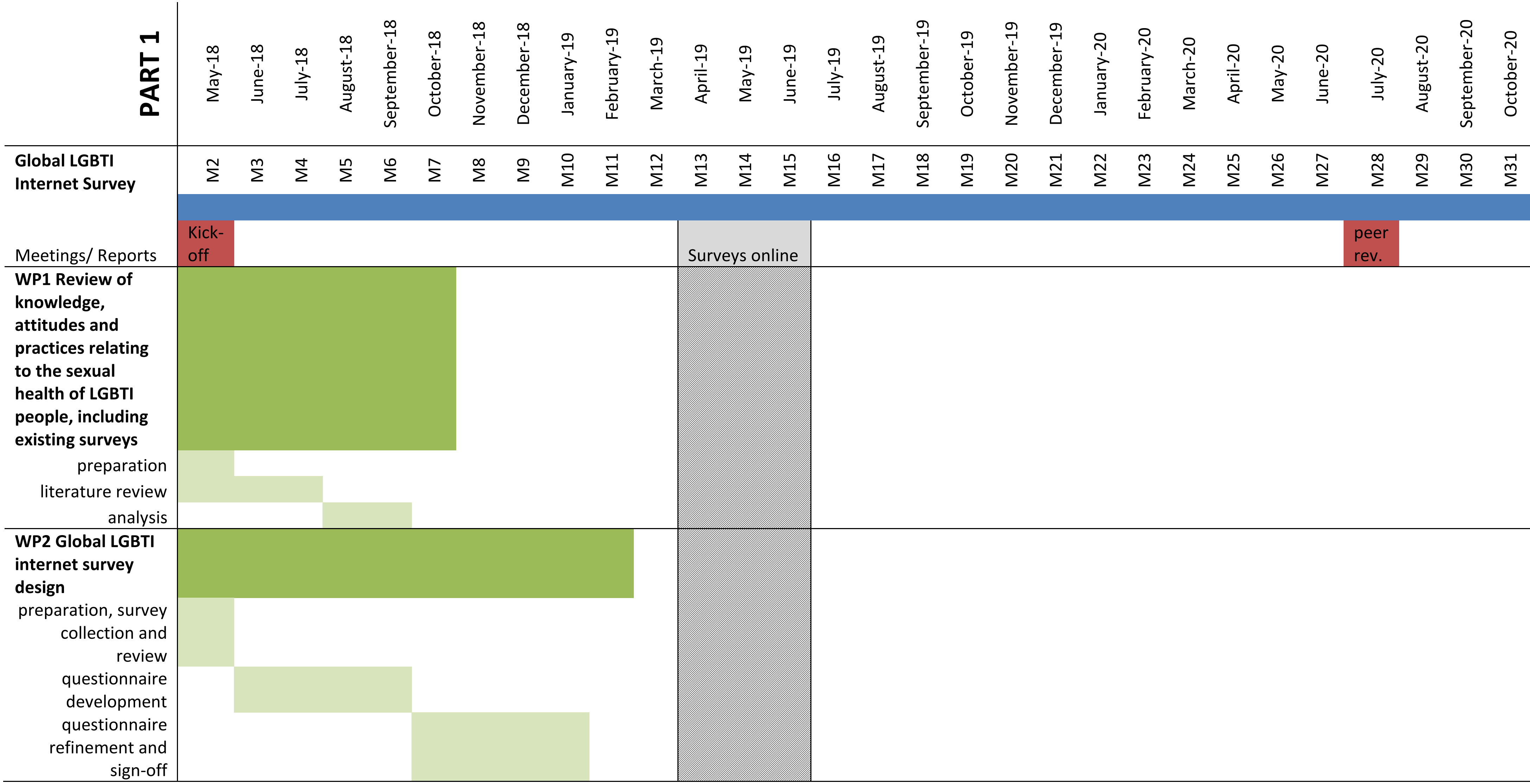

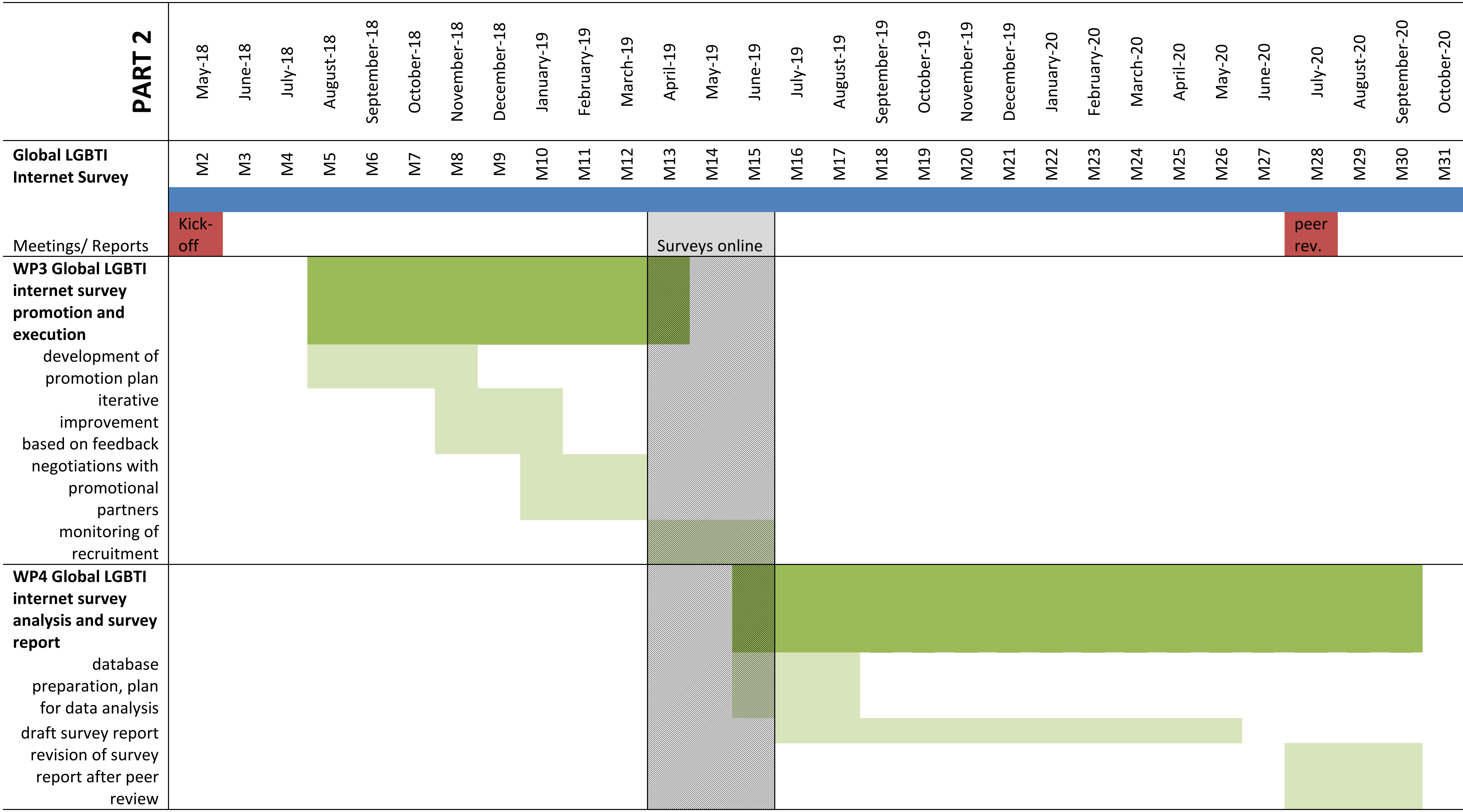
PART 2
May-18
June-18
July-18
August-18
September-18
October-18
November-18
December-18
January-19
February-19
March-19
April-19
May-19
June-19
July-19
August-19
September-19
October-19
November-19
December-19
January-20
February-20
March-20
April-20
May-20
June-20
July-20
August-20
September-20
October-20
Global LGBTI Internet Survey
M2
M3
M4
M5
M6
M7
M8
M9
M10
M11
M12
M13
M14
M15
M16
M17
M18
M19
M20
M21
M22
M23
M24
M25
M26
M27
M28
M29
M30
M31
Meetings/ Reports
Kick-off
Surveys online
peer rev.
WP3 Global LGBTI internet survey promotion and execution
development of promotion plan
iterative improvement based on feedback
negotiations with promotional partners
monitoring of recruitment
WP4 Global LGBTI internet survey analysis and survey report
database preparation, plan for data analysis
draft survey report
revision of survey report after peer review

Appendix 4: Curriculum vitae

LAMONTAGNE, Erik

Personal Details

**Gender: Male**	
**Country and Place of Birth: Québec Canada**	
**Nationality: Canada**	
**Are you currently a UNAIDS staff member?** Yes	

Permanent Address:<br>1700 rue Isabelle-Aubert Appt 603<br>Québec<br>G1M 3X9<br>Canada<br>*Home telephone:* +1 418 4960819	*Professional telephone:*	Present Address (if different from Permanent Address):<br>UNAIDS 20 Via Appia<br>Geneva<br>1211<br>Switzerland<br>Telephone:<br>*Professional telephone:* +41 22 791 45 05<br>*Mobile telephone:* +41 79 201 18 19
Other details:		
E-mail for correspondence: lamontagnee@unaids.org		*Other e-mail address:* erik.lamontagne@laposte.net

Areas of Expertise

Economics, Health<br>Expert<br>over 10 years	Gender Equality<br>Advanced<br>3 - 5 years	Public Health Specialists<br>Advanced<br>6 - 10 years

Language Skills

Mother Tongue 1 : French			Mother Tongue 2 : -
U.N. Proficiency Examination? Yes			
If yes, please indicate the language(s) & year(s) certificate was obtained: English (1994)			
Working Languages of UNAIDS:	*Speaking:*	*Reading:*	*Writing:*

English	Advanced	Advanced	Advanced
French	Mother Tongue	Mother Tongue	Mother Tongue
Spanish	Basic	Basic	Basic
Other languages:			
Arabic	Advanced	Advanced	Intermediate
Portuguese	Basic	Intermediate	Basic

Education

Highest Educational Degree:		Master's Degree (or Equivalent)	
Please indicate other educational details or studies in progress in the text box below (if applicable, publications may be entered in the Additional Information section) then<br>Public Health Economics, Health economics			
Educational details			
Year:	*Name of Education Institution:*	*Degree / Diploma*	*Title of degree/diploma and description of studies:*
2016<br>2019	Aix-Marseille School of Economics<br>Marseille<br>France	Ph. D.	Economics (ongoing) Specialised in public health econometric. developed econometric models on the economics of discrimination
2007<br>2012	United Nations<br>MENA Regional Offfice Cairo<br>Egypt	Certificate	Arabic
2005<br>2010	Ecole de Santé publique, Faculté de Médecine<br>BP 184 54505 Vandoeuvre lès Nancy Cedex<br>France	Diploma	Diplôme universitaire en santé publique option: Developing countries
1989<br>1991	Université Laval<br>Ste-Foy, PQ Canada, G1K 7P4<br>Canada	M.Sc(Econ)	maîtrise en sciences économiques
1986<br>1989	Université Laval<br>Ste-Foy, PQ Canada, G1K 7P4<br>Canada	B. Sc.	Baccalauréat en sciences économiques options: Developing Countries and Econometrics
1983<br>1985	Campus Notre-Dame-de-Foy<br>Cap Rouge, Québec<br>Canada	Diploma	Sciences

International Experience

Are you currently employed by an international organization as explained above? Yes			
Organization:	*Duty Station:*	*From:*	*Grade:*
AI/SIE WHO	Geneva	2010	Fixed-Term Appointment
Have you lived and/or worked outside your home country on a long-term basis (1 year or more)? Yes			
Organization:	Duty Station:	From: - To:	Grade:
MAE	Cameroon	2005 - 2007	Band 2
Commissioneuropéenne	Syria	2002 - 2005	Band 2
PNUD	Mali	1991 - 1994	Band 1
Do you have professional experience outside of your home country (e.g., short-term assignments, management responsibility, extensive business travel)? Yes			
African Region<br>Eastern-Mediterranean Region<br>European Region<br>WHO HQ - Geneva			
Please specify the country and field of work: Middle East as Regional Adviser, Economist and Country Focal Point: Iran Iraq Jordan Lebanon Libya Morocco Palestine Tunisia Abkhazia, Programme officer Azerbaïdjan, Programme Officer Cameroon: Health Economist Guinea Bissau, Programme Officer Mali, Economist (junior) Mali, Programme Officer Mozambique, Programme Officer Niger, Economist Syria: Economist, social sectors Chad, Programme Officer			
A number of jobs in international organizations require that you travel extensively or are prepared to relocate. Are you willing and able to travel during the course of your duties? Yes			
If no, or with reservations, please specify:			

Present and previous employment

12 listed currently			
Exact Title of Position held: Senior Economist			
Name/address of employer:	UNAIDS/WHO<br>20 Ave Appia<br>Geneva	*Name and title of supervisor(s):*	Michel Sidibé, Executive Director, UNAIDS
From:-To: 8/2014 - Present			
Brief description of duties and responsibilities:			
1. provide strategic analysis and recommendations in:<br>- sustainability of Health and HIV financing (domestic and international resources)<br>- modeling for estimating the economic returns of health<br>- linking HIV and health with inequality and poverty<br><br>2. provide innovative strategies to position Health and HIV in the post-2015 development agenda			

3.provide innovative and strategic guidance in Social Health insurance and social protection

4. programme management and team management

Key achievements:

- estimation of the economic returns of ending the AIDS epidemic by 2030
- estimation of the out of pocket expenditures for HIV worldwide
- the economic costs of homophobia and risk of HIV transmission
- to start in 2017: economic study of the demand and supply for testing technologies and antiretroviral therapy in the post-2015 environment

Exact Title of Position held: Regional Investment and Efficiency Adviser / Team leader PPA

Name/address of employer:	UNAIDS regional office for MENA<br>EMRO<br>Cairo, Egypt	*Name and title of supervisor(s):*	Yamina Chakkar, Regional Director

From:-To: 9/2010 - 7/2014

Brief description of duties and responsibilities:

- Generate health economic intelligence for MENA. here, the focus is on:
+ Sustainability of financing for HIV
+ Integration in health systems;
+ Economics of travel restriction
- Develop the Shared Responsibility Agenda in the region
- Perform strategic country support to Iran, Lebanon, Libya, Morocco, Tunisia, Palestine, Syria. Areas of focus includes National Strategy for HIV, lifting bottlenecks hindering implementation of national strategies; Financing of national response and its Sustainability
- Coordinate the interventions of other UN Agencies in the field of HIV at regional level;
- Participation, as resource person, in the new Unified Health Financing model lead by WHO (Geneva)
- Responsible for Workplanning for the country offices; the Regional office; and the Regional Cosponsors intervention on HIV

Key achievements:

- The Macroeconomic Consequences of Renouncing to Universal Access to Antiretroviral Treatment for HIV in Africa: A Micro-Simulation Model- Social health insurance and HIV: a review of experience (peer reviewed, publisehd 2012);
- Long term sustainable financing map for 5 countries in WCA and LAC (UNAIDS papers)
- Policy brief on Social Health Insurance and HIV services, in colloaboration with EIP in HQ (2012)
- Macroeconomic impact of HIV: the need for better modelling (peer reviewed, published 2011)
- The macroeconomic consequences of renouncing to universal access to antiretroviral treatment for HIV in Africa;
- various technical notes on the economics of HIV, the impact of the Economic Crisis; Financing Mechanisms;

TRIPS, etc.
- Various technical notes on UNAIDS functional review (as resource person for MENA)
- Increased efficiency at UNAIDS offices: review and focused workplanning exercises for all MENA countries + RST+ Regional Cosponsors Workplan for HIV

Exact Title of Position held: Economist, Adviser

Name/address of employer:	UNAIDS<br>20 ave Appia<br>1211 Geneva 27, CH	*Name and title of supervisor(s):*	Robert Greener, Paul de Lay

From:-To: 1/2007 - 8/2010

Brief description of duties and responsibilities:

1. Provide senior strategic analysis, technical advice and policy recommendations in the economics of HIV. In particular:
♣ Health financing for HIV;
♣ macroeconomics of HIV at country level;
♣ social protection; and
♣ Sustainable financing (domestic resources)
2. Support the development of improved economic policies in the areas of vulnerability to the impacts of AIDS and the strengthening of social protection.
3. Facilitate and coordinate economic applied research involving multiple international partners and other UN organisations.
4. Keep abreast of economics theory and findings that relate could potentially relate to AIDS. More particularly in the sectors of health economics, microeconomics, public economics, overseas development assistance (ODA) and socio-economics.

Key achievements:

+ Technical analysis and policy advisory support to senior staff on economics and political issues of HIV. This economic knowledge can apply to labour economics and for vulnerable groups like women and youth in particular.
+ Member of the World Bank/UNAIDS Economics Reference Group on HIV
+ Research papers on particular issues with the objective to provide advocacy strategies to UNAIDS senior staff:
♣ Long term sustainable financing for HIV;
♣ Social health insurance and social protection mechanisms
♣ Impact of the economic crisis;
♣ Model for the long term economic impact of the response to HIV;
♣ The Response to HIV as a Global Public Good.
+ Management of research projects and teams involving universities and institutes from Africa and Latin America.:

♣ Paper on the interaction of MDG6 with the other MDGs: getting AIDS out of isolation;
♣ Member of the UN system joint initiative on the Social protection floor;

Exact Title of Position held: Economist, Technical Adviser to the MoH

Name/address of employer:	Ministère des Affaires étrangères<br>23, rue de la Pérouse<br>75775 Paris cedex 16	*Name and title of supervisor(s):*	Jean-Pierre Lamarque

From:-To: 1/2005 - 1/2007

Brief description of duties and responsibilities:

1. Senior Technical Adviser to the Ministry of Public Health
2. Provide senior technical advice and policy recommendations for heath financing and optimisation of resources available
3. Provide capacity building in terms of econmics, planning and budgeting for civil servant from the Ministry of Health
4. Responsible for the preparation of the first debt cancellation contract (C2D) of the French government in the health sector, representing Euro 90 million.
5. Technical adviser to the Minister for:

♣ Sector wide approach (SWAp) in health;
♣ Aid coordination and implementation of Paris' Declaration on aid effectiveness;
♣ Public expenditures and public health budget.

Key achievements:

+ 5 programmes representing Euro 90 millions on:
♣ institutional support;
♣ AIDS;
♣ Immunisation programme;
♣ Public-Private Partnership; and
♣ Health system strengthening.
+ The health Mid Term Expenditures Framework (MTEF)
+ Preparation of the Sector Wide approach Programme (SWAP)

Exact Title of Position held: Economist, Social Sectors

Name/address of employer:	Commission européenne<br>Délégation Damas, Syrie<br>EPSO, Commission Européenne<br>B-11000 Bruxelles, Belgique	*Name and title of supervisor(s):*	Frank Hesske, Chef de Délégation

From:-To: 1/2002 - 1/2005

Brief description of duties and responsibilities:

1. Responsible for the preparation and the management of social development programmes:
♣ Public health (Euro 30 million) this programme was the first reform programme in the health sector in Syria;
♣ Civil society (euro 10 million) under preparation
♣ Other projects in fields of cultural heritage protection (euro 9 million)
2. Technical adviser in health, civil society, NGO and Gender
3. responsible for the technical and financial monitoring of projects

Key achievements:

+ Implementation of the health sector modernisation programme

Exact Title of Position held: Programme Officer/ Economist

Name/address of employer:	Handicap International<br>14, Ave Berthelot,<br>69007, Lyon<br>France	*Name and title of supervisor(s):*	Jean-Baptiste Richardier

From:-To: 9/1997 - 1/2002

Brief description of duties and responsibilities:

1. Responsible for the preparation and implementation of development projects in Chad, Guinea-Bissau, Mali and Mozambique in:
♣ Community-based health
♣ AIDS
♣ Disability
♣ Humanitarian Demining
2. Person in charge of the Technical group on Economics factors

Key achievements:

+ Successful design and implementation of country programmes (5 year prog) for more than euro 7 million.
+ Best financial coverage of all development programmes and excellent diversification of financial resources.
+ Development of an innovative project building development relationship between demining and territorial development.

Please indicate how many people you were responsible for:	250	*May we contact your employer as a reference?*	Yes
Reason for leaving:	looking for career progression		

Exact Title of Position held: Responsable d'opérations, Caucase

Name/address of employer:	Première Urgence<br>9bis rue Georges<br>92250 Garenne-Colombe<br>France	*Name and title of supervisor(s):*	Thierry Mauricet

From:-To: 1/1995 - 3/1997

Brief description of duties and responsibilities:

+ Préparation d'opérations d'aide humanitaire dans le Caucase dans les secteurs suivants:
o Aide alimentaire pour personnes extrêmement vulnérables
o Réhabilitation d'urgence
o Aide intégrée pour personnes extrêmement vulnérables (activités génératrices de revenus)
+ Administration, suivi et supervision de 15 projets (6 millions Euros).
+ Relations avec les donateurs, dont ECHO, Etats membres et ONU (PAM, HCR/OCHA).

Exact Title of Position held: Économiste

Name/address of employer:	CRC-SOGEMA pour:<br>Agence Canadienne de Développement International (ACDI)<br>1111, rue St-Charles Ouest, bureau 454<br>Longueuil, Québec, Canada	*Name and title of supervisor(s):*	Pierre Cholette

From:-To: 9/1994 - 11/1994

Brief description of duties and responsibilities:

+ Analyse complète de l'offre et de la demande du secteur de l'hydraulique souterraine au Niger en vue d'en tirer un diagnostic du marché
+ Évaluation de l’impact socio-économique de différents scénarii de réforme de l'entreprise publique l'Office des Eaux du Sous-sol du Niger en respectant les mesures d'accompagnement des programmes d'ajustement structurel et sectoriel

Key achievements:

analyse de l'offre et la demande de l'eau au Niger

Exact Title of Position held: Économiste (JPO)

Name/address of employer:	PNUD<br>Représentation du PNUD à Bamako<br>BP. 120, Bamako, Mali	*Name and title of supervisor(s):*	Théodore Mpatswenumugabo

From:-To: 9/1991 - 7/1994

Brief description of duties and responsibilities:

+ Administration et gestion de projets dans les domaines du cadrage macro-économique, des finances, du perfectionnement des agents de l?État, de la décentralisation, du processus de démocratisation
+ Préparation du rapport sur la coopération pour le développement, éditions 90,91 et 92/93
+ Préparation du programme de gestion décentralisée du développement comprenant le cadrage macro-économique, la programmation des investissements, le suivi du Programme d'ajustement structurel et le suivi de l'impact de la dévaluation
+ Préparation du projet d'étude nationale de prospective à long terme (NLTPS)
+ Participation à la mise en oeuvre des politiques sectorielles de coopération technique
+ Participation à la préparation de la Conférence de Table Ronde pour le Mali qui s?est tenue en septembre 1994 à Genève

Key achievements:

+ Successful management of 4 development projects in the field of economics
+ Development cooperation Report 1991, 1992-93

Exact Title of Position held: Agent de recherche et de planification socio-économique

Name/address of employer:	Ministère de l'Industrie, du commerce et de la technologie<br>Place d'Youville<br>Québec Canada	*Name and title of supervisor(s):*	Marc Gignac

From:-To: 6/1991 - 9/1991

Brief description of duties and responsibilities:

+ Études économiques de l'Accord de libre échange canado-américain sur le commerce extérieur, l'emploi, l'investissement et la productivité

Key achievements:

five econometric studies on trade and international economics, with application for Quebec Province

Exact Title of Position held: Assistant de recherche

Name/address of employer:	École Nationale d'administration publique<br><br>Ste-Foy,<br>Québec, Canada	*Name and title of supervisor(s):*	Michel Boucher

From:-To: 10/1989 - 6/1991

Brief description of duties and responsibilities:

+ Recherche économétrique sur le bien public et sur la corrélation entre le sport olympique et un bien public

Key achievements:

research paper

Exact Title of Position held: Agent de recherche et de planification socio-économique

Name/address of employer:	Ministère des Affaires internationales<br>525, Boulevard René-Lévesque Est<br>Québec, G1R 5R9<br>Canada	*Name and title of supervisor(s):*	

From:-To: 7/1990 - 9/1990

Brief description of duties and responsibilities:

+ Participation à l'élaboration de politiques et priorités au niveau de la finance et du commerce international pour la nouvelle politique du ministère mise en oeuvre à partir de 1991.

Key achievements:

Analysis of commercial and economic comparative advantage of Quebec Province

Additional Information

Publications, fellowships, etc.:
- The economic cost of homophobia (under submission)
- The return on investment of ending the AIDS epidemic by 2030 (2019)
- A socioecological measurement of homophobia for all countries and its public health impact (2018)
-The Macroeconomic Consequences of Renouncing to Universal Access to Antiretroviral Treatment for HIV in Africa: A Micro-Simulation Model (2012)
-Macroeconomic impact of HIV: the need for better modelling (2010)
- The impact of the Economic crisis on the Response to HIV (2009)

- The response to HIV as a Global public Good (2008)<br>- The long term sustainable financing for HIV: What are the opportunities for Africa ? (2008)

Sean Howell

sean@foundation.lgbt
linkedin.com/in/seanhornet
San Francisco Bay Area

CEO AT LGBT FOUNDATION AND CO-FOUNDER AT HORNET

Education

Economics
Pacific Lutheran University

Graduate **Public Sector Economics**
The Evergreen State College

Certificate **Blockchain Strategy**
Said Business School, University of Oxford

Volunteering

Co-Chair Center for Public Health and Human Rights
Johns Hopkins Bloomberg School of Public Health
Nov 2018 – Present

Board Member **MPact (formerly MSMGF)**
Sep 2018 – Present

Member Board Of Trustees **World Affairs Council**
Jan 2004 – Jan 2008

President & Co Founder
YPIN - Young Professionals International Network
Jan 2004 – Jan 2008

Experience

CEO **LGBT Foundation**
May 2018 – Present Netherlands
The LGBT Foundation is a not-for-profit organization that aims to deliver equality for, and advance the cause of, the LGBT community in countries across the world. We harness the power and potential of blockchain technology – alongside other technological innovations – to foster greater acceptance of the LGBT community, drive positive social change for the community's benefit, and protect vulnerable community members.

Co-Founder, Chairman & President
Hornet Gay Social Networks
2010 – Present San Francisco Bay Area
With Hornet, Gays Can Now Play Safe on Gay Mobile Social Networks

Hornet Networks is gay owned, developer and distributer of the Hornet iPhone and Android Application—a location-based mobile dating and social network that that lets you meet new friends based on criteria you set including geography, share photos quickly by sharing preloaded galleries, and keep notes about people you chat with. Founders and backers include the ranks of LGBT at Microsoft, Linkedin, and Silicon Valley venture technologists. www.hornet.com

Hornet is the virtual "Third Place" of gay social networks. A "First place" is the home. The "second place" is the workplace — where people may actually spend most of their time. Third places, then, are "anchors" of community life and facilitate and foster broader, more creative interaction. All societies already have informal meeting places; what is new in modern times is the intentionality of seeking them out as vital to current societal. Hallmarks of a true "third place": free or inexpensive; while not essential, are important; highly accessible: proximate for many; involve regulars – those who habitually congregate there; welcoming and comfortable; both new friends and old should be found there.

Board of Trustee Member
PFLAG - Parents, Families and Friends of Lesbians and Gays
Jan 2003 – 2005
Parents, Families and Friends of Lesbians and Gays (PFLAG) is a national non- profit organization with over 200,000 members and supporters and over 500 affiliates in the United States. This vast grassroots network is cultivated, resourced and serviced by the PFLAG National Office, located in Washington, D.C., the national Board of Directors and 13 Regional Directors.

Our Vision.
We, the parents, families and friends of lesbian, gay, bisexual and transgender persons, celebrate diversity and envision a society that embraces everyone, including those of diverse sexual orientations and gender identities. Only with respect, dignity and equality for all will we reach our full potential as human beings, individually and collectively. PFLAG welcomes the participation and support of all who share in, and hope to realize this vision.

Anna Yakusik

Phone: 41.78.734.0103 •• E-Mail: annayakusik@gmail.com 10 Rue Emile-Yung •• Geneva 1205, Switzerland
US Permanent Resident

SUMMARY

Technical Officer at UNAIDS, with 10 years of work experience, including more than 7 years related to public health, health financing, and health economics. Designed and managed health economics studies in resource-poor settings, with deep methodological expertise in cost-effectiveness analysis, resource tracking, and economic modeling. Professional experience working with multilateral institutions and diverse research teams. Carried out research projects in Eastern Europe and Central Asia, the Caribbean, Asia, and various countries in sub-Saharan Africa. Experience writing analytical reports and reviews, and leading presentations at the national and international level.

EDUCATION

2010-2012 - MA, Business Administration, European Humanities University, Lithuania. Areas of concentration: public finance and policy, economic analysis and health economics.
2007-2008 – MA, Economics, Brest State Technical University, School of Economics, Belarus. Areas of concentration: economic theory, public economics, public finance and policy, applied econometrics.
2000-2005 - BSc Economics (Honours), Brest State Technical University, School of Economics, Belarus. Areas of concentration: economic history, economic theory, advanced macro and microeconomics, econometrics and economic statistics, computer science.

PROFESSIONAL EXPERIENCE

2013 – Present, Technical Officer, Economics and Evaluation Division, Strategic Information and Evaluation Department, UNAIDS Secretariat, Geneva, Switzerland
Contribute to the development of approaches, normative guidance, tools, and indicators to monitor resources available for HIV&AIDS. Assess health and disease spending data and make recommendations on improving its availability and quality. In collaboration with UNAIDS country teams, liaise with technical partners to improve tracking of health and disease spending data. Develop and assess strategic options, draw strategic implications, and engage in policy discussions on financial sustainability of HIV programs in low- and middle-income countries. Support UNAIDS Secretariat, country teams and regional offices with strategic information activities and quantitative analyses on health and disease financing, leveraging domestic resources, and innovative financing. Contributes to capacity building of UNAIDS regional and country teams. Share best practices and lessons learned across countries. Foster effective relationships and partnerships with country officials and partner agencies; and represent UNAIDS in sustainability and expenditure tracking related forums.

INDEPENDENT CONSULTANCIES

- 2012: Consultancy for the United Nations Development Programme (UNDP), Belarus Country Office. Investment case study with pilot application of the Optima mathematical optimization model for

HIV&AIDS (optimamodel.com) developed by the University of New South Wales (UNSW) with the World Bank. The analysis included strategies for efficient resource allocation and innovative financing mechanisms.

- 2012: Consultancy for the UNDP Belarus Country Office, Programme Implementation Unit of the Global Fund to Fight AIDS, Tuberculosis and Malaria. Resource tracking for HIV, costing. Financial gap analysis for HIV for the standard Global Fund concept note applications.
- 2012: GRM International, Consultancy for Tajikistan. Resource tracking for HIV, costing. Capacity building for the National AIDS Commission. Capacity building for the national technical working group.
- 2011-2012: Consultancy for the UNAIDS China Country Office. Resource tracking for HIV, costing. Capacity building workshops for Chinese Center for Disease Control and Prevention, UNAIDS China, and Beijing University School of Economics (Beijing, Dehong). Facilitation of national and regional level trainings. Capacity building for the national technical working group.
- 2011: Consultancy for the UNAIDS Tajikistan Country Office. Technical report on HIV funding flows in Tajikistan for 2008-2009.
- 2008-2011: Multiple consultancies for the UNAIDS Belarus Country Office. Resource tracking for HIV, costing. Multiple capacity building workshops for the Ministry of Health, the National AIDS Commission, the UNDP Programme Implementation Unit of the Global Fund to Fight AIDS, Tuberculosis and Malaria, civil society and non-governmental organizations. Training of national technical working group. Financial gap analysis for HIV for the standard Global Fund concept note applications. Cost-effectiveness evaluation of needle and syringe exchange programmes for people who inject drugs.
- 2010: Consultancy for the International HIV/AIDS Alliance, Ukraine Country Office. Resource tracking for HIV. Capacity building workshops for the National AIDS Commission, and Ukraine Country Office of the International HIV/AIDS Alliance.
- 2009-2011: Multiple consultancies for the UNDP, Programme Implementation Unit of the Global Fund, AIDS, Tuberculosis and Malaria grants, Tajikistan Country Office. Resource tracking for HIV, and TB. Multiple capacity building workshops for the National AIDS Commission, the UNDP Programme Implementation Unit of the Global Fund to Fight AIDS, Tuberculosis and Malaria, the TB Control Programme, civil society and non-governmental organizations. Budgeting of HIV national strategic plan. Financial gap analysis for HIV, and TB for the standard Global Fund concept note applications.
- 2009, 2010: UNAIDS Secretariat Geneva, Consultancy for Belarus. Evaluation of the impact of the global economic crisis on HIV prevention and treatment programs in Belarus.

TEACHING EXPERIENCE

2006 – 2009 Brest State Technical University, School of Economics, Belarus. University instructor for undergraduate students at the School of Economics. Coursework: Business Law, Applied Taxation, Public Finance, Mathematical Statistics.

SELECTED PUBLICATIONS

- "Financing and Costing." In: Consolidated Strategic Information Guidelines for HIV in the Health Sector, 63-64, 205207, 274-275. Geneva: World Health Organization (WHO). 2015 http://apps.who.int/iris/bitstream/10665/164716/1/9789241508759_eng.pdf?ua=1&ua=1
- "The Financing Lesson." In: How AIDS Changed Everything — MDG6: 15 Years, 15 Lessons of Hope from the AIDS Response, 187-221. Geneva: UNAIDS. 2015

- "Target 6. Close the Global Aids Resource Gap by 2015 and Reach Annual Global Investment of US$22–24 Billion in Low- and Middle-income Countries." In: Global AIDS Response Progress Reporting 2015, 88-90, 110-117. Geneva: UNAIDS. 2015 http://www.unaids.org/sites/default/files/media_asset/JC2702_GARPR2015guidelines_en.pdf
- Duo Shan, Jiangping Sun, Anna Yakusik, et al. Total HIV/AIDS Expenditures in Dehong Prefecture, Yunnan Province in 2010: The First Systematic Evaluation of Both Health and Non-Health Related HIV/AIDS Expenditures in China. PLOS ONE. 2013 http://journals.plos.org/plosone/article?id=10.1371/journal.pone.0068006
- David P. Wilson, Anna Yakusik, et al. HIV resource needs, efficient allocation and resource mobilization for the Republic of Belarus. UNAIDS, UNDP, UNSW, and Abt Associates. 2013 http://optimamodel.com/pubs/belarusreport.pdf
- "Close the Global AIDS Resource Gap." In Global Report: UNAIDS Report on the Global AIDS Epidemic 2013, 68-77. Geneva: UNAIDS. 2013 http://www.unaids.org/sites/default/files/media_asset/UNAIDS_Global_Report_2013_en_1.pdf
- Smart Investments. Geneva: UNAIDS. 2013 http://www.unaids.org/sites/default/files/media_asset/20131130_smart-investments_en_1.pdf Anna Yakusik, et al.
  National AIDS Spending Assessment in Dehong Prefecture of Yunnan Province, China. China CDC, UNAIDS, Beijing University. 2012 http://www.unaids.org/sites/default/files/documents/china_2010_en.pdf
- Anna Yakusik, et al. Resource Flows and Levels of Spending for the Fight against Tuberculosis in Tajikistan, 2010. Tajikistan TB Control Programme, UNDP. 2012. Available upon request.
- Anna Yakusik, et al. National AIDS Spending Assessment in Belarus for the period of 2008-2012. Belarus national AIDS commission, UNAIDS, UNDP. 2012. http://www.unaids.org/sites/default/files/documents/belarus_20082011_en.pdf
- Valentina I Kachan, Alena I Tkachova, Eleanora Gvozdeva, Ilona Urbanovich, Anna Yakusik, Peter Amico, and Carlos Avila. Resource flows and levels of spending for the response to HIV/AIDS in Belarus. BMC research notes. 2011 http://www.ncbi.nlm.nih.gov/pmc/articles/PMC3156755/
- Anna Yakusik, et al. National AIDS Spending Assessment in Tajikistan for the period of 2008-2009. Tajikistan national AIDS commission, UNAIDS, UNDP. 2011. http://www.unaids.org/sites/default/files/en/media/unaids/contentassets/dataimport/pub/report/2008/NASA_TAJIKI STAN_2008-2009_en.pdf

LANGUAGES AND SKILLS

Fluent English and native Russian; intermediate French
MS Office, Stata, Matlab

Bruno Ventelou

AMSE
5/9 bd Bourdet
Marseille
13001
FRANCE

+33 (0)4 13 55 25 82
bruno.ventelou@univ-amu.fr
BORN 1967

Position

January 2004 - present)

Aix Marseille School of Economics (AMSE) ; CNRS, Aix-Marseille University, France
Research professor

Member of Collège des Economistes de la Santé, Member of Commission des comptes de la Santé, Laureate of Programme AVENIR de l'Inserm, advisor at Observatoire Régional de la Santé PACA, advisor at MGEN research council.

Research

Themes

Health Economics ; Macroeconomics ; History of Keynesian Economics

Areas of expertise in health economics:

- **Production of health, health behaviours & policy interventions**
- **Supply of health services**
- **Health care financing & expenditures**
- **Evaluation of policy, programs and health system performance**

Recent Publications

1. Cortaredona, S., & **Ventelou, B**. (2017). The extra cost of comorbidity: multiple illnesses and the economic burden of non-communicable diseases. *BMC Medicine*, 15(1), 216.
2. Treibich, C., Lescher, S., Sagaon-Teyssier, L., and **Ventelou, B**. (2017). The expected and unexpected benefits of dispensing the exact number of pills, *PLoS-ONE*, 12(9) : e0184420.
3. Nay O., Béjean S., Benamouzig, D., Bergeron H., P. Castel., & **Ventelou B**. (2016). Achieving universal health coverage in France: policy reforms and the challenge of inequalities. *The Lancet*, online: May 2, 2016, Vol 387, No. 10034, p2236–2249, 28 May 2016
4. Tchewonpi-Kankeu, H., & **Ventelou, B**. (2016). Socioeconomic inequalities in informal payments for health care: An assessment of the 'Robin Hood' hypothesis in 33 African countries. *Social Science & Medicine*, 151(February), 173-186.
5. Weeks, W. B., Jardin, M., Dufour, J. C., Paraponaris, A., & **Ventelou, B.** (2014). Geographic Variation in Admissions for Knee Replacement, Hip Replacement, and Hip Fracture in France: Evidence of Supplier-induced Demand in For-Profit and Not-for-Profit Hospitals. *Medical care*, 52(10), 909-917.
6. Arrighi, Y., Abu-Zaineh, M., & **Ventelou, B**. (2015). To Count or Not to Count Deaths: Reranking Effects in Health Distribution Evaluation. *Health economics*, 24(2), 193-205.
7. Woode, M. E., Nourry, C., & **Ventelou, B**. (2014). Childhood preventive care, adult healthcare and economic growth: The role of healthcare financing. *Economics Letters*, 124(1), 41-47.

Books:

(1) *Millennial Keynes : **The Origins, Development and Future of Keynesian economics***, M.E. Sharpe Publisher, New York + ed Paperback. Traduction et préface de G. Nowell, 2004.

(2) ***Au delà de la rareté, la croissance économique comme construction sociale***, Albin-Michel, Paris, septembre 2001. Sélectionné pour les journées 2001 du *Livre d'Economie* au Sénat.

02/06/19

Michael Wallis ROSS

Address Program in Human Sexuality,
Department of Family Medicine and Community Health,
University of Minnesota,
1300 South 2nd Street, Minneapolis MN 55454, U.S.A.

Tel: Office: 612-624-4749; Secretary: 612-625-1500.

Citizenships New Zealand, Australia,United States of America.

Education

Massey University, New Zealand (Faculty of Arts: in Psychology)	B.A. and *(1973)* B.A.(Hons) *(1974)*
University of the State of New York, Albany, New York, U.S.A (Faculty of Science: in Medical Sociology)	B.S. *(1976)*
Victoria University of Wellington, New Zealand (Faculty of Arts: in Social-Clinical Psychology)	M.A. *(1975)*
University of New England, Australia (Faculty of Education: in Adult Education)	Diploma in Tertiary Ed. *(1984)*
University of Melbourne, Australia (Faculty of Science: in Health Psychology)	Ph.D. *(1980)*
University of Adelaide, Australia (Faculty of Medicine: in Epidemiology)	M.P.H. *(1989)*
University of New South Wales, Australia (Faculty of Medicine: in Health Personnel Education)	M.H.P.Ed. *(1991)*
Prince of Songkla University, Thailand (Faculty of Medicine: in Tropical Clinical STDs)	Diploma in STDs *(1992)*
Malmö University, Sweden. (Faculty of Medical Sciences: in Health and Society)	*Medicinae Doctor:* M.D. (2006)

	University of Cambridge, UK<br>(Faculty of Law: in Prison Studies)	Diploma in<br>Applied Criminology<br>(with Commendation)<br>*(2003)*
	University of Cambridge, UK<br>(Faculty of Law: in Applied Criminology)	M.St. *(2004)*<br>(Prize for top student)
**Post Doctoral**	Institute of Sociology, University of Helsinki, Finland	*(1978, 1980)*
**Employment**	**Professor and Joycelyn Elders Chair of Sexual Health Education**<br>**Department of Family Medicine and Community Health**<br>**University of Minnesota**	**2014 –**
	Professor (with tenure)<br>School of Public Health<br>Center for Health Promotion and Prevention Research<br>The University of Texas at Houston	1993 – 2014
	and<br>Professor (Cross-appointment)<br>Infectious Diseases, School of Medicine<br>The University of Texas at Houston	1996 –2014
	Director, University of New South Wales Unit<br>National Centre in HIV Social Research<br>School of Community Medicine<br>(rank of Associate Professor)	1990 - 1993
	*Co-ordinator, WHO International Training Courses<br>on Counseling and Risk Reduction in HIV Infection<br>and Disease, WHO Regional Training Centre,<br>University of New South Wales	1990 - 1993
	National Health and Medical Research Council<br>Senior Research Fellow<br>The Sydney Hospital	1989 - 1990
	*Occasional World Health Organisation Consultant,<br>Global Programme on AIDS<br>Geneva	1989 -
	Director, Research, Evaluation and Epidemiology<br>STD/AIDS Services<br>South Australian Health Commission	1988 - 1989
	STD/AIDS Programme Co-ordinator	

South Australian Health Commission 1985 - 1988

Senior Demonstrator (Grade 2) in Psychiatry
Flinders University of South Australia 1978 - 1985

Tutor in Psychology
University of Melbourne, Australia 1977 - 1978

*Unpaid co-appointments

Honorary Positions

Clinical Lecturer in Venereology
University of Sydney 1989 - 1993

Visiting Fellow
National Drug and Alcohol Research Centre UNSW 1991 - 1993

Clinical Senior Lecturer in Psychiatry and
Community Medicine
Flinders University of South Australia
Medical School 1985 - 1989

Senior Clinical Psychologist (PSC-3)
Flinders Medical Centre 1978 - 1989

Visiting Lecturer, Glenside Psychiatric Hospital 1980 – 1989

Chair, Fellows Committee, Division 44
American Psychological Association 2000 – 2003

Honorary Professor of Health Sciences, University of Sydney 2006 –

Docent, Faculty of Health and Society
Malmö University, Sweden 2009 –

Memberships

Fellow, New Zealand Psychological Society
Fellow, British Psychological Society
Fellow, American Psychological Association (Division 44)
Fellow, Royal Society of Arts
Fellow, Royal Society of Public Health
Fellow, Society of Antiquaries of Scotland
Fellow, Society for the Scientific Study of Sexuality; (International Board Member, 1991-1993; Midcontinent region Board member, 1998-1999; President

Elect, 1999; President, 2000-2001, past-president, 2001-2002)
Honorary Fellow, Royal Australasian College of Physicians, Chapter of Sexual Health Medicine
Liveryman, Worshipful Company of Scriveners of London, and Freeman of the City of London

Awards and Honours

Faculty of Arts Travelling Scholarship,
University of Melbourne, 1977
(to Stockholm and Göteborg Universities for doctoral research).

Faculty of Arts Travelling Scholarship, 1978
(to University of Helsinki for post-doctoral research).

Hugo Beigel Award of the Society for the Scientific Study of Sex, U.S.A. (annual international award for the most important contribution to sexuality research), 1980.

Delivered first Michael Q. Petersen Memorial Lecture to American Physicians for Human Rights, Chicago, August 1984, on "Social and Political Aspects of Homosexual Health Care".

Keynote Address, "Homosexuality beyond Disease" conference, Amsterdam, December 1987, on "The Future of the Study of Homosexuality".

The Newman Award for Excellence in AIDS Research, Australian National Council on AIDS (annual award for research excellence), 1989.

Keynote Address, International Academy of Sex Research, Uppsala, Sweden, August 1990, on "Social and Cultural Influences on Male Homosexual Behaviour".

Plenary Address, Society for the Scientific Study of Sex, Annual Conference, Chicago, November 1993, on "Attitudes toward Condoms in Homosexual Men".

US Surgeon-General’s Exemplary Service Award, 2001, for co-writing the “Surgeon-General’s Call to Action to Promote Sexual Health and Responsible Sexual Behavior”.

Alfred C. Kinsey Award, Society for the Scientific Study of Sexuality, 2004, for scientific achievement in the field of sexology.

Distinguished Scientific Achievement Award, 2005, Society for the Scientific Study of Sexuality, for contributions which have advanced the understanding of human sexual behavior and promoted the study of sexuality.

Current Licensure/Registration

Chartered Psychologist, United Kingdom Registration # 16754 (since 1988)

Research Grants

Australia:

University of Melbourne research grants (2)	1977	$300
	1978	$500
Flinders University research grants (3)	1980	$2,500
	1981	$420
	1982	$860
Glenside Hospital Research Foundation grant (Measurement of Depression)	1980	$1,000
South Australian Health Commission grant (Psychoneuroimmunology in male homosexuals: with Dr. P.A. Drew)	1983	$25,720
National Health and Medical Research Council (Precursors of AIDS in Male Homosexuals: with Dr. P.A. Drew)	1984	$23,500
National Health and Medical Research Council (Assessment of AIDS Risks and the Cognitive, Informational and Behavioural components of AIDS)	1986	$83,864
South Australian Health Commission Grant (Attitudes to condom use in heterosexual men and women: with Dr S. Chapman)	1986	$5,000
National Health and Medical Research Council (Knowledge of AIDS and risk behaviours in four ethnic minority groups: with Dr K. Rigby et al)	1988	$72,900
National Health and Medical	1989	$42,300

Research Council (Effects of meditation on the immune system of HIV infected men: with Drs J. Condon & J. Need)		
Newman Award for AIDS Research	1989	$12,500
Committee on AIDS Research Grants (Situational contributors to and rationalisations used to justify unsafe sex: with Dr R. Gold & Mr B. Parnell)	1990	$47,400
World Health Organisation (Indirect methods of enumeration of groups particularly at risk of HIV infection in an urban environment: with Drs R. Taylor, B. Donovan, J. Kaldor, & Prof. J. Darroch)	1990	$70,060
Committee on AIDS Research Grants (Decision making and determinants of risk behaviours for HIV infection)	1991	$44,059
Committee on AIDS Research Grants (Emotional Well-being of people bereaved by AIDS: with Dr B. Kelly & Prof. B. Raphael)	1991	$51,265
Committee on AIDS Research Grants (National Injecting Drug and AIDS Study: with Drs A. Wodak, B. Monheit, Prof. D. Hawks, Ms W. Loxley, & Mr A Buzolic)	1991	$254,270
Research into Drug Abuse Grants (Determinants of risk-taking among methadone maintenance clients: with Prof. N. Heather, Prof W. Hall, & Dr S. Darke)	1992	$94,812
Committee on AIDS Research Grants (National Injecting Drug and AIDS Study)	1992	$92,000

U.S.A:

National Institutes for Health: HIV/AIDS Risks and Education Component, Research Centers for Minority Institutions Grants (through Texas Southern University. James Essien, Co-Principal Investigator) $252,000/5 years (20% time)

American AIDS Foundation: HIV Prevention Case Management for Gay/Lesbian Street Youth (through Houston Institute for the Protection of Youth (Tracy Brown, Co-Principal Investigator) $79,365/2 years (5% time)

Health Resources Services Administration: Learning, Empowerment Advocacy Participation (Jim Halloran, Co-Principal Investigator) $55,532/1 year (5% time)

Health Resources Services Administration: Evaluation of Texas/Oklahoma AIDS ETC Dental Training Project, Consultant (Deborah Brimlow, Principal Investigator) $10,000/1 year

Health Resources Services Administration: AIDS Education & Training Center for Texas and Oklahoma, Evaluation Consultant (Richard Grimes, Principal investigator) $13,346=10% time/5 years

City of Houston - Health & Human Services: Evaluation of City's HIV Prevention Planning Process (Co-Evaluator with Deborah Scott) $15,000/2 years

National Institute for Drug Abuse: Tuberculosis Spread in Drug Users in Houston, TX (James M. Musser, Baylor College of Medicine, Principal Investigator) $5,695,700/5 years (5% time)

Centers for Disease Control: STD/HIV Behavioral Training Center (jointly with Dallas County Health Department) (Michael W. Ross, Principal Investigator) $1,300,000/5 years (25% time)

Centers for Disease Control: Syphilis control in Houston (Michael W. Ross, Principal Investigator) $2,000,000/5 years (30% time)

Texas Commission on Alcohol and Drug Abuse: STDs in crack users (Michael W. Ross, Principal Investigator) $28,000/1 year

Texas Commission on Alcohol and Drug Abuse: STDs in drug users in treatment (Michael W. Ross, Principal Investigator) $32,000/1 year

National Institute of Drug Abuse: Positive Choices Research Project (Mark L. Williams, Principal Investigator) $1,835,000/5 years (20% time)

National Institutes of Health: Social network characteristics of a highly active HIV/STD core group (Mark L. Williams, Principal Investigator) $1,669,000/5 years (20% time)

National Institutes of Health: Videotape-based HIV prevention (James Essien, Principal Investigator). Subcontract $535,000/4 years (15% time)

Centers for Disease Control: Behavioral Surveillance for gonorrhea in Houston (Michael W. Ross, Principal Investigator) $447,000/3 years (40% time)

CDC (through Association of Schools of Public Health): Identifying non-gay identifying men who have sex with men on the Internet (Michael W. Ross, Principal Investigator). $400,000/2 years (20% time)

Centers for Disease Control: Tanzanian AIDS Prevention Project (Mark L. Williams, Principal Investigator) $167,195/2 years (9% time).

National Institutes of Health: HIV, Risk Behaviors and Stigma in in High Risk Tanzanian Men (Michael W. Ross, Principal investigator). $256,017/2 years (15% time)

<u>University Teaching</u>

Flinders University - School of Medicine

1978 - 1985	Co-ordinator, Psychosocial-Psychobiological Systems Course, Year III Medicine, Flinders University
1979 - 1988	Co-ordinating Lecturer, Introduction to Sexual Medicine, Core Topic, Year IV Medicine, Flinders University
1979 - 1980	Bridging courses in Sexual Medicine, Years IV to Vl Medicine, Flinders University
1980 - 1988	Lecturer to psychiatric trainees, Year I & Year III Boards for Royal Australian and New Zealand College of Psychiatrists (co-ordinator of Clinical Psychology course).
1979 - 1985	Electives offered in sexual medicine (with Dr. J.A.Need), psychiatry (gender problems and transsexualism), Flinders University
1982 - 1985	Co-ordinator (with Dr. M. McLaughlin), General Issues in Medicine, Year IV Medicine, Flinders University

University of New South Wales - School of Community Medicine

1989 - 1993	Occasional lectures, MMed(Ven) and MPH courses, University of Sydney & MCH course, University of New South Wales

University of Texas - School of Public Health

1993 - 1996	Addictive Behaviors (Co-taught with Dr David Martin), 4 credit hours

1994 - 2000	Advanced Theory in Health Promotion (Co-taught with Drs Alfred McAlister and María Fernández-Esquer), 3 credit hours
1994 -	Genital, Sexual and Reproductive Public Health (Sole teacher), 3 credit hours
1995 - 1999	Social Theory and Health (Co-taught with faculty of Behavioral Sciences and Management and Policy Sciences), 2 credit hours
2000 - 2003	International Health Promotion (Co-taught with Drs Alfred McAlister and María Fernández-Esquer), 3 credit hours
2006 - 2014	AIDS and Africa (Co-taught with Dr Sheryl McCurdy), 3 credit hours
2007 - 2014	Introduction to Health Promotion and Behavioral Sciences (Co-taught with Dr Alfred McAlister), 3 credit hours

University of Minnesota – School of Medicine

2015 –	MS-1 Sexual Health
2017 –	MS-4 LGBT Elective

Clinical Duties

1979 - 1988	Coordinator, Gender Clinic, Flinders Medical Centre
1985 - 1988	Coordinator, AIDS Clinic, South Australian Health Commission

Committees
Flinders University
University

1980 - 1982	Academic Committee
1980 - 1982	Animal Experimentation Ethics Review Committee

Medical School

1979 - 1985	Third Year Committee
1979 - 1985	Third Year Examinations Board
1979 - 1989	Fourth Year Committee
1979 - 1989	Fourth Year Examinations Board
1980 - 1985	Standing Committee

1980 - 1988	Research Committee
1980 - 1985	Admissions Committee
1985 - 1988	Curriculum Evaluation Committee

University of Texas - School of Public Health

1993 - 2001	Admissions Committee
1994 - 2000	Distance Learning Working Group<br>(and Subcommittee for Course Design and Presentation)
1995-1997,<br>2000-2002	Convenor (Head of Department), Health Promotion/Behavioral Sciences
1999 -	Committee, Delta Omega (University of Texas chapter)<br>(Public Health honors society)

Houston Health Science Center

1996 - 1998	Infectious Diseases Committee
1997- 2002	Institutional Biosafety Committee

Houston Community Service

1994- 2000	Member, AIDS Care Team, St Anne's Catholic Church
1994 - 1997	Board Member, Hope is Victory (services to African American people with HIV disease)
1995 - 1997	Harris County (Houston) Ryan White Planning Council<br>Case Management subcommittee<br>Cultural Sensitivity Training workgroup<br>Client Satisfaction Survey workgroup<br>Minority Sensitivity and RFP Review workgroup
1995 - 2005	Board, Kolbe Project (Franciscan NGO for people with HIV disease)
1995 - 2014	Community Research Risk Protection Committee, Montrose/Legacy Clinic<br>(Institutional Review Board for Houston Clinical Research Network)
1997 - 2014	Member, Research Advisory Group, Houston Area Women’s Center
1997 - 2006	Board Member, Saving Lives through Alternate Options (Minority public health interventions CBO) (Board chair 2001 - 2006)

2006 - 2013	Trustee, Christus - Stehlin Foundation for Cancer Research
2009 - 2012	Member, Houston-Harris County Office of Drug Policy Advisory Committee
2011 - 2014	Board Member, LIVE (NGO working to reduce HIV-related stigma)
2014 - 2017	Board member, Human Research Committee, American Psychological Association (APA)
2012 -	Member National Institute of Health Study Section ZRG1 AARR-C(22)

Australia: State & National

1979 - 1993	Secretary, Australian and New Zealand Medical Committee on Transsexualism
1983 - 1989	Secretary, South Australian Advisory Committee on AIDS
1983 - 1984	Secretary, National Venereology Council of Australia
1983 - 1989	Delegate to Council, National Venereology Council of Australia
1985 - 1987	Member, South Australian Animal Welfare Advisory Committee
1985 - 1988	Member, National AIDS Task Force
1986 - 1988	Member, National Advisory Committee on AIDS
1988 - 1990	Member, National AIDS Forum

International

1989 - 1990	Member, WHO Technical Working group on Bisexuality and HIV/AIDS
1989 - 1990	Member, WHO Technical Working Group on Responses of Health Workers to HIV/AIDS
1993 - 1997	Chair, HIV/AIDS Committee, Society for the Scientific Study of Sexuality
1997 - 1999	Chair, Publications Committee, Society for the Scientific Study of Sexuality
2000 - 2001	President, Society for the Scientific Study of Sexuality (President-elect 1999, Past president, 2002)

**Editorial**	Board of AIDS Care<br>(American Editor, 1994 - 2006)	1990 - 2010

Board of Journal of Psychology and Human Sexuality (from 2007, becomes International Journal of Sexual Health) 1989 –

Board of Journal of Homosexuality, 1983 -

Board of Sexological Review 1992 - 2000

Board of Venereology 1992 - 2001

Board of International Journal of Intercultural Relations 1992 - 2005

Board of International Journal of Transgenderism 1997-

Board of book series Contemporary Perspectives on Lesbian, Gay and Bisexual Psychology, APA Books 2001-

Board of Counseling, Psychotherapy and Health 2004-

Board of Journal of Correctional Health Care 2005-

Board of Psychology and Sexuality 2009-

Chair, Publications Committee, Society for the Scientific Study of Sexuality (responsible for oversight of Journal of Sex Research and Annual Review of Sex Research) 1997-2000.

Board of Social Science and Medicine 2003-2006

Board of Sexuality Research and Social Policy 2005-2012

Board of Journal of LGBT Health Research 2005-2009

Aviation Training:
Sydney Aerobatic School
Licences -- Australian and US Private Pilot, land/single, both current
Aircraft ratings -- Avions Pierre Robin 2160 Aerobin, Bellanca 8KCAB Super Decathalon; and Pitts S-2A Special (all with unrestricted aerobatic ratings)

Publications

Impact Analytics (Manifold, 12/20/18)

h-Index	H(fl)-Index	Total Publications	First/Last Author Publications	Total Citations	First/Last Author Citations

**50**	**39**	**470**	**286**	**9385**	**5285**

PEER REVIEWED PUBLICATIONS

Journal Papers

1. Ross, M.W. Relationship between sex role and sex orientation in homosexual men. *New Zealand Psychologist*, 1975, *4*(1), 25-29.
2. Ross, M.W. Paradigm lost or paradigm regained? Behaviour modification and homosexuality. *New Zealand Psychologist*, 1977, *6*(1), 42-51.
3. Ross, M.W. Societal reaction theory: a phenomenological approach. *Melbourne Psychology Reports*, 1977, *42*, 1-17.
4. Ross, M.W., Rogers, L.J., & McCulloch, H. Stigma, sex and society: a new look at gender differentiation and sexual variation. *Journal of Homosexuality*, 1978, *3*(4), 315-330.
5. Ross, M.W. The relationship of perceived societal hostility,conformity and psychological adjustment in homosexual males. *Journal of Homosexualitv*, 1978, *4*(2), 157-168.
6. Ross, M.W. The ethics of animal experimentation: control in practice. *Australian Psychologist*, 1978, *13*, 375-378.
7. Ross, M.W. Some possible relationship between brain structure and behaviour in Cetaceans. *Melbourne Psychology Reports*, 1978, *48*, 1-18.
8. Ross, M.W., & Talikka, A. Finland and homosexuality. *Psychiatric News*, 1978, *13*(14), 2 & 10.
9. Ross, M.W., & Talikka, A. Societal pressure, the homosexual and the role of psychiatry. *Nordisk Psykiatrisk Tidskrift*, 1978, *32*, 543-546.
10. Ross, M .W. Brain and behaviour in Cetaceans. *Commission of Inquiry into Whales and Whaling*, Australian Federal Government, 1978, *2*, 57-93.
11. Ross, M.W. Bisexuality: fact or fallacy? *British Journal of Sexual Medicine*, 1979, *6*(45), 49-50.
12. Ross, M.W. Homosexuality and venereal disease. *British Journal of Sexual Medicine*, 1979, *6*(46), 32-33.
13. Ross, M.W. Heterosexual marriage of homosexual males: some associated factors. *Journal of Sex and Marital Therapy*, 1979, *5*(2), 142-151.
14. Ross, M.W., & Stålström, O.W. Exorcism as psychiatric treatment: a homosexual case study. Archives of Sexual Behavior, 1979, 8(4), 379-383.
15. Ross, M.W., & Talikka, A. Homosexual labelling and cultural control. Psychiatric Opinion, 1979, 16(10), 31-33.
16. Wålinder, J., Lundström, B., Ross, M.W., & Thuwe, I. Transsexualism: incidence, prevalence and sex ratio. *Proceedings of the Sixth International Gender Dysphoria Symposium*, 1979.
17. Sidanius, J., Ekehammar, B., & Ross, M.W. Comparisons of sociopolitical attitudes between democratic societies. *International Journal of Psychology*, 1979, *14*, 225-240.
18. Ross, M.W. Why people change their sex: an empirical view. *Australian Left Review*, 1979, October, *71*, 21.
19. Ross, M.W. Psychological malpractice and the anti-cult battle: the brain awash. *Australian Journal of Social Issues*, 1979, *14*, 201-210.

20. Ross, M.W. Retrospective distortion in homosexual research. *Archives of Sexual Behavior*, 1980, *9*, 523-531.
21. Orr, K., & Ross, M.W. Homosexuality in clinical and biblical context. *Journal of Christian Education*. 1980, *69*, 37-48.
22. Andrew, G.M., & Ross, M.W. A short form of the Bem Sex Role Inventory. *Journal of Psychiatric Treatment and Evaluation*, 1981, *3*, 563-566.
23. Ross, M.W., Wålinder, J., Lundström, B., & Thuwe, I. Cross-cultural approaches to transsexualism: a comparison between Sweden and Australia. *Acta Psychiatrica Scandinavica*, 1981, *63*, 75-82.
24. Ross, M.W. The ethics of experiments on higher animals. *Social Science and Medicine*, 1981, *15F*, 52-61.*
25. Ross, M.W. Attitudes of male homosexuals to venereal disease clinics. *Medical Journal of Australia*, 1981, *2*, 670-671.
26. Ross, M.W. Social factors in homosexually acquired venereal disease: a comparison between Sweden and Australia. *British Journal of Venereal Diseases*, 1982, *58*, 263-268.
27. Ross, M.W. Some effects of heterosexual marriage on homosexual desire. *Australian Journal of Sex, Marriage and Family*, 1982, *3*(1), 25-29.
28. Ross, M.W. The ethics of animal experimentation in medicine. *New Doctor*, 1982, *24*, 12-13.
29. Badcock, K.A., & Ross, M.W. Neuropsychological testing of Australian Aboriginals. Australian Psychologist, 1982, 17, 297-299.
30. Ross, M.W., Campbell, R.L., & Clayer, J.R. New inventory for measurement of parental rearing patterns: an English form of the EMBU. Acta Psychiatrica Scandinavica, 1982, 66, 499-507.
31. Ross, M.W. Societal relationships and gender role in homosexuals: a cross-cultural comparison. *Journal of Sex Research*, 1983, *19*, 273-288.
32. Ross, M.W. Clinical profiles of Hare Krishna devotees. *American Journal of Psychiatry*, 1983, *140*, 416-420.
33. Ross, M.W., Kalucy, R.S., & Morton, J.E. Locus of control in obesity: predictors of success in a jaw-wiring programme. *British Journal of Medical Psychology*, 1983, *56*, 49-56.
34. Ross, M.W. Counselling the homosexual patient. *Patient Management*, 1983, *7*(7), 117-123.
35. Ross, M.W. Mental health and membership of the Hare Krishnas: a case study. *Australian Psychologist*, 1983, *18*, 128-129.
36. Ross, M.W. Homosexuality and social sex roles: a re-evaluation. *Journal of Homosexuality*, 1983, *9*(1), 1-6.*
37. Ross, M.W. Femininity, masculinity and sexual orientation: some cross cultural relationships. *Journal of Homosexuality*, 1983, *9*(1), 27-36.*
38. Hafner, R.J., & Ross, M.W. Predicting the outcome of behavior therapy for agoraphobia. *Behavior Research and Therapy*, 1983, *21*, 375-382.
39. Ross, M.W., Clayer, J.R., & Campbell, R.L. Parental rearing patterns and suicidal thoughts. *Acta Psychiatrica Scandinavica*, 1983, *67*, 429-433.
40. Ross, M.W. Predictive value of selection interviews in success in first year medicine. *Medical Journal of Australia*, 1983, *2*, 423-424.
41. Adcock, N.V., & Ross, M.W. Early memories, early experiences and personality. *Social Behaviour and Personality*, 1983, *11*, 95-100.
42. Ross, M.W., Clayer, J.R., & Campbell, R.L. Dimensions of child-rearing practices: factor structure of the EMBU. *Acta Psychiatrica Scandinavica*, 1983, *68*, 476-483.

43. Campbell, R.L., Clayer, J.R., & Ross, M.W. An Australian standardisation of the Eysenck Personality Questionnaire. *Australian Council for Educational Research Bulletin*, 1983, *34*, 22-25.
44. Ross, M.W., & Need, J.A. Teaching sexual medicine: the Flinders curriculum. *British Journal of Sexual Medicine*, 1984, *11*, 94-96.
45. Ross, M.W. Problems with research on Hare Krishna Devotees. American Journal of Psychiatry, 1984, 141,144.
46. Ross, M.W. Intelligence testing in Australian Aboriginals. *Comparitive Education*, 1984, *20*, 371-375.
47. Clayer, J.R., Campbell, R.L., & Ross, M.W. Parental rearing and transient thought disorder in young adults. *Psychopathology*, 1984, *17*, 9-16.
48. Ross, M.W. Designing sexual medicine courses: a model. *Medical Education*, 1984, *18*, 24-30.
49. Ross, M.W. Sexually transmitted diseases in homosexual men: a study of four societies. *British Journal of Venereal Diseases*, 1984, *60*, 52-55.
50. Ross, M.W., Goss, A.N., & Kalucy, R.S. The relationship of panic-fear to anxiety and tension in jaw wiring for obesity. *British Journal of Medical Psychology*, 1984, *57*, 67-69.
51. Ross, M.W. Social and psychological predictors of STD infection in homosexual men: a study of four countries. *British Journal of Venereal Diseases*, 1984, *60*, 110-113.
52. Hafner, R.J., & Ross, M.W. Agoraphobia in women: factor analysis of symptoms and personality correlates of factor scores in a clinical population. *Behavior Research and Therapy*, 1984, *22*, 441-444.
53. Ross, M.W. Predictors of partner numbers in homosexual men: psychosocial factors in four societies. *Sexually Transmitted Diseases*, 1984, *11*, 119-122.
54. Ross, M.W., & Stålström, O.W. Attitudes to venereal disease clinics in Finnish homosexual men. *European Journal of Sexually Transmitted Diseases*, 1984, *1*, 169-171.
55. Clayer, J.R., Bookless, C., & Ross, M.W. Neurosis and conscious symptom exaggeration: its differentiation by the Illness Behaviour Questionnaire. *Journal of Psychosomatic Research*, 1984, *28*, 237-241.
56. Forrest, Q.C., & Ross, M.W. Predictors of neurosis in migrants: Greek-ltalian and male-female differences. *American Journal of Social Psychiatry*, 1984, *4*, 52-56.
57. Ross, M.W. Beyond the biological model: new directions in homosexual and bisexual research. *Journal of Homosexuality*, 1984, *10*(3&4), 63-70.
58. Clayer, J.R., Ross, M.W., & Campbell, R.L. Child rearing patterns and dimensions of personality. *Social Behaviour and Personality*, 1984, *12*, 153-156.
59. Ross, M.W. Psychotherapy as a model for interpersonal dynamics in tertiary teaching. *Higher Education*, 1984, *13*, 717-729.
60. Ross, M.W. Selection of medical students: problems and prospects. *Australian Family Physician*, 1985, *14*, 1076-1081.
61. Ross, M.W. Actual and anticipated societal reaction to homosexuality and adjustment in two societies. *Journal of Sex Research*, 1985, *21*, 40-55.
62. Ross, M.W. Sexual practices as risk factors for sexually transmitted diseases in homosexual men. *European Journal of Sexually Transmitted Diseases*, 1985, *2*, 159-162.
63. Ross, M.W. Psychosocial factors in admitting to homosexuality in sexually transmitted disease clinics. *Sexually Transmitted Diseases*, 1985, *12*, 83-86.
64. Ross, M.W., Drew, P.A., & Beal, R.W. Characteristics of homosexual men who donate blood. *Medical Journal of Australia*, 1985, *142*, 343-344.

65. Ross, M.W. Interventions to minimise AIDS. *Medical Journal of Australia*, 1985, *142*, 279-280.
66. Perris, C., Arrindell, W.A., Perris, H., van der Ende, J., Maj, M., Benjaminsen,S., Ross, M.W., Eisemann, M., & del Vecchio, M. Cross-national study of perceived parental rearing behaviour in healthy subjects from Australia, Denmark, Italy, The Netherlands and Sweden: pattern and level comparisons. *Acta Psychiatrica Scandinavica*, 1985, *72*, 278-282.
67. Ross, M.W. Understanding the Homosexual Patient. *Patient Management*, 1985, *9*, 15-25.
68. Ross, M.W. Mental health in Hare Krishna devotees: a longitudinal study. *American Journal of Social Psychiatry*, 1985, *5*, 65-67.
69. Ross, M.W. Social and behavioural aspects of male homosexuality. *Medical Clinics of North America*, 1986, *70*, 537-549.*
70. Sinclair, K.C.P., & Ross, M.W. Consequences of decriminalisation of homosexuality: a study of two Australian states. *Journal of Homosexuality*, 1986, *12*, 119-127.
71. Clayer, J.R., Bookless-Pratz, C., & Ross, M.W. Evaluation of illness behaviour and exaggeration of disability. *British Journal of Psychiatry*, 1986, *148*, 296-299.
72. Ross, M.W., & Cameron, A.S. Epidemiology of AIDS retrovirus infection in South Australia. *Medical Journal of Australia*, 1986, *144*, 614-615.
73. Arrindell, W.A., Perris, C., Eisemann, M., Perris, H., van der Ende, J., Ross, M.W., Benjaminsen, S., Gaszner, P., & del Vecchio, M. Cross-national generalizability of patterns of parental rearing practices: invariance of EMBU dimensional representations of healthy subjects from Australia, Denmark, Hungary, Italy and the Netherlands. Personality and Individual Differences, 1986, 7, 103-112.
74. Ross, M.W. Illness behavior among patients attending a sexually transmitted disease clinic. Sexually Transmitted Diseases, 1987, 14, 174-179.
75. Millan, G ., & Ross, M.W. AIDS and gay youth: attitudes and lifestyle modifications in young homosexual men. Community Health Studies, 1987, 11, 50-53.
76. Ross, M.W., & Herbert, P. Responses of homosexual men to AIDS. *Medical Journal of Australia*, 1987, *146*, 280.
77. Ross, M.W. Problems with condom use in homosexual men. *American Journal of Public Health*, 1987, *77*, 877.
78. Ross, M.W., & Rosser, B.R.S. Pretest counselling for AIDS screening: a guide for the clinician. *Patient Management*, 1987, *11*(7), 93-103.
79. Ross, M.W. Ego-dystonic heterosexuality: a case study. *Journal of Homosexuality*, 1988, *15*(1&2), 7-11.*
 Italian translation as: Ross, M.W. Un caso di eterosessualità ego-distonica. *La Fenice di Babilonia*, 1996, *1*, 9-13.
80. Ross, M.W., Paulsen, J.A., & Stålström, O.W. Homosexuality and mental health: a review. *Journal of Homosexuality*, 1988, *15*(1&2), 131-152.*
81. Ross, M.W. Gay youth in four cultures: a comparitive study. *Journal of Homosexuality*, 1989, *17*, 299-314.*
82. Ross, M.W., & Rosser, B.R.S. Counselling issues in AlDS-related syndromes: a review. *Patient Education and Counselling*, 1988, *11*, 17-28.
83. Ross, M.W. Components and structure of attitudes toward AIDS. *Hospital and Community Psychiatry*, 1988, *39*, 1306-1308.
84. Ross, M.W. Effects of membership of Scientology on personality. *Journal for the Scientific Study of Religion*, 1988, *27*, 630-636.

85. Ross, M.W., & Rosser, B.R.S. Monogamy is.... *Genitourinary Medicine*, 1988, *64*, 65-66.
86. Ross, M.W., & Arrindell, W.A. Perceived parental rearing practices of homosexual and heterosexual men. *Journal of Sex Research*, 1988, *24*, 275-281.
87. Ross, M.W., & Need, J.A. Effects of adequacy of gender reassignment surgery on psychological adjustment: a follow-up of fourteen patients. *Archives of Sexual Behavior*, 1989, *18*, 145-153.
88. Ross, M.W. Prevalence of risk factors for AIDS infection in the Australian population. *Medical Journal of Australia*, 1988, *149*, 362-365.
89. Ross, M.W., Burnard, D., & Campbell, I.M. Utility of the *Gd* scale for the measurement of gender dysphoria in males. *Psychological Reports*, 1988, *63*, 87-90.
90. Ross, M.W., Carson, J.A., Cass, V.C., Hart, J., & Wilson, P. Knowledge of AIDS in Australia: a national study. *Health Education Research*, 1988, *3*, 367-373.
91. Ross, M.W. AIDS and the pursuit of happiness: some problems associated with psychosocial discrimination. Australian Journal of Social Issues, 1988, 23, 103-111.
92. Rosser, B.R.S., & Ross, M.W. Perceived emotional and life change impact of AIDS on homosexual men in two countries. *Psychology and Health*, 1988, *2*, 301-317.
93. Ross, M.W., & Rosser, B.R.S. Counselling the patient with an STD infection. *Patient Management*, 1988, *17*(8), 73-84.
94. Ross, M.W. Distribution of knowledge of AIDS: a national study. *Social Science and Medicine*, 1988, *27*, 1295-1298.
95. Ross, M.W., & Carson, J.A. Effectiveness of distribution of information on AIDS: a national study of six media in Australia. *New York State Journal of Medicine*, 1988, *88*, 239-241.
96. Ross, M.W. Attitudes toward condoms as AIDS prophylaxis in homosexual men: dimensions and measurement. *Psychology and Health*, 1988, *2*, 291-299.
97. Ross, M.W. AIDS phobias: a report of four cases. *Psychopathology*, 1988, *21*, 26-30.
98. Ross, M.W. The relationship of combinations of AIDS counselling and testing to safer sex and condom use in homosexual men. *Community Health Studies*, 1988, *12*, 322-327.
99. Ross, M.W. Prevalence of classes of risk behaviors for HIV infection in a randomly selected population. *Journal of Sex Research*, 1988, *25*, 441-450.
100. Ross, M.W. Personality factors which differentiate homosexual men with positive and negative attitudes toward condom use. *New York State Journal of Medicine*, 1988, *88*, 626-628.
101. Ross, M.W., & Seeger, V. Burnout in health professionals associated with care of patients with AIDS: predictors and dimensions. *AIDS*, 1988, *2*, 395-398.
102. Rosser, B.R.S., & Ross, M.W. A gay life events scale for homosexual men. *Journal of Gay and Lesbian Psychotherapy*, 1989, *1*(2), 87-101.
103. Ross, M.W. Psychological ethical aspects of AIDS. *Journal of Medical Ethics*, 1989, *15*, 71-84.
104. Ross, M.W., & Bell, A.C. Perceptions of AIDS: a pilot comparison with views of other selected disorders and their implications for further research. Community Health Studies, 1989, 13, 220-226.
105. Ross, M.W. Married homosexual men: prevalence and background. Marriage and Family Review, 1989, 14, 35-57.*
106. Ross, M.W., Tebble, W.E.M., & Viliunas, D. Staging of reactions to AIDS virus infection in asymptomatic homosexual men. Journal of Psychology and Human Sexuality, 1989, 2, 93-104.
107. Hafner, R.J., & Ross, M.W. The FIRO model of family therapy: implications of factor analysis. *Journal of Clinical Psychology*, 1989, *45*, 974-979.

108. Rosser, B.R.S., & Ross, M.W. Stress and anti-HIV status. *Venereology*, 1989, *2*, 91.
109. Ross, M.W., & Rosser, B.R.S. A review of education and AIDS risks, with special reference to homosexual men. In: *Living with AIDS: Report of the Third National Conference on AIDS*. Canberra: Australian Government Publishing Office, 1988.
110. Ross, M.W. Reactions to HIV positive status. In: *Living with AIDS: Report of the Third National Conference on AIDS*. Canberra: Australian Government Publishing Office, 1988.
111. Ross, M.W., Caudle, C., & Taylor, J. Social issues in AIDS prevention in adolescents: dimensions and measurement. *Journal of School Health*, 1989, *59*, 308-311.
112. Rigby, K., Anagnostou, P., Brown, M., Ross, M.W., & Rosser, B.R.S. Shock tactics to counter AIDS: the Australian experience. *Psychology and Health*, 1989, *3*, 145-159.
113. Arrindell, W.A., Ross, M.W., Bridges, K.R., van Hout, W., Hofman, A., & Sanderman, R. Fear of AIDS: are there replicable, invariant questionnaire dimensions? *Advances in Behaviour Research and Therapy*, 1989, *11*, 69-115.
114. Ross, M.W., & Rosser, B.R.S. AIDS risks and education: a review. Health Education Research, 1989, 4, 273-284.
115. Ross, M.W. Principles of patient counselling. *Viral Therapy in General Practice*,1989, *4*, 1-3.
116. Ross, M.W., Freedman, B., & Brew, R. Changes in sexual behaviour between 1986 and 1988 in matched samples of homosexually active men. *Community Health Studies*, 1989, *13*, 276-280.
117. Ross, M.W. Possible explanations of variation in admission of homosexual contact in population surveys. *AIDS*, 1989, *3*, 670.
118. Ross, M.W., & Hunter, C.E. Reactions to AIDS patients. *Hospital and Community Psychiatry*, 1989, *40*, 1077.
119. Coleman, E., Gooren, L.J.G., & Ross, M.W. Theories of gender transpositions: a critique and suggestions for further research. *Journal of Sex Research*, 1989, *26*, 525-538*.
120. Ross, M.W., & McLaws, M-L. Normative beliefs are better predictors of condom use and intention to use than behavioral beliefs. Health Education Research, 1992, 7, 335-339.
121. Ross, M.W. Reasons for non-use of condoms by homosexually active men during anal intercourse. *International Journal of STD and AIDS*, 1990, *1*, 432-434.
122. Ross, M.W. Psychological determinants of increased condom use and safer sex in homosexual men: a longitudinal study. *International Journal of STD and AIDS*, 1990, *1*, 98-101.
123. Ross, M.W., & Hafner, R.J. A comparison of the factor structure of the Crown-Crisp Experiential Index across sex and psychiatric status. *Personality and Individual Differences*, 1990, *11*, 733-739.
124. Ross, M.W. Transsexualism and transvestism. *Patient Management*, 1991, *20*, 89-94.
125. Ross, M.W. The relationship between life events and mental health in homosexual men. *Journal of Clinical Psychology*, 1990, *46*, 402-411.
126. Ross, M.W., Rigby, K., Rosser, B.R.S., Brown, M., & Anagnostou, P. The effect of a national campaign on attitudes toward AIDS. *AIDS Care*, 1990, *2*, 339-346.
127. McLaws, M-L., Oldenburg, B., Ross, M.W., & Cooper, D.A. Sexual behaviour in AIDS-related research: reliability and validity of recall and diary measures. *Journal of Sex Research*, 1990, *27*, 265-281.
128. Ross, M.W., Gold, J., Wodak, A.D., & Miller, M.E. Sexually transmissible diseases in injecting drug users. *Genitourinary Medicine*, 1991, *67*, 32-36.
129. Ross, M.W., & Rosser, B.R.S. Dimensions of sexual behaviour in homosexual men: replicability across time and country. *Psychological Reports*, 1991, *68*, 607-612.

130. Ross, M.W., & Drew, P.D. Effects of nitrite use on mitogenesis in homosexual men. *International Journal of STD and AIDS*, 1991, *2*, 133-135.
132. Ross, M.W., & Hunter, C.E. Replication of the factor structure of the Fear of AlDS Schedule across samples. Psychology and Health, 1992, 6, 39-44.
133. Kumar, B., & Ross, M.W. Homosexual behaviour and HIV infection risks in Indian males: a cross-cultural comparison. *International Journal of STD and AIDS*, 1991, *2*, 442-444.
134. Ross, M.W. Attitudes toward condoms and condom use: a review. *International Journal of STD and AIDS*, 1992, *3*, 10-16.
135. Ross, M.W., & Gregson, R.A.M. Some problems in estimating the reproductive rate of HIV infection. *Archives of AIDS Research*, 1991, *5*, 1-4.
136. Ross, M.W., Chapman, S., Wodak, A., Miller, M.E., & Gold, J. Media sources of HIV/AIDS information in injecting drug users. *Australian Journal of Public Health*, 1992, *16*, 324-327.
137. Ross, M.W. The GP's role as counsellor in HIV/AIDS. *HIV/AIDS: A Developing Issue for General Practitioners*, 1991, *4*, 10-12.
138. Rosser, B.R.S., & Ross, M.W. Psychological resistance in AIDS counselling. *Journal of Gay and Lesbian Psychotherapy*, 1991, *1*(4), 93-114.
139. Ross, M W., Caudle, C., & Taylor, J. Relationship of AIDS education and knowledge to AlDS-related social skills in adolescents. *Journal of School Health*, 1991, *61*, 351-354.
140. Wodak, A., Shaw, J.M., Gaughwin, M.D., Ross, M.W., Miller, M., & Gold, J. Behind bars: HIV risk-taking behaviour of Sydney male drug injectors while in prison. In: Norberry J, Gaughwin M, & Gerull, S.A. (eds) *HIV/AIDS and Prisons*. Canberra: Australian Institute of Criminology Conference Proceedings *4*, 1991, 239-44.
141. Ross, M .W. Factors affecting information and education, and behaviour change. *AIDS Care*, 1991, *3*, 419-421.
142. Ross, M.W., Wodak, A., Gold, J., & Miller, M.E. Differences across sexual orientation on HIV risk behaviours in injecting drug users. *AIDS Care*, 1992, *4*, 139-148.
143. Ross, M.W., & Paul, J.P. Beyond gender: the basis of sexual attraction in bisexual men and women. *Psychological Reports*, 1992, *71*, 1283-1290.*
144. Ross, M.W., Wodak, A., Miller, M.E., & Gold, J. Sexual partner choice in injecting drug users from a "critical incident" measure: its implications for estimating HIV spread. *Sexological Review*, 1993, *1*, 77-92.
145. Ross, M.W., Wodak, A., & Gold, J. Sexual behaviour in injecting drug users. *Journal of Psychology and Human Sexuality*, 1992, *5*, 89-104.
146. Kelaher, M., & Ross, M.W. Sources of bias in perception of HIV risk by injecting drug users. *Psychological Reports*, 1992, *69*,771-774.
147. Stowe, A., Ross, M.W., & Wodak, A. Contact between injecting drug users and general practitioners and its implications for health education. *Journal of the Royal Society of Health*, 1992, *112*, 122-123.
148. Stowe, A., Ross, M.W., Wodak, A., Thomas, G.V., & Larson, S.A. Significant relationships and social supports of injecting drug users and their implications for HIV/AIDS services. AIDS Care, 1993, *5*, 23-33.*
149. Ross M.W., Wodak, A., & Gold, J. Needle cleaning is associated with reduced seroprevalence among injecting drug users sharing injection equipment. *Journal of Acquired Immune Deficiency Syndromes*, 1992, *5*, 849-850.
150. Treffke H., Tiggemann, M., & Ross, M.W. The relationship between attitude, assertiveness and condom use. *Psychology and Health*, 1992, *6*, 45-52.

151. Ross, M.W., & Darke, S. Mad, bad and dangerous to know: dimensions and measurement of attitudes towards injecting drug users. *Drug and Alcohol Dependence*, 1992, *30*, 71-74.
152. Alvos, L., Gregson, R.A.M., & Ross, M.W. Future-time perspective in current and previous injecting drug users. *Drug and Alcohol Dependence*, 1993, *31*, 193-197.
153. Hunter, C.E., & Ross, M.W. Determinants of health care workers' attitudes toward people with AIDS. *Journal of Applied Social Psychology*, 1991, *21*, 947-956.
154. Bennett, L., Kelaher, M.A., & Ross, M.W. The impact of working with HIV/AIDS on health care professionals: development of the AIDS Impact Scale. *Psychology and Health*, 1994, *9*, 221-232.
155. Crofts, N., Silvester, C., & Ross, M.W. Absence of makers for HTLV infection among Australian injecting drug users. *Medical Journal of Australia*, 1992, *157*, 70-71.
156. Ross, M.W., Wodak, A., Stowe, A., & Gold, J. Explanations for sharing injection equipment in injecting drug users and barriers to safer use. *Addiction*, 1994, *89*, 473-479.
157. Ross, M.W., Buzolic, A., Wodak, A., Stowe, A., Gold, J., & Miller, M.E. Structure and measurement of dimensions of risk for HIV transmission in injecting drug users. *Drug and Alcohol Review*, 1992, *11*, 231-237.
158. Barber, J.G., Crisp, B.R., Ross, M.W., Wodak, A., Miller, M.E., & Gold, J. The social behaviour of injecting drug users. *British Journal of Social Work*, 1992, *22*, 455-462.
159. Hall, W., Darke, S., Ross, M.W., & Wodak, A. Patterns of drug use and risk-taking among injecting amphetamine and opioid users in Sydney, Australia. *Addiction*, 1993, *88*, 509-516.
160. Darke, S., Hall, W., Ross, M.W., & Wodak, A. Benzodiazepine use and HIV risk-taking behaviour among injecting drug users. *Drug and Alcohol Dependence*, 1992, *31*, 31-36.
161. Ross, M.W., Stowe, A., Loxley, W., & Wodak, A. "Home Bake" heroin use by injecting drug users. *Medical Journal of Australia*, 1992, *157*, 283-284.
162. Ross M.W., Stowe, A., Wodak A., Miller M.E., & Gold J. A comparison of drug use and HIV infection risk behaviour between injecting drug users currently-in-treatment, previously-in-treatment and never-in-treatment. *Journal of Acquired Immune Deficiency Syndromes*, 1993, *6*, 518-528.
163. Crisp B.R., Barber J.G., Ross M.W., Wodak A., Gold J., & Miller M.E. Injecting drug users and HIV/AIDS: risk behaviours and risk perception. *Drug and Alcohol Dependence*, 1993, *33*, 73-80.
164. Crisp B.R., Barber J.G., Ross M.W., Wodak A., Gold J., & Miller M.E. Predictors of unsafe injecting drug use. *Drug and Alcohol Review*, 1994, *13*, 13-19.
165 Ross, M.W. Men who have sex with men: a summary of findings from the 1992 International Conference on AIDS, Amsterdam. *AIDS Care*, 1992, *4*, 457-459.
166. Darke, S., Ross, M.W., & Hall, W. Benzodiazepine prescribing and injecting drug users. *Medical Journal of Australia*, 1992, *157*, 573-574.
167. Bell, P.F., Williams, A.K., Ross, M.W., Boswarva, P.A., & Strunin, L. HIV/AIDS knowledge and attitudes of accident and emergency health care professionals. *Journal of the Association of Nurses in AIDS Care*, 1993, *4*(4), 7-14.
168. Furner, V., & Ross, M.W. Lifestyle clues in the recognition of HIV infection: how to take a sexual history. *Medical Journal of Australia*, 1993, *158*, 40-41.*
169. Dwyer R., Richardson D., Ross M.W., Wodak A., Miller M.E., & Gold, J. Gender differences in HIV risks among injecting drug users. *AIDS Education and Prevention*, 1994, *6*, 379-389.

170. Ross, M.W., Loxley, W., Wodak, A., Buzolic, A., Monheit, B., & Stowe, A. Positive predictive value and possible explanation for self-reported false positive HIV antibody test results in injecting drug users. *American Journal of Public Health*, 1993, *83*, 1349-1350.
171. Carne, P.T., Ross, M.W., & Kemp, R.J. A practitioner's guide to HIV testing. *Medical Journal of Australia*, 1993, *158*, 267-268.*
172. Ross, M.W., Jeffords, K., & Gold, J. Reasons for entry into, and understanding of HIV/AIDS clinical trials: a preliminary study. *AIDS Care*, 1994, *6*, 77-82.
173. Akande, D., & Ross, M.W. Fears of AIDS in Nigerian students: dimensions of the Fear of AIDS Schedule (FAIDSS) in West Africa. *Social Science and Medicine*, 1994, *38*, 339-342.
174. Gold R.S., Skinner, M.J., & Ross, M.W. Unprotected intercourse in HIV-infected and non-HlV-infected gay men. *Journal of Sex Research*, 1994, *31*, 59-77.
175. Ross, M.W., Stowe, A., Wodak, A., & Gold, J. Changes in equipment sharing in injecting drug users in Sydney 1989-1990. *Drug and Alcohol Review*, 1993, *12*, 277-281.
176. Bennett, L., Kelaher, M., & Ross, M.W. Quality of life in health care professionals: burnout and its associated factors in HIV/AIDS related care. *Psychology and Health*, 1994, *9*, 273-283.
177. Ross, M.W., Kelaher, M., Wodak, A., & Gold, J. Predictors of intoxicated sex in injecting drug users. *Journal of Addictive Diseases*, 1994, *13*, 69-80.
178. Lowy, E., & Ross, M.W. "It'll never happen to me": gay men's beliefs, perceptions and folk constructions of sexual risk. *AIDS Education and Prevention*, 1994, *6*, 467-482.
179. Ross, M.W., Stowe, A., Wodak, A., Miller, M.E., & Gold, J. Predictors of HIV status among injecting drug users and health promotion. *Journal of the Royal Society of Health*, 1994, *114*, 75-80.
180. Ross, M.W., Wodak, A., & Gold, J. Accuracy of self-report of sexually transmissible disease in injecting drug users. *Journal of the European Academy of Dermatology and Venereology*, 1993, *2*, 147-148.
181. Ross, M.W. Diagnosis of transsexualism and transvestism. *General Practitioner*, 1993, *1*(15), 12-13.
182. Ross, M.W., Stowe, A., Wodak, A., Gold, J., & Miller, M.E. Reliability of interview responses of injecting drug users. *Journal of Addictive Diseases*, 1995, *14*(2), 1-12.
183. Wodak, A., Stowe, A., Ross, M.W., Gold, J., & Miller, M.E. HIV risk exposure of injecting drug users in Sydney. *Drug and Alcohol Review*, 1995, *14*, 213-222.
184. Ross, M.W., & Ryan, L. The little deaths: perceptions of HIV, sexuality and quality of life in gay men. *Journal of Psychology and Human Sexuality*, 1995, *7*, 1-20.*
185. Ross, M.W. Men who have sex with men. *AIDS Care*, 1993, *5*, 514-516.
186. Richmond, B. & Ross, M.W. Responses to AIDS-related bereavement. *Journal of Psychosocial Oncology*, 1994, *12*, 141-161.
187. Ross, M.W., Hunter, C.E., Condon, J., Collins, P., & Begley, K. The Mental Adjustment to AIDS Scale: measurement and dimensions of response to HIV/AIDS. *AIDS Care*, 1994, *6*, 407-411.
188. Kelaher, M., Ross, M.W., Rohrsheim, R., Drury, M. & Clarkson, A. Dominant situational determinants of sexual risk behaviour in gay men. *AIDS*, 1994, *8*, 101-105.
189. Tindall, B., Forde, S., Goldstein, D., Ross, M.W. & Cooper, D.A. Sexual dysfunction in advanced HIV disease. *AIDS Care*, 1994, *6*, 105-107.

190. Begley, K., Ross, M.W., Austin, P., Casey, K., Hennings, G., Agriesti, L. & Marshall, K. Development and evaluation of an inventory for rating client satisfaction with outcome in HIV counselling: the Albion Center Scale. *Patient Education and Counselling*, 1994, *24*, 341-345.
191. Tindall, B., Forde, S., Ross, M.W., Goldstein, D., Barker, S. & Cooper, D.A. Effects of two formats of informed consent on knowledge amongst persons with advanced HIV disease in a clinical trial of Didanosine. *Patient Education and Counselling*, 1994, *24*, 261-266.
192. Duque-Portugal, F., Martin, A.J., Taylor, R. & Ross, M.W. Mark-recapture estimates of injecting drug users. *Australian Journal of Public Health*, 1994, *18*, 201-204.
193. Lewis, L.A., & Ross, M.W. The gay dance party culture in Sydney: A qualitative analysis. *Journal of Homosexuality*, 1995, *29*, 41-70.
194. Caplehorn, J. & Ross, M.W. Methadone maintenance and the likelihood of risky needle-sharing. *International Journal of the Addictions*, 1995, *30*, 685-698.
195. Ross, M.W. & Rosser, B.R.S. Measurement and correlates of internalized homophobia: a factor analytic study. *Journal of Clinical Psychology*, 1996, *52*, 15-21.
196. Seibt, A.C., Ross, M.W., Freeman, A., Krepcho, M., Hedrich, A., McAlister, A. & Fernández-Esquer, M.E. Relationship between safe sex and acculturation into the gay subculture. *AIDS Care*, 1995, *7* (suppl. 1), S85-S88.
197. Ross, M.W. An adjustment to HIV scale. *Patient Education and Counseling*, 1994, *24*, 353-354. 201.
198. Muma, R.D., Ross, M.W., Parcel, G.S., and Pollard, R.B. Zidovudine adherence among individuals with HIV infection. *AIDS Care*, 1995, *7*, 439-447.
199. Des Jarlais, D.C., Hagan, H., Friedman, S.R., Friedmann, P., Goldberg, D., Frischer, M., Green, S., Tunving, K., Ljungberg, B., Wodak, A., Ross, M.W., Purchase, D., Millson, M.E., and Myers, T. Maintaining low HIV seroprevalence in populations of injecting drug users. *Journal of the American Medical Association*, 1995, *274*, 1226-1231.
200. McLaws, M-L., Irwig, L.M., Oldenburg, B., Mock, P. & Ross, M.W. Predicting intention to use condoms in homosexual men: an application and extension of the Theory of Reasoned Action. *Psychology and Health*, 1996, *11*, 745-755.
201. Nesbitt, W.H., Ross, M.W., Sunderland, R.H., and Shelp, E.E. Prediction of grief and HIV-related burnout in volunteers. *AIDS Care*, 1996, *8*, 137-143.
202. Bennett L., Ross, M.W., and Sunderland, R.H. The relationship between recognition, rewards, and burnout in AIDS caring. *AIDS Care*, 1996, *8*, 145-153.
203. Ross, M.W. How psychosocial aspects of HIV infection can affect health. *Medical Journal of Australia*, 1996, *164*, 235-237.
204. Kelly, B., Raphael, B., Statham, D., Ross, M.W., Eastwood, H., McLean, S., O'Loughlin B, and Brittain, K. A comparison of the psychosocial aspects of AIDS and cancer-related bereavement. *International Journal of Psychiatry in Medicine*, 1996, *26*, 35-49.
205. Halloran, J.P., Ross, M.W., and Huffman, L. Training persons with HIV disease for involvement in community planning process: Project LEAP. *Journal of the Association of Nurses in AIDS Care*, 1996, *7*(6), 39-47.
206. Mathai, R., Ross, M.W., and Hira, S. Concommitants of HIV/STD risk behaviors and intentions to engage in risk behaviors in adolescents in India. *AIDS Care*, 1997, *9*, 563-576.
207. Ross, M.W. Volunteers. *AIDS Care*, 1997, *9*, 95-98.
208. Lahai-Momoh, J.C and Ross, M.W. HIV/AIDS prevention-related social skills and knowledge among adolescents in Sierra Leone, West Africa. *African Journal of Reproductive Health*, 1997, *1*, 20-36.

209. Fernández-Esquer, M.A., Krepcho, M.A., Freeman, A.C., Magee, E., McAlister, A.L and Ross, M.W. Predictors of condom use among African American males at high risk for HIV. *Journal of Applied Social Psychology*, 1997, *27*, 58-74.
210. Essien, E.J., Ross, M.W., Ezedinachi, E.N.U., and Meremikwu, M. HIV/AIDS infection control enforcement: a comparison between Nigeria and the United States. *Public Health*, 1997, *111*, 205-209.
211. Essien, E.J., Ross, M.W., Ezedinachi, E.N.U., and Meremikwu, M. Cross-national HIV infection control practices and fear of AIDS: a comparison between Nigeria and the United States. *International Journal of STD and AIDS*, 1997, *8*, 764-771.
212. Leonard, L., and Ross, M.W. The last sexual encounter: the contextualization of sexual risk behavior. *International Journal of STD and AIDS*, 1997, *8*, 643-645.
213. Maruti, S., Ross, M.W., Hwang, L-Y, Leonard, L., Paffel, J., and Hollins, L. The epidemiology of early syphilis in Houston, Texas 1994-1995: gender differences in rate and presentation. *Sexually Transmitted Diseases*, 1997, *24*, 475-480.
213. Venier, J.L., Ross, M.W., and Akande, A. HIV-related anxieties in adolescents in three African countries. *Social Science and Medicine*, 1998, *46*, 313-320.
214. Ross, M.W. Race and ethnicity in STD analyses. *Sexually Transmitted Infections*, 1998, *74*, 2-3.
215. DeGuzman, M.A. and Ross, M.W. Assessing the application of AIDS-related counseling and education on the Internet. *Patient Education and Counseling*, 1999, *36*, 209-228.
216. Wong, F.Y., Chng, C.L., Ross, M.W. and Mayer, K.H. Sexualities as social roles among Asian and Pacific American Gay, lesbian, bisexual and transgender individuals: implications for community-based health education and prevention. *Journal of the Gay and Lesbian Medical Association*, 1998, *2*, 157-166.
217. Ross, M.W., Hwang L.Y., Leonard, L., Teng, M. and Duncan, L. Sexual behavior, STDs and drug use in a crack house population. *International Journal of STD and AIDS*, 1999, *10*, 224-230.
218. Kelaher, M. & Ross, M.W. Dominant situational determinants of needle sharing in injecting drug users. *Drug Education, Prevention and Policy*, 1999, *6*, 399-407.
219. Leonard, L., Chatterjee, N., Ross, M.W. Preventing syphilis: lessons from a survey of two inner-city communities in Houston, Texas. *Journal of Health Care for the Poor and Underserved*, 1999, *10*, 362-375.
220. Mathai, R., Ross, M.W., Hira, S. and McAlister, A.L. Social anxieties, social skills and sexual activities in Indian college students. *Journal of HIV/AIDS Prevention and Education for Adolescents and Children*, 1999, *3*(3), 25-41.
221. Ross, M.W., Greenfield, S.A., and Bennett, L. Predictors of Dropout and Burnout in AIDS Volunteers: A Longitudinal Study. *AIDS Care*, 1999, *11*, 723-731.
222. Baseman, J., Ross, M.W., and Williams, M. Sale of sex for drugs and drugs for sex: an economic context of sexual risk behavior for STDs. *Sexually Transmitted Diseases*, 1999, *26*, 444-449.
223. Chatterjee, N., Leonard, L., and Ross, M.W. The role of private physicians in STD control in two inner-city communities in Houston, Texas. *Journal of Public Health Management and Practice*, 1999, *5*(5), 34-39.
224. Hospers, H.J., Debets, W., Ross, M.W., and Kok, G. Evaluation of an HIV prevention intervention for men who have sex with men at cruising areas in the Netherlands. *AIDS and Behavior*, 1999, *3*, 359-366.
225. Ross, M.W., and Ferreira-Pinto, J.B. Toward a public health of situations: The re-contextualization of risk. *Cadernos de Saúde Pública*, *16*(1), 2000, 59-71.
226. Essien, E.J., Ross, M.W., Ezedinachi, E.N.U., and Meremikwu, M. Measuring AIDS Fears in Health Workers: Structure of the FAIDSS across Countries. *International Journal of Intercultural Relations*, 2000, *24*, 125-129.

227. Williams, M., Bowen, A., Ross, M.W., Freeman, R., and Elwood, W.N. Perceived compliance with AZT dosing among a sample of African-American drug users. *International Journal of STD and AIDS*, 2000, *11*, 57-63.
228. Lee, D.H., Ross, M.W., Mizwa, M., and Scott, D.P. HIV risks in a homeless population. *International Journal of STD and AIDS*, 2000, *11*, 509-515.
229. Yaganehdoust, A., Graviss, E.A., Ross, M.W., Adams, G.J., Ramaswamy, S., Wanger, A., Frothingham, R., Soini, H., and Musser, J.M. Complex transmission dynamics of clonally related virulent *Mycobacterium tuberculosis* associated with barhopping by predominantly Human Immunodeficiency Virus-positive gay men. *Journal of Infectious Diseases*, 1999, *180*, 1245-1251.
230. Ross, M.W., Tikkanen, R. and Månsson, S.A. Differences between Internet samples and conventional samples of men who have sex with men: Implications for research and HIV interventions. *Social Science and Medicine*, 2000, *51*, 749-758.
231. Tikkanen, R. and Ross, M.W. Looking for sexual compatibility: experiences among Swedish men visiting Internet gay chat rooms. *CyberPsychology and Behavior*, 2000, *3*, 605-616.
232. Donovan, B. and Ross, M.W. HIV risk evaluation. *Australian Family Physician*, 2000, *29*, 646-650.
233. Brimlow, D.L., **Ross**, M.W., and Rankin, K.V. The perception of surrogate teaching patients with HIV disease of dental providers' fear and comfort. *Journal of Dental Education*, 2000, 597-602.
234. Donovan, B. and **Ross**, M.W. Preventing HIV: Determinants of sexual behavior. *The Lancet*, 2000, *355*, 1897-1901.
235. Hwang, L-Y., **Ross**, M.W., Zack, C., Bull, L., Rickman, C., and Holleman, M. Prevalence of sexually transmitted infections and associated risk factors in drug abuse populations. *Clinical Infectious Diseases*, 2000, *31*, 920-926.
236. Essien, E.J., **Ross**, M.W., Linares, A.C., and Osemene, N.I. Perception of reliability of HIV/AIDS information sources: A comparison among whites, African Americans and Hispanics in Houston, Texas. *Journal of the National Medical Association*, 2000, *92*, 269-274.
237. Peters, R.J., Kelder, S., **Ross**, M.W., McAlister, A., Meshack, A., Lewis, S., Baumler, E. and Shinn, E. Project Alpha: A culturally appropriate approach to adolescent male sex education. *Venereology*, 2000, 13, 57-62.
238. **Ross, M.W**. and Wells, A.L. The modernist fallacy in homosexual selection theories: Homosexual and homosocial exaptation in South Asian society. *Psychology, Evolution and Gender*, 2000, *2*, 253-262.
239. Fernández-Esquer, M.E., **Ross**, M.W. and Torres, I. The importance of psychosocial factors in the prevention of HPV infection and cervical cancer. *International Journal of STD and AIDS*, 2000, *11*, 701-713.
240. **Ross**, M.W. Le psychologue au tribunal: La théorie et l'expérience. *Pratiques Psychologiques*, 2000, *4*, 83-89.
241. Rosser, B.R.S., Rugg, D.L. and **Ross**, M.W. Increasing research and evaluation productivity: Tips for successful writing retreats. *Health Promotion Practice*, 2001, 2, 9-13.
242. Klovdahl, A.S., Graviss, E.A., Yaganehdoost, A., **Ross, M.W.**, Wanger, A., Adams, G.J. and Musser, J.M. Networks and tuberculosis: An undetected community outbreak involving public places. *Social Science and Medicine*, 2001, *52*, 681-694.
243. Mattison, A.M., **Ross, M.W.**, Wolfson, T., Franklin, D. and the HNRC Group. Circuit party attendance, club drug use and unsafe sex in gay men. *Journal of Substance Abuse*, 2001, *13*, 119-126.
244. Koumans, E.H., Farley, T.A., Gibson, J.J., Langley, C., **Ross, M.W.**, McFarlane, M., Braxton, J., and St Louis, M.E. Characteristics of persons with syphilis in areas of persisting syphilis in the U.S.: Sustained transmission associated with concurrent partnerships. *Sexually Transmitted Diseases*, 2001, *28*, 497-503.

245. Timpson S.C., Pollack, K.I., Bowen, A.M., Williams, M.L., **Ross, M.W.**, McCoy, C.B. and McCoy, H.V. Gender differences in the process of change for condom use: Patterns across stages of change in crack cocaine users. *Health Education Research*, 2001, *16*, 541-553.
246. Timpson, S.C., Pollack, K.I., Williams, M.L., **Ross, M.W.**, Kapadia, A.S., Bowen, A.M., McCoy, C.B., and McCoy, H.V. Predictors of stages of change for condom use in crack cocaine users. *AIDS and Behavior*, 2001, *5*, 65-74.
247. Mâsse, L.C. and **Ross, M.W**. Assessing differential item validity of the AIDS-related social skills questionnaire among African adolescents. *Social Science Research*, 2001, *30*, 50-61.
248. Johnson, R.J., Baumler, E.R., Yacoubian, G.S., Peters, R.S., and **Ross, M.W.** A longitudinal analysis of drug use reporting among Houston arrestees. *Journal of Drug Issues*, 2001, *31*, 757-766.
249. Selvan, M.S., **Ross, M.W.**, Kapadia, A.S., Mathai, R. and Hira, S. Study of perceived norms, beliefs and intended sexual behavior among higher secondary school students in India. *AIDS Care*, 2001, *13*, 779-788.
250. Reininger, B., Martin, D.W., **Ross, M.W.**, Sinicrope, P.S., and Dinh-Zarr, T. Advancing the theory and measurement of collective empowerment: A qualitative study. *International Quarterly of Community Health Education*, 2000, *19*, 293-320.
251. Williams, M.L., **Ross, M.W.**, Bowen, A.M., Timpson, S., McCoy, H.V., Perkins, K., Saunders, L., and Young, P. An investigation of condom use by frequency of sex. *Sexually Transmitted Infections*, 2001, *77*, 433-435.
252. **Ross, M.W.** and Williams, M.L. Sexual behavior and illicit drug use. *Annual Review of Sex Research*, 2001, *12*, 290-310.
253. Reynolds, S.L., Kapadia, A.S., Leonard, L., and **Ross, M.W.** Examining the direct costs and effectiveness of syphilis detection by selective screening and partner notification. *Journal of Public Health Medicine*, 2001, *23*, 339-345.
254. Baseman, J., Leonard, L., **Ross, M.W.**, and Hwang, L-Y. Acceptance of syphilis screening among residents of high STD-risk Houston communities. *International Journal of STDs and AIDS*, 2001, *12*, 744-749.
255. **Ross, M.W.**, Rosser, B.R.S., Bauer, G.R., Bockting, W.O., Robinson, B.E., Rugg, D.L., and Coleman, E. Drug use, unsafe sexual behavior, and internalized homonegativity in men who have sex with men. *AIDS and Behavior*, 2001, *5*, 97-103.
256. Essien, E.J., **Ross, M.W**., and Meshack, A.F. Misperceptions about HIV transmission and reliability of information sources in African American and Mexican American men and women. *Journal of the National Medical Association*, 2002, *94*, 304-312.
257. Wiltshire, A.D., **Ross, M.W.**, and Brimlow, D.L. Empathic communication between dental professionals and persons living with HIV and AIDS. *Journal of Dental Education*, 2002, *66*, 86-93.
258. Ezedinachi, N.E.U., **Ross, M.W**., Meremiku, M., Essien, E.J., Edem, C., Ekure, E., and Ita, O. The impact of an intervention to change health workers' HIV/AIDS attitudes and knowledge in Nigeria: A controlled trial. *Public Health*, 2002, *116*, 106-112.
259. Rosser, B.R.S., Bockting, W., Rugg, D., Robinson, B., Ross, M.W., Bauer, G., Kraft, C. and Coleman, E. A randomized controlled intervention trial of a sexual health approach to long-term HIV risk reduction for men who have sex with men. Effects of the intervention on unsafe sex behavior. *AIDS Education and Prevention*, 2002, *14* Suppl. A, 59-71.
260. **Ross, M.W.** and Williams, M.L. Effective targeted and community STD/HIV prevention programs. *Journal of Sex Research*, 2002, *39*, 58-62.
261. Buzi, R.S., Tortolero, S.R., Smith, P., and **Ross, M.W.** Young females' perception of sexual abuse:A focus group approach. *North American Journal of Psychology*, 2002, *4*, 441-456.
262. **Ross, M.W.** Sexuality and health challenges: Responding to a public health imperative. *Journal of Sex Research*, 2002, *39*, 7-9.
263. **Ross, M.W.**, Hwang, L-Y., Zack, C., Bull, L. and Williams, M.L. Sexual risk behaviors and STIs in drug abuse treatment populations whose drug of choice is crack cocaine. *International Journal of STD and AIDS*, 2002, *13*, 769-774.

264. Peters, R., Yacoubian, G., Baumler, E., **Ross, M.W.**, and Johnson, R. Heroin Use among Southern Arrestees: Regional Findings from the Arrestee Drug Abuse Monitoring (ADAM**)** Program. *Journal of Addictions and Offender Therapy*, 2002, *22* (2), 50-60.
265. Wang, Q. and **Ross, M.W.** Differences between chat room and e-mail sampling approaches in Chinese men who have sex with men. *AIDS Education and Prevention*, 2002, *14*, 361-366.
266. **Ross, M.W.**, Essien, E.J., Williams, M.L. and Fernández-Esquer, M.E. Concordance between sexual behavior and sexual identity in street outreach samples of four racial/ethnic groups. *Sexually Transmitted Diseases*, 2003, *30*, 110-113.
267. **Ross, M.W.**, Mattison, A.M. and Franklin, D.R. Club drugs and sex on drugs are associated with different motivations for circuit party attendance in gay men. *Substance Abuse and Misuse*, 2003, *38*, 1173-1183.
268. **Ross, M.W.**, Timpson, S.C., Williams, M.L. and Bowen, A.M. Situational correlates of condom use in a sample of African American drug users who are primarily crack cocaine users. *AIDS and Behavior*, 2003, *7*, 55-60.
269. Tikkanen, R. and **Ross, M.W.** Technological tearoom trade: Characteristics of Swedish men visiting gay internet chat rooms. *AIDS Education and Prevention*, 2003, *15*, 122-132.
270. Buzi, R.S., Tortolero, S.R., Roberts, R.E., **Ross, M.W.**, Markham, C.M., and Fleschler, M. Gender differences in the consequences of a coercive sexual experience among adolescents attending alternative schools. *Journal of School Health*, 2003, *73*, 191-196.
271. Williams, M.L., Timpson, S., Klovdahl, A., Bowen, A.M., **Ross, M.W.**, and Keel, K.B. HIV risk among a sample of drug using male sex workers. *AIDS*, 2003, *17*, 1402-1404.
272. Beatty, R.L., Gruskin, E., Hsi, A., Jillson, I.A., Neisen, J., and **Ross, M.W.** Bridging science and practice in LGBT health. *Clinical Research and Regulatory Affairs*, 2003, *20*, 229-246.
273. **Ross, M.W.**, Daneback, K., Månsson, S-A., Cooper, A., and Tikkanen, R. Characteristics of men and women who complete and exit from an on-line internet sexuality questionnaire. *Journal of Sex Research*, 2003, *40*, 396-402.
274. Rosenthal, L., Scott D.P., Kelleta, Z., Zikarge, A., Momoh, M., Momoh-Lahai, J., **Ross, M.W**., and Baker, A. Assessing the HIV/AIDS health service needs of African immigrants to Houston. *AIDS Education and Prevention*, 2003, *15*, 570-580.
275. **Ross, M.W.**, and Brooks, Ann K. Sexual behavior and the non-construction of sexual identity: Implications for the analysis of men who have sex with men and women who have sex with women. *Radical Statistics*, 2003, *83*, 2-15.
276. Crisp, B.R. and **Ross, M.W.** Borders of evidence: A critical reflection. *Radical Statistics*, 2003, *83*, 71-86.
277. Cooper, A., Månsson, S-A., Daneback, K., Tikkanen, R. and **Ross, M.W.** Predicting the future of Internet sex: Online sexual activities in Sweden. *Sexual and Relationship Therapy*, 2003, *18*, 277-291.
278. **Ross, M.W.**, Henry, D., Freeman, A., Caughy, M. and Dawson, A.G. Environmental influences on safer sex in young gay men: A situational presentation approach to measuring influences on sexual health. *Archives of Sexual Behavior*, 2004, *33*, 249-257.
279. **Ross, M.W.**, Chatterjee, N., and Leonard, L. A community-level syphilis prevention program: Outcome data from a controlled trial. *Sexually Transmitted Infections*, 2004, *80*, 100-104.
280. Scott, D.P., Harzke, A.J., Mizwa, M.B., Pugh, M., and **Ross, M.W.** Evaluation of an HIV peer education program in Texas prisons. *Journal of Correctional Health*, 2004, *10*, 151-173.
281. Harzke, A.J., Williams, M.L., Nilsson Schönnesson, L., **Ross, M.W.**, and Timpson, S.C. Factors associated with adherence with antiretroviral medications in a sample of HIV-positive African American drug users. *AIDS Care*, 2004, *16*, 458-470.
282. Crisp, B., Williams, M, Timpson, S, and **Ross, M.W.** Medication compliance and satisfaction with treatment for HIV disease in a sample of African American crack cocaine smokers. *AIDS and Behavior*, 2004, *8*, 1999-206.
283. Des Jarlais, D.C., Lyles, C., Crepaz, N. and the TREND (Transparent Reporting of Evaluations with Non-randomized Designs) group. Improving the reporting quality of nonrandomized

evaluations of behavioral and public health interventions: The TREND statement. *American Journal of Public Health*, 2004, *94*, 361-366.

284. Cochran, S.D., Ackerman, D., Mays, V.M., and **Ross, M.W.** Prevalence of non-medical drug use and dependency among homosexually active men and women in the U.S. population. *Addictions*, 2004, *89*, 989-998.
285. Nilsson Schönnesson, L. **Ross, M.W.**, and Williams, M.L. The HIV Medication Self-reported Non-adherence reasons (SNAR) Index and its underlying psychological dimensions. *AIDS and Behavior*, 2004, *8*, 293-301.
286. Buzi, R.S., Tortolero, S.R., Roberts, R.E., **Ross, M.W.**, Addy, R.C. & Markham, C.M. The impact of a history of sexual abuse on high-risk sexual behaviors among females attending alternative schools. *Adolescence*, 2003, *38*, 595-606.
287. **Ross, M.W.**, Elford, J., Sherr, L., and Hart, G. The TREND statement: social science, communication, and HIV/AIDS. *AIDS Care*, 2004, *16*, 667-668.
288. **Ross, M.W.**, Courtney, P., Dennison, J. and Risser, J. Incomplete reporting of race and ethnicity in gonorrhea cases and potential bias in disease reporting by private and public sector providers. *International Journal of STD and AIDS*, 2004, *15*, 778.
289. Wang, Q., Lin, G., and **Ross, M.W.** Sexual risk behaviors among men who have sex with men: An Internet outreach sample. *Chinese Journal of Prevention and Control of STDs and AIDS*, 2004, *10*, 335-337.
290. Essien, E.J., Meshack, A., **Ross, M.W.**, Williams, M.L., Fernández-Esquer, M.E., Peters, R.O., and Ogungbade, G.O. Primary source of income is associated with differences in HIV risk behaviors in street-recruited samples. *International Journal for Equity in Health*, 2004, *3*(1), article 5.
291. McFarlane, M., **Ross, M.W**., and Elford, J. [Editorial]. The Internet and HIV/STD prevention. *AIDS Care*, 2004, *16*, 929-930.
292. **Ross, M.W.**, Rosser, S., and Stanton, J. Beliefs about cybersex and Internet-mediated sex of Latino men who have Internet sex with men: relationships with sexual practices in cybersex and in real life. *AIDS Care*, 2004, *16*, 1002-1011.
293. **Ross, M.W**., Rosser, B.R.S., Stanton, J., and Konstan J. Characteristics of Latino men who have sex with men on the Internet who complete and drop out of an Internet-based sexual behavior survey. *AIDS Education and Prevention*, 2004, *16*, 526-537.
294. Dawson, A.G., **Ross, M.W.**, Henry, D., and Freeman, A. Evidence of risk in “barebacking” men who have sex with men: Cases from the Internet. *Journal of Gay and Lesbian Psychotherapy*, 2005, *9*, 73-83*.
295. Essien, E.J., **Ross, M.W.**, Fernández-Esquer, M.E., and Williams, M.L. Reported condom use and condom use difficulties in street outreach samples of men of four racial and ethnic backgrounds. *International Journal of STDs and AIDS*, 2005, *16*, 739-743.
296. Konstan, J.A., Rosser, B.R.S., **Ross, M.W.**, Stanton, J., and Edwards, W.M. The story of Subject Naught: A cautionary but optimistic tale of internet survey research. *Journal of Computer-Mediated Communication*, 2005, 10(2), article 11, DOI: 10.1111/j.1083-6101.2005.tb00248.x
297. Johnson, R.J., **Ross, M. W.**, Taylor, W. C., Williams, M. L., Carjaval, R. I., and Peters, R. J. A history of drug use and childhood sexual abuse among incarcerated males in a county jail. *Substance Use and Misuse*, 2005, *40*, 211-229.
298. **Ross, M.W.**, Månsson, S-A., Daneback, K., Cooper, A. and Tikkanen, R. Biases in Internet sexual health samples: Comparison of an Internet sexuality survey and a national sexual health survey in Sweden. *Social Science and Medicine*, 2005, *61*, 245-252.
299. **Ross, M.W.**, Månsson, S-A., Daneback, K., and Tikkanen, R. Characteristics of men who have sex with men on the Internet but identify as heterosexual, compared with heterosexually identified men who have sex with women. *Cyberpsychology and Behavior*, 2005, *8*, 131-139.
300. Williams, M.L., Atkinson, J., Klovdahl, A., **Ross, M.W.**, and Timpson, S. Spatial bridging in a network of drug-using male sex workers. *Journal of Urban Health*, 2005, *82 (1 suppl. 1)*, i35-i42.

301. **Ross, M.W**., and Fernández-Esquer, M.E. Ethnicity in sexually transmitted infections and sexual behavior research. *The Lancet*, 2005, *365*, 1209-1210.
302. Hamra, M., **Ross, M.W.**, Karuri, K.K., Orrs, M., and D'Agostino, A. The relationship between expressed HIV/AIDS-related stigma and beliefs and knowledge about care and support of people living with AIDS in families caring for HIV infected children in Kenya. *AIDS Care,* 2005, *17*, 911-922.
303. **Ross, M.W**. Typing, doing and being: Sexuality and the Internet. *Journal of Sex Research*, 2005, *42*, 342-352.
304. Carvajal, R.I., **Ross, M.W**., Byrd, T., Shelton, A., and Johnson, R.J. Analysis of HIV data collected in the Harris County Jail, Texas 1992-2000: Counseling and testing program female inmates. *Journal of Correctional Health*, 2005, *11*, 271-287.
305. McCurdy, S.A., Williams, M.L., Kilonzo, G.P., Ross, M.W., and Leshabari, M.T. Heroin and HIV risk in Dar es Salaam, Tanzania: Youth hangouts, *mageto* and injecting practices. *AIDS Care*, 2005, *17*, **(Supplement 1): S65-76.**
306. Johnson, J., Timpson, S., Williams, M.L., **Ross, M.W.**, and Atkinson J. A comparison of two practical approaches for imputing interim analysis and stochastic curtailment in a field trial. *Clinical Trials*, 2005, *2*, Suppl 1, S23-S92, S88, 97.
307. Timpson, S.C., Williams, M.L., **Ross, M.W.**, and Keel, K.B. African American crack users' attitudes and beliefs about male and female condoms. *Journal of Substance Abuse*, 2005, *10*, 207-213.
308. McCurdy S.A., Williams, M.L., **Ross, M.W.**, Kilonzo, G.P. and Leshabari M.T. New injecting practice increases HIV risk among drug users in Tanzania [letter]. *British Medical Journal*, 2005, *331*, 778.
309. Nilsson Schönnesson, L., **Ross, M.W.**, and Bergbrant, I-M. Coping modes with HIV disease predict loss from HIV study cohort. *International Journal of STD and AIDS*, 2005, *16*, 479-481.
310. Khalil, S.N., Ross, M.W., Mathai, R. and Hira, S. Knowledge and attitudes toward HIV/STD among Indian adolescents. International Journal of Adolescence and Youth, 2005, 12, 149-168, **DOI:**10.1080/02673843.2005.9747948
311. Essien, E.J., Meshack, A.F., Ekong, E., Williams, M.L., Amos, C.E., James, T.M., Peters, R.J., Ogungbade, G., and **Ross, M.W.** Effectiveness of a situationally-based HIV risk-reduction intervention for the Nigerian Uniformed Services on readiness to adopt condom use with casual partners. *Counselling, Psychotherapy and Health*, 2005, *1*, 19-30.
312. Selvan, M.S., **Ross, M.W.**, and Parker, P. Societal norms and open communication about sex-related issues as predictors of safer sex. *Indian Journal of Community Medicine*, 2005, *30*(4), 10-12.
313. Selvan, M.S., **Ross, M.W.**, Nagaraj, S., Etzel, C.J., and Shete, S. Perception among upper middle class adolescent in Bombay regarding sex and sexuality. *Indian Journal of Public Health*, 2005, *49*, 250-251.
314. **Ross, M.W.**, Essien, E.J., Ekong, E., James, T.M., Amos, C., Ogungbade, G.O., and Williams, M.L. The impact of a situationally-focused individual HIV/STD risk reduction intervention on risk behavior in a one-year cohort of Nigerian military personnel. *Military Medicine*, 2006, *17*, 970-975.
315. Johnson, R.J., **Ross, M.W.**, Taylor, W.C., Williams, M.L., Carvajal, R.I., and Peters, R.J. Prevalence of childhood sexual abuse among incarcerated males in county jail. *Child Abuse and Neglect*, 2006, *30*, 75-80.
316. Harzke, A.J., **Ross**, M.W., and Scott, D.P. Predictors of post release healthcare utilization among HIV positive inmates: A pilot study. *AIDS Care*, 2006, *18*, 290-301.
317. Nilsson Schönnesson L., Diamond, P.M., **Ross**, M.W., Williams, M.L., and Bratt, G. Baseline predictors of three types of antiretroviral therapy (ART) adherence: A 2-year follow-up. *AIDS Care*, 2006, *18*, 406-414.

318. Williams M., **Ross M.W.**, Atkinson J., Bowen A., Klovdahl A., and Timpson S.C. An investigation of concurrent sex partnering in two samples having large numbers of sex partners. *International Journal of STD and AIDS*, 2006, *17*, 309-314.
319. **Ross, M.W.**, Risser, J., Peters, R.J., and Johnson, R.J. Cocaine use and syphilis trends: Findings from the Arrestee Drug Abuse Monitoring (ADAM) program and syphilis epidemiology in Houston. *American Journal on Addictions*, 2006, *15*, 473-477.
320. Springer, A., Parcel, G., Baumler, E., and **Ross, M.W.** Supportive social relationships and adolescent health risk behavior among secondary school students in El Salvador. *Social Science and Medicine*, 2006, *62*, 1628-1640.
321. Hamra, M., **Ross, M.W.**, Orrs, M., and D'Agostino, A. Relationship between expressed HIV/AIDS-related stigma and HIV beliefs-knowledge and behaviour in families of HIV infected children in Kenya. *Tropical Medicine and International Health*, 2006, *11*, 513-527.
322. Daneback, K., **Ross, M.W.**, and Månsson, S-A. Characteristics and behaviors of sexual compulsives who use the Internet for sexual purposes. *Sexual Addiction and Compulsivity*, 2006, *13*, 53-67.
323. McCurdy, S.A., **Ross, M.W.**, Kilonzo, G.P., Leshabari, M.T., and Williams, M.L. HIV/AIDS and injection drug use in the neighborhoods of Dar es Salaam, Tanzania. *Drug and Alcohol Dependence*, 2006, *82 (Suppl. 1)*, S23-S27.
324. Williams, M.L., Bowen, A.M., Timpson, S.C., **Ross, M.W.**, and Atkinson, J.S. HIV prevention and street-based male sex workers: An evaluation of brief interventions. *AIDS Education and Prevention*, 2006, *18*, 204-215.
325. **Ross, M.W.**, Rosser, B.R.S., Coleman, E. and Mazin, R. Misrepresentation on the Internet and in real life about sex and HIV: A study of Latino men who have sex with men. *Culture, Health and Sexuality*, 2006, *8*, 133-144.
326. Selvan, M.S., **Ross, M.W.**, Nagaraj, S., and Kaila, H.L. Attitude and knowledge about school-based intervention and prevention programs among adolescent students. *Health Administrator*, 2006, *17*, 42-48.
327. **Ross, M.W.**, Essien, E.J., and Torres, I. Conspiracy beliefs about the origin of HIV/AIDS in four racial/ethnic groups. *Journal of Acquired Immune Deficiency Syndromes*, 2006, *41*, 342-344.
328. Bowen A., Williams, M.L., Dearing, E., Timpson, S.C., and **Ross, M.W**. Male heterosexual crack smokers with multiple sex partners: Between and within person predictors of condom intention. *Health Education Research*, 2006, *21*, 459-559.
329. Selane, P., Kamiru, H.N., and **Ross, M.W**. Dimensions of the Fear of AIDS scale among South African students. *Counselling, Psychotherapy and Health*, 2006, *2*(2), 1-14.
330. Crisp, B.R., Williams, M.L., **Ross, M.W**. and Timpson, S.C. The impact of religious affiliation while growing up on substance use by African American cocaine users. *Social Work and Christianity*, 2006, 33, 90-100.
331. Crisp, B.R., Williams, M.L., Ross, M.W., and Timpson, S.C. Correlates of sexual assault in a sample of male African American crack cocaine users. *Health Sociology Review*, 2006, *15*, 258-268.
332. **Ross, M.W**., Harzke, A.J., Scott, D.P., McCann, K., and Kelley, M. Outcomes of Project Wall Talk: An HIV/AIDS peer education program implemented within the Texas state prison system. *AIDS Education and Prevention*, 2006, *18*, 504-517.
333. **Ross, M.W**. A commentary on publication of "The gay cruise: Developing a theory- and evidence-based Internet HIV-prevention intervention": Bringing the new generation of Internet-based prevention into the research world [editorial]. *Sexuality Research and Social Policy*, 2006, *3*(2), 68-69.
334. Williams, M.L., Bowen, A.M., Timpson, S.C., Ross, M.W., and Atkinson, J.S. HIV prevention and street-based male sex workers: An evaluation of brief interventions. *AIDS Education and Prevention*, 2006, *18*, 204-215.

335. Risser, J.M.H., Timpson, S.C., McCurdy, S.A., Ross, M.W., and Williams, M.L. Psychological correlates of trading sex for money among African American crack cocaine smokers. *American Journal of Drug and Alcohol Abuse*, 2006, *32*, 645-653.
336. Whitby, M., McLaws, M-L., and Ross, M.W. Why healthcare workers don’t wash their hands: A behavioral explanation. *Infection Control and Hospital Epidemiology*, 2006, *27*, 484-492.
337. Ross, M.W., Rosser, B.R.S., McCurdy, S.A., and Feldman, J. The advantages and limitations of seeking sex online: A comparison of reasons given for online and offline sexual liaisons by men who have sex with men. *Journal of Sex Research*, 2007, *44*, 59-71.
338. Ross, M.W., Timpson, S.C., Williams, M.L., Charles Amos, C., McCurdy, S., Bowen, A.M., and G.P. Kilonzo, G.P. Responsibility as a dimension of HIV prevention normative beliefs: Measurement in three drug-using samples. *AIDS Care*, 2007, *19*, 403-409.
339. Nilsson Schönnesson, L., Williams, M.L., Ross, M.W., Bratt, G., and Keel, B. Factors associated with suboptimal antiretroviral therapy adherence to dose, schedule, and dietary instructions. *AIDS and Behavior*, 2007, *11*, 175-183.
340. Brennan, D.J., Hellerstedt, W.L., Ross, M.W., and Welles, S.L. History of childhood sexual abuse and HIV risk behaviors in gay and bisexual men. *American Journal of Public Health*, 2007, *97*, 1107-1112.
341. Shaikh, F.D., Shaikh, S.A., **Ross, M.W.**, and Grimes, R. Knowledge and attitudes of Pakistani medical students towards HIV-positive and/or AIDS patients. *Psychology, Health and Medicine*, 2007, *12*, 7-17.
342. Peters, R.J., Williams, M.L., **Ross, M.W.**, Atkinson, J., and Yacoubian, G.S. Codeine cough syrup use among African American crack cocaine smokers. *Journal of Psychoactive Drugs*, 2007, *39*, 97-102.
343. Miner, M.H., Coleman, E., Center, B.A., Ross, M.W., and Rosser, B.R.S. The Compulsive Sexual Behavior Inventory: Psychometric properties. Archives of Sexual Behavior, 2007, 36, 579-587.
344. Pequegnat, W., Rosser, B.R.S., Bowen, A.M., Bull, S.S., DiClemente, R.J., Bockting, W.O., Elford, J., Fishbein, M., Gurak, L., Horvath, K., Konstan, J., Noar, S.M., Ross, M.W., Sherr, L., Speigel, D., and Zimmerman, R. Conducting Internet-based HIV/STD prevention research: Considerations in design and evaluation. AIDS and Behavior, 2007, 11, 505-521.
345. Nilsson Schönnesson, L., Williams, M.L., Ross, M.W., Diamond, P.M., and Keel, B. Three types of adherence to HIV antiretroviral therapy and their associations with AIDS diagnosis, medication side effects, beliefs about antiretroviral therapy, and belief about HIV disease. International Journal of STD and AIDS, 2007, 18, 369-373.
346. Ross, M.W., Timpson, S.C., Williams, M.L., and Bowen, A. The Impact of HIV-related interventions on HIV risk behavior in a community sample of African American crack cocaine users. *AIDS Care*, 2007, 19, 608-616.
347. Ross, M.W., Timpson, S.C., Williams, M.L., Amos, C. and Bowen, A. Stigma consciousness concerns related to drug use and sexuality in a sample of street-based male sex workers. International Journal of Sexual Health, 2007, 19, 57-67.
348. Timpson, S.C., Ross, M.W., Williams, M.L., and Atkinson, J. Characteristics, drug use, and sex partners of a sample of male sex workers. The American Journal of Drug and Alcohol Abuse, 2007, 33, 63-69.
349. Ngo, D.A., Ratliff, E.A., McCurdy, S.A., Ross, M.W., Markham, C., and Pham, H.T.B. Health Seeking Behaviour for Sexually Transmitted Infections and HIV Testing Among Female Sex Workers in Vietnam. AIDS Care, 2007, 19, 878-887.
350. Adu-Oppong, A., Grimes, R.M., Ross, M.W., Risser, J., and Kessie, G. Social and behavioral determinants of consistent condom use among female commercial sex workers in Ghana. AIDS Education and Prevention, 2007, 19, 160-172.

351. Ross, M.W. Commentary. Sexually Transmitted Infections, 2007, 83, 205.
352. Daneback, K., Månsson, S-A., and Ross, M.W.Using the internet to find offline sex partners. CyberPsychology & Behavior, 2007, 10, 100-107.
353. Ross, M.W. Relationships between homosexual and heterosexual interest and their implications for bisexuality: An empirical test. Journal of LGBT Health Research, 2007, 3, 21-23.
354. Ross, M.W., Berman, S.M., Aral, S.O., Courtney, P.E., Dennison, J.M., Klovdahl, A.S., Williams, M.L. and St Lawrence, J.S. Process, efficacy and sample demographics of three approaches to behavioral surveillance for gonorrhea: Case interviews, place surveys, and network studies. International Journal of STD and AIDS, 2007, 18, 846-850.
355. Ross, M.W. Situating sexuality electronically: The Internet and sexual expression. Sexuality Research and Social Policy, 2007, 4(2), 1-4.
356. Thomas, A.B., Ross, M.W., and Harris, K.K. Coming out online: Interpretations of young men's stories. Sexuality Research and Social Policy, 2007, 4(2), 5-17.
357. Ngo, D.A., McCurdy, S.A., Ross, M.W., Markham, C., Ratliff, E.A., and Pham, H.T.B. The lives of female sex workers in Vietnam: Findings from a qualitative study. Culture, Health and Sexuality, 2007, 9, 555-570.
358. Ferris, M., Burau, K., Constantin, A.M., Mihale, S., Murray, N., Preda, A., Ross, M.W., and Kline, M.W. Influence of institutionalization on time to HIV disease progression in a cohort of Romanian children and teens. *Pediatrics, 2007, 120, 476-80.*
359. Ferris, M., Burau, K., Ana Maria Constantin, A.M., Dunn, K., Mihale, S., Murray, N., Preda, A., Ross, M.W., and Kline, M. The influence of disclosure of HIV diagnosis on time to disease progression in a cohort of Romanian children and teens. AIDS Care, 2007, 19, 1088-94.
360. Essien, E.J., Ogungbade, G.O., Ward, D., Ekong, E., Ross, M.W., Meshack, A., and Holmes, L. Influence of educational status and other variables on HIV risk perception among military personnel: A large cohort finding. Military Medicine, 2007, 172, 1177-1181.
361. Ross, M.W., Daneback, K., Månsson, S-A., Berglund, T. and Tikkanen, R. Reported sexually transmitted infections in Swedish Internet-using men and women. Journal of the European Academy of Dermatology and Venereology, 2008, 22, 696-703.
362. Pallonen, U.E., Williams, M.L., Timpson, S.C., Bowen, A., and **Ross, M.W**. Personal and partner measures in stages of consistent condom use among African American heterosexual crack cocaine smokers. AIDS Care, 2008, 20, 212-220.
363. Davies, D.A., Welles, S.L., Hellerstedt, W.L., and **Ross, M.W**. Body image, body satisfaction, and unsafe anal intercourse among men who have sex with men. Journal of Sex Research, 2008, 45, 49-56.
364. Umeh, C.N., Essien, E.J., Ezedinachi, E.N., and **Ross, M.W**. Knowledge, beliefs and attitudes about HIV/AIDS-related issues, and sources of knowledge among health care professionals in Southern Nigeria. Journal of the Royal Society of Health, 2008, 128, 233-239.
365. Jain, A. and **Ross, M.W**. Predictors of dropout in an Internet study of men who have sex with men. Cyberpsychology and Behavior, 2008, 11, 583-6.
366. Rosser, B.R.S., Bockting, W.O., **Ross, M.W**., Miner, M.H., and Coleman, E. The relationship between homosexuality, internalized homo-negativity, and mental health in men who have sex with men. Journal of Homosexuality, 2008, 55, 185-203.
367. Williams, M.L., Bowen, A.M., Pallonen, U., **Ross, M.W**., McCurdy, S.A., Timpson, S.C., and Amos, C. An investigation of a personal norm of condom use responsibility. AIDS Care, 2008, 20, 225-234.
368. Ross, M.W., Diamond, P., Liebling, A. and Saylor, W. Measurement of prison climate: A comparison of an inmate measure in England and the U.S. Punishment and Society, 2008, 10, 447-474.
369. Begley, K., McLaws, M.L., Ross, M.W. and Gold, J. Cognitive and behavioral correlates of non-adherence to HIV antiretroviral therapy: Theoretical and practical insight for clinical psychology and health psychology. Clinical Psychologist, 2008, 12, 9-17.

370. Daneback, K., Ross, M.W., and Månsson, S-A. Bisexuality and sexually related activities on the Internet. Journal of Bisexuality, 2008, 8, 115-129.
371. Nilsson-Schönnesson, L., Williams, M.L., Atkinson, J., Bowen, A., Ross, M.W., and Timpson, S.C. A Cluster Analysis of Drug Use and Sexual HIV Risks and Their Correlates in a Sample of African-American Crack Cocaine Smokers with HIV Infection. Drug and Alcohol Dependence, 2008, 97, 44-53.
372. Ngo, D.A., Ross M.W., and Ratliff, E. Internet influences on sexual practices among young people in Hanoi, Vietnam. Culture, Health and Sexuality, 2008, 10(Suppl.), S201-213.
373. Ross, M.W., McCurdy, S.A., Kilonzo, G.P., Williams, M.L., and Leshabari, M.T. Drug use careers and blood-borne pathogen risk behavior in Male and Female Tanzanian heroin injectors. American Journal of Tropical Medicine and Hygeine, 2008, 79, 338-343.
374. Peters, R.J., Williams, M.L., Ross, M.W., Atkinson, J., and McCurdy, S. The use Fry (embalming fluid and PCP – laced cigarettes or marijuana sticks) among crack cocaine smokers. Journal of Drug Education, 2008, 38, 285-295.
375. Ross, M.W., Rosser, B.R.S., Neumaier, E.R., and the Positive Connections Team. The relationship of internalized homonegativity to unsafe sexual behavior in HIV seropositive men who have sex with men. *AIDS Education and Prevention*, 2008, *20*, 547-557.
376. Rosser, B.R.S., Bockting, W.O., **Ross, M.W.**, Miner, M.H., and Coleman, E. The relationship between homosexuality, internalized homonegativity, and mental health in men who have sex with men. *Journal of Homosexuality*, 2008, *55*, 185-203.
377. Timpson S., Ratliff E., **Ross M.W**., Williams M., Atkinson J., Bowen A., and McCurdy S. A psychosocial comparison of New Orleans and Houston crack smokers in the wake of Hurricane Katrina. *Substance Use and Misuse*, 2009, 1695-1710.
378. Rosser, B.R.S., Miner, M.H., Bockting, W.O., **Ross, M.W.**, Konstan, J., Gurak, L., Stanton, J., Edwards, W., Jacoby, S.,Carballo-Diéguez, A., Mazin, R., and Coleman, E. HIV Risk and the Internet: Results of the Men's INTernet Study (MINTS). *AIDS and Behavior*, 2009, *13*, 746-756.
379. Pallonen, U.E., Timpson, S.C., Williams, M.L., and **Ross, M.W.** Stages of condom use, partner intimacy, and condom use attitude and self-efficacy in African American crack cocaine users. *Archives of Sexual Behavior*, 2009, 38, 149–158.
380. **Ross, M.W.** The last book-burning trial of the 20th century: The Stålström dissertation and the challenge of homophobic authority. *Journal of Homosexuality*, 2009, 56, 623-638.
381. Ngo, D.A., **Ross, M.W.**, Phan, H., Ratliff, E.A., Trinh, T. and Sherburne, L. Male homosexual identities, relationships, and practices among young men who have sex with men in Vietnam: Implications for HIV prevention. *AIDS Education and Prevention*, 2009, 21, 251-265.
382. Kamiru H, **Ross, M.W.**, Bartholomew, L.K., McCurdy, S.A., and Kline, M.W. Effectiveness of a training program to increase the capacity of health care providers to provide HIV/AIDS care and treatment in Swaziland. *AIDS Care*, 2009, 21, 1463-1470.
383. Brennan, D.J., Welles, S.L., Miner, M.H., **Ross, M.W.**, Mayer, K.H., and Rosser, B.R.S. Development of a treatment optimism scale for HIV-positive gay and bisexual men. *AIDS Care*, 2009, 21,1090-1097.
384. Wilkerson, J.M., Brooks, A., and **Ross, M.W.** Sociosexual Identity Development and Sexual Risk Taking of Acculturating Collegiate Gay and Bisexual Men. *Journal of College Student Development*, 2009, 6, 71-87.
385. Damani, R., **Ross, M.W.**, Aral, S.O., Berman, S., St Lawrence, J., and Williams, M.L. Emotional intimacy predicts condom use: Findings in a group at high STD risk. *International Journal of AIDS and STDs*, 2009, 20, 761-764.
386. Langanke, H., and **Ross, M.W.** Web-based forums for clients of female sex workers: Development of a German Internet approach to HIV/STD-related sexual safety. *International Journal of STD and AIDS*, 2009, 20, 4-8
387. Williams, M.L., McCurdy, S.A., Bowen, A.M., Kilonzo, G.P., Atkinson, J.S., **Ross, M.W.**, and Leshabari, M.T. HIV seroprevalence in a sample of Tanzanian intravenous drug users. *AIDS Education and Prevention*, 2009, 21, 474-483.

388. Hobdell, M., Tsakos, G., Sprod, A., Ladrillo, T.E., **Ross, M.W.**, Gordon, N., Myburgh, N., and Lalloo, R. Using an oral health-related quality of life measure in three cultural settings. *International Dental Journal*, 2009, 59, 381-388.
389. Smolenski, D.J., **Ross, M.W.**, Risser, J.M., and Rosser, B.R.S. Sexual compulsivity and high-risk sex among Latino men: The role of internalized homonegativity and gay organizations. *AIDS Care*, 2009, 21, 42-49.
390. Arrivillaga, M., **Ross, M.W.**, Useche, B., Alzate, M.L. and Correa, D. Social position, gender role, and adherence to treatment among Colombian women with HIV/AIDS: Social determinants of health approach. *Panamerican Journal of Public Health*, 2009, 26, 502-510.
391. Springer, A.E., McQueen, A., Quintenilla, G., Arrivillaga, M., and **Ross, M.W.** Reliability and validity of the Student Perceptions of School Cohesion Scale in a sample of Salvadoran secondary school students. *BMC International Health and Human Rights*, 2009, 9, 1-9.
392. Coleman, E., Horvath, K.J., Miner, M.H., **Ross, M.W.**, Oakes, J.M., Rosser, B.R.S., and the Men's INTernet Sex (MINTS-II) Team. Compulsive sexual behavior and risk for unsafe sex among men who use the internet to seek sex with men. *Archives of Sexual Behavior*, 2010, 39, 1045-1053.
393. **Ross, M.W.** Pedagogy for prisoners: An approach to peer health education for inmates. *Journal of Correctional Health Care*, 2011, 17, 6-18.
394. Nepal, V.P. and **Ross, M.W.** Issues related to HIV stigma in Nepal. *International Journal of Sexual Health*, 2010, 22, 20-31.
395. **Ross, M.W.**, Smolenski, D.J., Kajubi, P., Mandel, J.S., McFarland, W., and Raymond, H.F. Measurement of internalized homonegativity in gay and bisexual men in Uganda: Cross-cultural properties of the Internalized Homonegativity scale. *Psychology, Health and Medicine*, 2010, 15, 159-165.
396. **Ross, M.W.** Prison staff occupational health and safety and its relationship with inmate health: A review. *Prison Service Journal*, 2010, 192, 55-59.
397. McCurdy, S., **Ross, M.W.**, Williams, M.L., Kilonzo, G.P., and Leshabari, M.T. Flashblood: Blood sharing among female injecting drug users in Tanzania. *Addiction*, 2010, 105, 1062-1070.
398. Bui, T., Diamond, P. M., Markham, C., **Ross, M. W.**, Nguyen-Le, T. & Tran, L. Gender relations and sexual communication among female students in the Mekong River Delta of Vietnam. *Culture, Health and Sexuality*, 2010, 12, 591-601.
399. Rochon, D, **Ross, M.W.**, Looney C., Nepal, V.P., Price, A.J., Giordano, T.P. Communication Strategies to Improve HIV Treatment Adherence. *Health Communication*, 2011, 26, 461-467.
400. Brennan, D.J., Welles, S. L., Miner, M. H., **Ross M. W.**, Mayer. K. H., and Rosser, B.R.S. HIV treatment optimism and sexual risk among a national, diverse sample of HIV-positive gay and bisexual men. *AIDS Education and Prevention*, 2010, 22, 126-137.
401. **Ross, M.W.**, and Rosser, B.R.S. The importance of measuring internalized homophobia/homonegativity: Some caveats. *Archives of Sexual Behavior*, 2010, 39, 1207-1208.
402. Smolenski, D.J., Diamond, P.M., **Ross, M.W.**, and Rosser, B.R.S. Revision, criterion validity, and multi-group assessment of the Reactions to Homosexuality scale. *Journal of Personality Assessment*, 2010, 92, 568-576.
403. Swe, H.M. and **Ross, M.W.** Refugees from Myanmar and their health needs in the US: A qualitative study at a refugee resettlement agency. *International Journal of Migration, Health and Social Care*, 2010, 6, 15-25.
404. Rhodes, S.D., Vissman, A.T., Stowers, J., Miller, C., McCoy, T.P., Hergenrather, K.C., Wilkin, A.M., Reece, M., Bachmann, L.H., Ore, A., **Ross, M.W.**, Hendrix, E., and Eng, E. A CBPR partnership increases HIV testing among men who have sex with men: Outcome findings from a pilot test of the CyBER/testing Internet intervention. *Health Education and Behavior*, 2011, 38, 311-320.

405. Lee, D.H., and **Ross, M.W.** Management of human resources associated with misuse of prescription drugs: Analysis of a national survey. *Journal of Health and Human Services Administration*, 2011, 34, 182-205.
406. Smolenski, D.J., Stigler, M.H., **Ross, M.W.**, and Rosser, B.R.S. Direct and indirect associations between internalized homonegativity and high-risk sex. *Archives of Sexual Behavior*, 2011, 40, 785-792.
407. **Ross, M.W.**, Liebling, A., and Tait, S. The Relationships of Prison Climate to Health Service in Correctional Environments: Inmate Healthcare Measurement, Satisfaction and Access in Prisons. *Howard Journal of Criminal Justice*, 2011, 50, 262-274.
408. Sharp, C., Skinner, D., Serekoane, M., and **Ross, M.W.** A qualitative study of the cultural appropriateness of the Diagnostic Interview Schedule for Children (DISC-IV) in South Africa. *Social Psychiatry and Psychiatric Epidemiology*, 2011, 46, 743-751.
409. Williams, M.L., Bowen, A., Atkinson, J., Nilsson Schönnesson, L., Diamond, P., **Ross,** M.W., and Pallonen, U. An assessment of brief group interventions to increase condom use by heterosexual crack smokers living with HIV infection. *AIDS Care*, 2012, *24*, 220-231, DOI: 10.1080/09540121.2011.597707.
410. Graham, J.L., Giordano, T.P., Grimes, R.M., Slomka, J., **Ross, M.W.**, and Hwang, L-Y. Influence of trust on HIV diagnosis and care practices. *Journal of the International Association of Physicians in AIDS Care*, 2011, 9, 346-352. DOI: 10.1177/1545109710380461
411. Hill, M., **Ross, M.W.**, Peters, R., Markham, C., and Granado, M. Too Sad to Care: The relationship between depression related symptoms and delay in seeking medical care. 2011. *Texas Public Health Journal*, 63, 21-27.
412. Machine, E.M., **Ross, M.W.**, and McCurdy, S.A. Issues of expressed stigma of HIV/AIDS among professionals in Southern Sudan. *Qualitative Health Research*, 2011, DOI:10.1177/1049732310392385
413. Daneback, K., Månsson, S-A., and **Ross, M.W.** Online sex shops: Purchasing sexual merchandise on the internet. *International Journal of Sexual Health*, 2011, 23, 102-110.
414. Berg, R., Tikkanen, R., and **Ross, M.W.** Predictors of reporting bareback sex among a diverse sample of MSM recruited through a Swedish website. *AIDS Care*, 2011, 1644-1651, DOI:10.1080/09540121.2011.565035
415. **Ross, M.W.**, Månsson, S-A., and Daneback, K. Prevalence, severity and correlates of problematic sexual Internet use in Swedish men and women. *Archives of Sexual Behavior*, 2012, 41, 459-466. DOI: 10.1007/s1007-011-9762-0.
416. Arrivillaga, M., **Ross, M.W.**, Useche, B., Springer, A. and Correa, D. Applying an expanded social determinant approach to the concept of adherence to treatment: The case of Colombian women living with AIDS. *Women's Health Issues*, 2011, 21, 177-183. DOI: 10.1016/j.whi.2010.09.003
417. Welles, S., **Ross, M.W.**, Banik, S., Fisher, L., McFarlane, M.M., Kachur, R., Rietmeijer, C., and Allensworth-Davies, D. Demographic and sexual behavior comparisons of Indian and United States Internet samples of Men who have Sex with Men. *International Journal of Sexual Health*, 2011, 23, 90-101.
418. Berg, R.C., **Ross, M.W.**, and Tikkanen, R. The effectiveness of MI4MSM: How useful is Motivational Interviewing as an HIV risk prevention program for men who have sex with men? A systematic review. *AIDS Education and Prevention*, 2011, 23, 533-549. doi: 10.1521/aeap.2011.23.6.533
419. Daneback, K., Månsson, S-A., **Ross, M.W.**, and Markham, C. The Internet as a source of information about sexuality. *Sex Education*, 2012, 12, 583-598. DOI:10.1080/14681811.2011.627739
420. Wong, F.Y., Nehl, E.J., Han, J.J., Huang, Z.J., Wu, Y., Young, D., **Ross, M.W.** and The MATH Study Consortium. Findings of HIV testing and HIV management from a national sample of Asian/Pacific Islander Men Who Have Sex with Men. *Public Health Reports*, 2012, 127, 186-194.

421. **Ross, M.W.**, Crisp, B.R., Månsson, S-A., and Hawkes, S. Occupational health and safety in commercial sex workers. *Scandinavian Journal of Work, Environment and Health*, 2012, 38, 105-119.
422. Graham, J.L., Grimes, R.M., Slomka, J., **Ross, M.W.**, Hwang, L.Y., and Giordano, T.P. The role of trust in delayed HIV diagnosis in a diverse, urban population. *AIDS and Behavior*, 2013, 17, 266-273. DOI 10.1007/s10461-011-0114-9.
423. **Ross, M.W.**, Daneback, K., and Månsson, S-A. Fluid versus fixed: A new perspective on bisexuality as a fluid sexual orientation beyond gender. *Journal of Bisexuality*, 2012, 12, 449-460. DOI:10.1080/15299716.2012.702609
424. Arrivillaga, M.Q., Springer, A.E., Lopera, M., Correa, D., Useche, B., and **Ross, M.W.** HIV/AIDS treatment adherence in economically better off women in Colombia. *AIDS Care*, 2012, 24, 929-935. DOI 10.1080/09540121.2011.647678
425. Selvaraj, V., **Ross, M.W.,** Unnikrishnan, B. and Hegde, S. Association of quality of life with major depression disorder among people with HIV in South India. *AIDS Care*, 2013, 25, 169-172. DOI:10.1080/09540121.2012.689809
426. Bui, T.C., Markham, C.M., **Ross, M.W.**, Williams, M.L., Beasley R.P., Tran, L.T.H., Nguyen, H.T.H., Le, T.N. Perceived gender inequality, sexual communication self-efficacy, and sexual behaviour among female undergraduate students in the Mekong Delta of Vietnam. *Sexual Health*, 2012, 9, 314-322. DOI 10.1071/SH11067
427. Nyoni, J., and **Ross, M.W.** Factors Associated with HIV Testing in Men who have Sex with Men in Dar es Salaam, Tanzania. *Sexually Transmissible Infections*, 2012, 88, 483. doi:10.1136/sextrans-2012-050661.
428. Nyoni, J., and **Ross, M.W.** Condom Use and HIV-related behaviours in Urban Tanzanian Men who have Sex with Men: A study of beliefs, HIV knowledge sources, and partner interactions. *AIDS Care*, 2012, DOI:10.1080/09540121.2012.699671.
429. Daneback, K., Sevcikova, A., Månsson, S-A., and **Ross, M.W.** Outcomes of using the internet for sexual purposes: fulfillment of sexual desires. *Sexual Health*, 2013, 10, 26-31. http://dx.doi.org/10.1071/SH11023,
430. Daneback, K., Månsson, S-A., and **Ross, M.W.** Technological Advancements and Internet Sexuality: Does Private Access to the Internet Influence Online Sexual Behavior? *Cyberpsychology, Behavior, and Social Networking*, 2012, 15, 386-390. doi:10.1089/cyber.2011.0188
431. Giordano TP, Rodriguez S, Zhang H, Kallen MA, Jibaja-Weiss M, Buscher AL, Arya M, Suarez-Almazor ME, and **Ross M.W.** Effect of a clinic-wide social marketing campaign to improve adherence to antiretroviral therapy for HIV infection. *AIDS and Behavior*, 2012, DOI 10.1007/s10461-012-0295-x
432. Millimet, S.A., Miller, B., **Ross, M.W.**, Samson, P., and Chaitanya Churi, C. Results of an HIV stigma reduction intervention on a university campus. *International Journal of Child and Adolescent Health*, 2013, 6(3), 297-304.
433. **Ross, M.W.**, and Harzke, A.J. Toward healthy prisons: the TECH model and its applications. International Journal of Prisoner Health, 2012, 8, 16-26. DOI: 10.1108/17449201211268255
434. **Ross M.W.**, Kajubi, P., Mandel, J.S., McFarland, W., Raymond, H.F. Internalized Homonegativity/Homophobia is associated with HIV Risk Behaviors in Ugandan Gay and Bisexual Men. *International Journal of STDs and AIDS*, 2013, 24, 409-413. doi: 10.1177/0956462412472793.
435. Fujimoto, K., Williams, M.L., and **Ross, M.W.** Venue-based affiliation network and HIV risk behavior among male sex workers. *Sexually Transmitted Diseases*, 2013, 40(6), 453-458.
436. **Ross, M.W.**, Nyoni, J.E., Williams, M.E., Bowen, A. M., and Kashiha, J.J. The Sexual and Geographic Organization of Men who have Sex with Men in a large East African City: Opportunities for outreach. *British Medical Journal*, 2012, **2**:e001813 doi:10.1136/bmjopen-2012-001813
437. **Ross, M.W.,** Berg, R.C., Schmidt, A.J., Hospers, H.J., Breveglieri, M., Furegato, M., Weatherburn, P. and the European MSM Internet Survey (EMIS) Network. Internalised

Homonegativity predicts HIV-Associated Risk Behavior in European Men who have Sex with Men in a 38-Country Cross-sectional Study: Some Public Health Implications of Homophobia. *British Medical Journal*, 2013, 3:e001928. Doi10.1136/bmjopen-2012-001928.
438. Bui, T. C., Markham, C. M., Tran, L. T. H., Beasley, R. P., and **Ross, M. W.** Condom Negotiation and Use Among Female Sex Workers in Phnom Penh, Cambodia. *AIDS and Behavior*, 2012, 17, 612-622. DOI 10.1007/s10461-012-0369-9. PMID: 23196858.
439. Berg R.C., **Ross, M.W.**, Schmidt, A.J., and Weatherburn, P. Structural and environmental factors are associated with internalised homonegativity in men who have sex with men: Findings from the European MSM Internet Survey (EMIS) in 38 countries. *Social Science and Medicine*, 2013, 61-69, http://dx.doi.org/10.1016/j.socscimed.2012.11.033
440. Berg, R.C., Tikkanen, R., and **Ross, M.W.** Barebacking among men who have sex with men recruited through a Swedish website: Associations with sexual activities at last sexual encounter. *Eurosurveillance*, 2013, 18:13, http://www.eurosurveillance.org/ViewArticle.aspx?ArticleId=20438
441. Hill, M., Hallmark, C., McNeese-Ward, M., Blue, N., and **Ross, M.W.** HIP HOP for HIV Awareness: Using hip hop culture to promote community level HIV prevention. *Sex Education*, DOI:10.1080/14681811.2013.852079
442. L. Huang, E.J. Nehl, L. Lin, G. Meng, Q. Liu, **Ross, M.W** and F.Y. Wong , AIDS Care (2013): Sociodemographic and sexual behavior characteristics of an online MSM sample in Guangdong, China, *AIDS Care*, 2013, DOI: 10.1080/09540121.2013.844760
443. **Noor, S**., Ross, M., Lai, D., and Risser, J. Knowledge of HIV serostatus and HIV risk: Unaware status is associated with drug and sexual risk behavior among injection drug users. *International Journal of STD & AIDS,* ePub on 24 July, 2013. DOI: 10.1177/0956462413496079
444. Nehl, E.J., Talley, C.L., Ong, P.M., Takahashi, L.M., Yu, F., Nakayama, K.K., **Ross, M.W.**, The MATH Consortium & Wong, F.Y. Dating preferences among self-identified gay men of Asian descent in the United States. *Asian American Journal of Psychology,* 2014, 5(4), 335-343. doi:10.1037/a0035539
445. Huy Ha, **Ross, M.W.**, Risser, J.M.H., and Nguyen, H.T.M. Measurement of Stigma in Men Who Have Sex with Men in Hanoi, Vietnam: Assessment of a Homosexuality-Related Stigma Scale, *Journal of Sexually Transmitted Diseases*, 2013, Article ID 174506. doi:10.1155/2013/174506.
446. Berg, R.C., and Ross, M.W. The second closet: A qualitative study of HIV stigma among seropositive gay men in a southern U.S. city. *International Journal of Sexual Health*, 2013, **DOI:** 10.1080/19317611.2013.853720
447. Langanke, H, Månsson S-A., and Ross, M.W. Planning for pleasure: Time patterns in the use of Internet forums of female sex workers' clients in Germany. *Cyberpsychology*, 2014, *8*(1), Article: 5: doi: 10.5817/CP2014-1-5
448. **Ross, M.W.,** Tikkanen, R. and Berg, R.M. Gay Community Involvement: Its Inter-relationships and Associations with Internet Use and HIV risk Behaviors in Swedish Men who have Sex with Men. *Journal of Homosexuality*, 2014, 61, 323-333. DOI: 10.1080/00918369.2013.839916
449. Hill, M., Granado, M., Peters, R., Markham, C., **Ross, M.W.**, & Grimes, R. A pilot study on the use of a Smartphone application to encourage emergency department patients to access preventive services: Human papillomavirus vaccine as an example. *Emergency Medicine and Health Care*, 2013, 1(1), 4. doi:10.7243/2052-6229-1-4
450. Rice, S.R., and **Ross, M.W.** Differential Processes of 'Internet' versus 'Real Life' Sexual Filtering and Contact among Men who have Sex with Men. *Cyberpsychology*, 2014, *8*(1), Article: 6: doi: 10.5817/CP2014-1-6
451. Bui TC, Tran LT, **Ross MW**, Markham CM. Douching practices among female sex workers in Phnom Penh, Cambodia. *International Journal of STD & AIDS*. Accepted for publication.

452. Bui T.C., Nyoni, J.E., **Ross, M.W.**, Mbwambo, J., Markham, C.M., and McCurdy, S.A. Sexual motivation, sexual transactions and sexual risk behavior in men who have sex with men in Dar es Salaam, Tanzania. *AIDS and Behavior*, 2014, DOI 10.1007/s10461-014-0808-x
453. **Ross, M.W.** and Anderson, A.M. Relationships between importance of religious belief, response to anti-gay violence, and mental health in men who have sex with men in East Africa. *Research in the Social Scientific Study of Religion*, 2014, 25:160-172.
454. Anderson, A.M., **Ross, M.W.**, and Nyoni, M.W. High prevalence of stigma-related abuse among a sample of men who have sex with men in Tanzania: implications for HIV prevention. *AIDS Care*, 2014, DOI: 10.1080/09540121.2014.951597
455. **Ross, M.W.**, Nyoni, J,, Ahaneku, H.O., Mbwambo, J., McClelland, R.S., and McCurdy, S.A. High HIV seroprevalence, rectal STIs and risky sexual behaviour in men who have sex with men in Dar es Salaam and Tanga, Tanzania. *BMJ Open* 2014;4:e006175. doi:10.1136/bmjopen-2014-006175
456. Fujimoto, K., Williams, M.L., and **Ross, M.W**. A Network Analysis of Relationship Dynamics in Sexual Dyads as Correlates of HIV Risk Misperceptions among High-risk MSM. Sexually Transmitted Infections, 2015, 91(2), 130-134. doi:10.1136/sextrans-2014-051742
457. Fujimoto, K., Wang, P., **Ross, M.W**., and Williams, M.L. Venue-Mediated Weak Ties in Multiplex HIV Transmission Risk Networks Among Drug-Using Male Sex Workers and Associates. American Journal of Public Health, 2015, 105(6), 1128-1135. doi:10.2105/AJPH.2014.302474
458. **Ross, M.W.**, Nyoni, J., Larsson, M., Mbwambo, J., Agardh, A., Kashiha, J. and McCurdy, S.A. Health Care in a Homophobic Climate: The SPEND Model for providing sexual health services to men who have sex with men where their health and human rights are compromised. *Global Health Action*, 2015, 8:26096 - http://dx.doi.org/10.3402/gha.v8.26096.
459. Berg, R.C., Weatherburn, P. B., **Ross, M.W.**, Schmidt, A.J., the EMIS Network. The relationship of internalised homonegativity to sexual health and well-being among men who have sex with men in 38 European countries. *Journal of Gay and Lesbian Mental Health*, 2015, 19(3), 285-302. doi:10.1080/19359705.2015.1024375
460. Morandini, J.S., Blaszczynski, A., **Ross, M.W.**, Costa, D.S.J., and Dar-Nimrod, I. Essentialist beliefs, sexual identity uncertainty, internalised homonegativity and psychological wellbeing in gay men. *Journal of Counseling Psychology*, 2015, 62(3), 413-424. doi:10.1037/cou0000072
461. Sheek-Hussein, M.M., **Ross, M.W.**, Nagelkerke, N., Alsuwaidi, A.R., Sayenna Uduman, S., and Souid, A-K. Natural History of Vertically Transmitted Hepatitis C Virus. *SM Journal of Hepatitis Research and Treatment*, 2015, 1(1):1004.
462. Berg, R.C., Munthe-Kaas, H.M., and **Ross, M.W.** Internalized homonegativity: A systematic mapping review of empirical research. *Journal of Homosexuality*, 2016, 63(4), 541-558. doi:10.1080/00918369.2015.1083788
463. Romijinders, K.A., Nyoni, J.E., **Ross, M.W.**, McCurdy, S.A., Mbwambo, J., Kok, G., & Crutzen, R. (2016). Lubricant use and condom use during anal sex in Men who have sex with Men in Tanzania. *International Journal of STD & AIDS, 27*(14), 1289-1302. doi:10.1177/0956462415615067
464. Agardh, A., **Ross, M.W.**, Östergren, P-O., Larsson, M., Tumwine, G., Månsson, S-A., Simpson, J.A., and Patton, G. (2016). Health risks in same-sex attracted Ugandan university students: evidence from two cross-sectional studies. *PLoS One*, *11*(3), e0150627. doi:10.1371/journal.pone.0150627
465. Ahaneku, H., **Ross, M.W.**, Nyoni, J.E., Selwyn, B., Troisi, C., Mbwambo, J., Adeboye, A, and McCurdy, S. (2016). Depression and HIV risk among men who have sex with men in Tanzania. *AIDS Care*, *28*(suppl. 1), 140-147. doi:10.1080/09540121.2016.1146207
466. Aboul-Enein, B.H., Bernstein, J., and **Ross, M.W.** (2016). Evidence for masturbation and prostate cancer risk: Do we have a verdict? *Sexual Medicine Reviews*, *4*(3), 229-234. doi:10.1016/j.sxmr.2016.02.006
467. Rhodes, S.D., McCoy, T.P., Tanner, A.E., Stowers, J., Bachmann, L.H., Nguyen, A.L., & **Ross, M.W.** (2016). Using social media to increase HIV testing among gay and bisexual men,

other men who have sex with men, and transgender persons: Outcomes from a randomized community trial. *Clinical Infectious Diseases*, *62*(11), 1450-1453. doi:10.1093/cid/ciw127

468. Larsson, M., **Ross MW**, Månsson S-A, Nyoni, JE, Shio, J, & Agardh, A. (2016). Being forced to become your own doctor: Men who have sex with men's experiences of stigma in the Tanzanian healthcare system. *International Journal of Sexual Health*, *28*(2), 163-175. doi:10.1080/19317611.2016.1158763.
469. Hallmark, C.J., Hill, M.J., Luswata, C., Watkins, K.L., Thornton, L., McNeese, M., & **Ross, M.W.** (2016). Déjà vu? A Comparison of Syphilis Outbreaks in Houston, Texas. *Sexually Transmitted Diseases, 43*(9), 549-555. doi:10.1097/OLQ.0000000000000488
470. Okudo, J.C., & **Ross, M.W.** (2016). Common health concerns in African immigrants in the US-Implications for the family physician. *International Journal of Medical and Health Sciences Research, 3*(4), 44-49. doi:10.18488/journal.9/2016.3.4/9.4.44.49
471. Fujimoto, K., Kim, J.Y., **Ross, M.W.**, & Williams, M.L. (2016). Multiplex crack smoking and sexual networks: Associations between network members' incarceration and HIV risks among high-risk MSM. *Journal of Behavioral Medicine*, *39*(5), 845-854. doi:10.1007/s10865-016-9754-6
472. Machine, E.M., Gillespie, S.L., Homedes, N., Selwyn, B.J., **Ross, M.W.**, Anabwani, G., Schutze, G., & Kline, M.W. Lost to follow-up: Failure to engage children in care in the first three months of diagnosis. *AIDS Care*, *28*(11), 1402-1410. doi:10.1080/09540121.2016.1179714
473. Larsson, M., Odberg, K., Kashiha, J., **Ross, M.W.**, & Agardh, A. (2016). Stretching the boundaries: Tanzanian pharmacy workers' views and experiences of providing STI services for men who have sex with men. *PLOSOne*, *11*(11), e0166019. doi:10.1371/journal.pone.0166019
474. **Ross, M.W.**, Larsson, M., Jacobson, J., Nyoni, J., & Agardh, A. (2016). Social networks of men who have sex with men and their implications for HIV/STI interventions: Results from a cross-sectional study using respondent-driven sampling in a large and a small city in Tanzania. *BMJ Open*, *6*(11), e012072. doi:10.1136/bmjopen-2016-012072
475. Bui, T.C., Markham, C.M., Diamond, P.M., Tran, L.T., **Ross, M.W.**, Nguyen, H.T., & Le, T.N. (2017). Quantitative measure of self-perceived gender relations in young women in the Mekong Delta of Vietnam. *Women's Health Bulletin*, *4*(1). doi:10.17795/whb-38488
476. Smith, C., White, M., & **Ross, M.W.** (2017). Experience of online outreach for Black MSM populations at high risk for HIV transmission. *Journal of the Association of Nurses in AIDS Care*, *28*(1), 25-26. doi:10.1016/j.jana.2016.09.009
477. Fujimoto, K., Wang, P., Kuhns, L. M., **Ross, M. W.**, Williams, M. L., Garofalo, R., Klovdahl, A. S., Laumann, E. O., & Schneider, J. A. (2017). Multiplex competition, collaboration, and funding networks among health and social organizations: Toward organization-based HIV interventions for young men who have sex with men. *Medical Care*, 55(2), 102-110. doi:10.1097/MLR.0000000000000595
478. Berg, R.C., Lemke, R., & **Ross, M.W.** (2017). Sociopolitical and cultural correlates of internalized homonegativity in gay and bisexual men: Findings from a global study. *International Journal of Sexual Health*, *29*(1), 97-111. doi:10.1080/19317611.2016.1247125
479. Andrade, R., Rodriguez-Barradas, M.C., Yasukawa, K., Villarreal, E., **Ross, M.W.**, & Serpa, J.A. (2017). Single versus 3 doses of intramuscular benzathine penicillin for early syphilis in HIV: A randomized clinical trial. *Clinical Infectious Diseases*, *64*(6), 759-764. doi:10.1093/cid/ciw862
480. **Ross, M.W.** (2017). Truth be told: Evaluation of a narrative and skills intervention in two women's prisons. *Journal of Correctional Health*, 23(2), 184-192. doi:10.1177/1078345817704348
481. Berg, R.C., Carter, D., & **Ross, M.W.** (2017). A mixed-method study on correlates of HIV-related stigma among gay and bisexual men in the southern United States. *Journal of the Association of Nurses in AIDS Care*, *28*(4), 532-544. doi:10.1016/j.jana.2017.02.004
482. **Ross, M. W.**, Larsson, M., Nyoni, J., & Agardh, A. (2017). Prevalence of STI symptoms and high levels of stigma in STI healthcare among men who have sex with men in Dar es Salaam,

Tanzania: A respondent-driven sampling study. *International Journal of STD & AIDS*, *28*(9), 925-928. doi:10.1177/0956462416683625

483. Larsson, M., Mohamed Shio, J., **Ross M. W.**, & Agardh, A. (2017). Acting within an increasingly confined space: a qualitative study of sexual behaviours and healthcare needs among men who have sex with men in a provincial Tanzanian city. *Plos One*, *12*(8), e0183265. doi:10.1371/journal.pone.0183265
484. Butame, S.A., Lawler, S., Hicks, J.T., Wilkerson, J.M., Hwang, L., Baraniuk, S., **Ross, M.W.**, Chiao, E.Y., & Nyitray, A.G. (2017). A qualitative investigation among men who have sex with men on the acceptability of performing a self- or partner anal exam to screen for anal cancer. *Cancer Causes & Control*, *28*(10), 1157-1166. doi:10.1007/s10552-017-0935-6
485. Adeboye, A., **Ross, M. W.**, Wilkerson, J. M., Springer, A., Ahaneku, H., Yusuf, R. A., Olanipekun, T. O., & McCurdy, S. (2017). Resilience factors as a buffer against the effects of syndemic conditions on HIV risk and infection among Tanzanian MSM. *Journal of Health Education Research & Development*, *5*(3), 230. doi:10.4172/2380-5439.1000232
486. Adeboye, A., **Ross, M. W.**, Wilkerson, J. M., Springer, A., Ahaneku, H., Yusuf, R. A., Olanipekun, T. O., & McCurdy, S. (2017). Syndemic production of HIV infection among Tanzanian MSM. *Journal of Health Education Research & Development*, *5*(3), 231. doi:10.4172/2380-5439.1000231
487. Agardh, C., Weije, F., Agardh, A., Nyoni, J, **Ross, M. W.**, Kashiha, J., & Larsson, M. (2017). Using pharmacists and drugstore workers as sexual healthcare givers: A qualitative study of men who have sex with men in Dar es Salaam, Tanzania. *Global Health Action*, *10*(1), 1389181. doi:10.1080/16549716.2017.1389181
488. Nyitray, A. G., Hicks, J. T., Hwang, L-Y, Baraniuk, S., White, M., Millas, S., Onwuka, M., Zhang, X., Brown, E. L., **Ross, M. W.**, & Chiao, E. Y. (2018). A phase II clinical study to assess the feasibility of self- and partner anal exams to detect anal canal abnormalities including anal cancer. *Sexually Transmitted Infections*, *94*(2), 124-130. doi:10.1136/sextrans-2017-053283
489. **Ross, M.W.**, Leshabari, S., Rosser, B.R.S., Trent, M., Mgopa, L., Wadley, J., Kohli, N., & Agardh, A. (2018). Evaluation of an assessment instrument for a sexual health curriculum for nurses and midwifery students in Tanzania: The Sexual Health Education for Professionals Scale (SHEPS). *Applied Nursing Research*, *40*, 152-156. doi:10.1016/j.apnr.2018.01.005
490. **Ross, M. W.**, Iguchi, M., & Panicker, S. (2018). Ethical aspects of data sharing and research participant protections. *American Psychologist*, *73*(2), 138-145. doi:10.1037/amp0000240
491. Tran, H., **Ross, M. W.**, Diamond, P. M., Berg, R. C., Weatherburn, P., & Schmidt, A. J. (2018). Structural validation and multiple group assessment of the Short Internalized Homonegativity Scale in homosexual and bisexual men in 38 European countries: Results from the European MSM Internet Survey. *Journal of Sex Research*, *55*(4-5), 617-629. doi:10.1080/00224499.2017.1380158
492. Nyitray, A. G., Bagyinszky, F., **Ross, M. W.**, & Schmidt, A .J. (2018). Lack of HIV testing and dissatisfaction with HIV testing and counselling among men having sex with men in Hungary. *European Journal of Public Health*, *28*(4), 713-720. doi:10.1093/eurpub/ckx200
493. Nilsson Schönnesson, L., Zeluf, G., Garcia-Huidobro, D., **Ross, M. W.**, Eriksson, L. E., & Ekström, A.M. (2018). Sexual (dis)satisfaction and its contributors among people living with HIV infection in Sweden. *Archives of Sexual Behavior*, *47*(7), 2007-2026. doi:10.1007/s10508-017-1106-2
494. Warner, C., Carlson, S., Crichlow, R., & **Ross, M.W.** (2018). Sexual health knowledge of U.S. medical students: A national survey. *Journal of Sexual Medicine*, *15*(8), 1093-1102. doi:10.1016/j.jsxm.2018.05.019
495. Bui, T. C., Scheurer, M. E., Pham, V. T., Tran, L. T., Hor, L. B., Vidrine, D. J., **Ross, M. W.**, & Markham, C. M. (2018). Intravaginal practices and genital human papillomavirus infection among female sex workers in Cambodia. *Journal of Medical Virology*, *90*(11), 1765-1774. doi:10.1002/jmv.25268

496. Lamontagne, E., d'Elbée, M., **Ross, M. W.**, Carroll, A., du Plessis, A., & Loures, L. (2018). A socioecological measurement of homophobia for all countries and its public health impact. *European Journal of Public Health*, *28*(5), 967-972. doi:10.1093/eurpub/cky023
497. McCurdy, S.A., & **Ross, M.W.** (2018). Qualitative data are not just quantitative data with text but data with context: On the dangers of sharing some qualitative data: Comment on DuBois et al. (2018). *Qualitative Psychology*, *5*(3), 409-411. doi:10.1037/qup0000088
498. Bradford, N. J., Dewitt, J., Decker, J., Berg, D. R., Spencer, K.G., & **Ross, M.W.** (2018). Sex education and transgender youth: "Trust means material by and for queer and trans people." *Sex Education*, *19*(1), 84-98. doi:10.1080/14681811.2018.1478808
499. Hicks, J. T., Hwang, L.-Y., Baraniuk, S., White, M., Chiao, E. Y., Onwuka, N., **Ross, M. W.**, & Nyitray, A. G. (online first). Factors associated with self-reported anal cancer screening history in men who have sex with men. *Sexual Health*, online ahead of print December 6, 2018. doi:10.1071/SH18039
500. **Ross, M. W.**, Kashiha, J., Nyoni, J., Larsson, M.S., & Agardh, A. (online first). Electronic media access and use for sexuality and sexual health education among men who have sex with men in four cities in Tanzania. *International Journal of Sexual Health*, online ahead of print January 31, 2019. doi:10.1080/19317611.2018.1491919

* Also reprinted as chapters with the same title

Chapters

1. Ross, M.W. The ethics of experiments on higher animals. In: Keehn, J.D. (ed.), *The Ethics of Psychological Research*. Oxford: Pergamon, 1982, 51-60.
2. Ross, M.W. Mitä homoseksuaalisuus on? Tieteellisiä näkökantoja [What is homosexuality? The scientific evidence]. In Sievers, K., & Stålström, O.W. (eds.), *Rakkauden Monet Kasvot. Homoseksuaalisesta Rakkaudesta, Ihmisoikeuksista Ja Vapautumisesta*. [The Many Faces of Love. On Homosexual Love, Human Rights and Liberation]. Tapiola: Weilin & Göös, 1984, 161-190.
3. Stålström, O.W., & Ross, M.W. Ihmissuhteet, elämäntavat ja identiteetit [Identities, relationships and lifestyles]. In: Sievers, K., & Stålström, O.W. (eds.), *Rakkauden Monet Kasvot. Homoseksuaalisesta Rakkaudesta. Ihmisoikeuksista Ja Vapautumisesta*. [The Many Faces of Love. On Homosexual Love, Human Rights, and Liberation]. Tapiola: Weilin & Göös, 1984, 38-83.
4. Ross, M.W. Psychovenereology and acquired immune deficiency syndrome. In: Nichols, S.E., & Ostrow, D.G. (eds.), *Psychiatric Implications of Acquired Immune Deficiency Syndrome*. Washington DC: American Psychiatric Press, 1984, 111-121.
5. Ross, M.W. Homosexuality and social sex roles: a re-evaluation. In: Ross, M.W. (ed.), *Homosexuality and Social Sex Roles*. New York: Haworth Press, 1983, 1-6.
6. Ross, M.W. Femininity, masculinity and sexual orientation: some cross-cultural comparisons. In: Ross, M.W. (ed.), *Homosexuality and Social Sex Roles*. New York: Haworth Press, 1983, 27-36.
7. Ross, M.W. Gender identity: male, female or a third gender? In: Walters, W.A.W., & Ross, M.W. (eds.), *Transsexualism and Sex Reassignment*. Melbourne: Oxford University Press, 1986, 1-8.
8. Burnard, D., & Ross, M.W. Psychosocial aspects and psychological testing: What can psychological testing reveal? In: Walters, W.A.W., & Ross, M.W. (eds.), *Transsexualism and Sex Reassignment*. Melbourne: Oxford University Press, 1986, 52-63.

9. Walters, W.A.W., Kennedy, T., & Ross, M.W. Results of gender reassignment: is it all worthwhile? In: Walters, W.A.W., & Ross, M.W. (eds.), *Transsexualism and Sex Reassignment*. Melbourne, Oxford University Press, 1986, 144-151.
10. Ross, M.W. Causes of gender dysphoria: how does transsexualism develop and why? In: Walters, W.A.W., & Ross, M.W. (eds.), *Transsexualism and Sex Reassignment*. Melbourne: Oxford University Press, 1986, 16-25.
11. Ross, M.W. Social and behavioral aspects of male homosexuality. In: Cooney, T.G., & Ward, T.T. (eds.), *AIDS and Other Medical Problems in the Male Homosexual*. Philadelphia: W.B. Saunders, 1986, 537-549.
12. Ross, M.W. Psychosocial aspects of AIDS-related syndromes. In: *Meeting the Challenge: Papers of the First National Conference on AIDS*. Canberra: Australian Government Publishing Service, 1986, 11-15.
13. Ross, M.W. Theory of normal homosexuality: a critique and redefinition of homosexual contacts. In: Diamant, L. (ed.), *Male and Female Homosexuality: Psychological Approaches*. Washington DC: Hemisphere, 1987, 237-259.
14. Ross, M.W. Gay youth in four cultures: a comparitive study. In: Herdt, G.H.(ed.) *Gay Youth*. New York: Harrington Park Press, 1989, 299-314.
15. Ross, M.W., Paulsen, J.A., & Stålström, O.W. Homosexuality and mental health: a cross-cultural review. In: Ross, M.W. (ed.) *The Treatment of Homosexuals with Mental Health Disorders*. New York: Harrington Park Press, 1988, 131-152.
16. Ross, M.W. Ego-dystonic heterosexuality: a case study. In: Ross, M.W. (ed.) *The Treatment of Homosexuals with Mental Health Disorders*. New York: Harrington Park Press, 1988, 7-11.
17. Ross, M.W. Psychological perspectives of human sexuality and sexually transmissible diseases. In: Holmes, K.K., Mårdh, P.A., Sparling, P.F., & Wiesner, P.J. *Sexually Transmitted Diseases (2nd edn)*. New York: McGraw-Hill, 1990, 55-60.
18. Ross, M.W. Psychovenereology of AIDS and sexually transmitted diseases. In: Ostrow, D.G. (ed.) *Behavioral Aspects of AIDS and STDs*. New York: Plenum Press, 1990, 19-40.
19. Ross, M.W. Married homosexual men: prevalence and background. In: Bozett, F.W., & Sussman, M.B. (eds) *Homosexuality and Family Relations*. New York: Harrington Park Press, 1990, 35-57.
20. Ross, M.W. A taxonomy of global behaviour. In: Tielman, R., Carballo, M., & Hendriks, A. (eds) *Bisexuality and AIDS*. Buffalo: Prometheus, 1991, 21-26.
21. Ross, M.W. Male bisexuality in Australia. In: Tielman, R., Carballo, M., & Hendriks, A. (eds) *Bisexuality and AIDS*. Buffalo: Prometheus, 1991, 127-129.
22. Ross, M.W. Bisexuality and injecting drug users. In: Tielman, R., Carballo, M., & Hendriks, A. (eds) *Bisexuality and AIDS*. Buffalo: Prometheus, 1991, 161-163.
23. Ross, M.W., Wodak, A., Miller, M.E., & Gold, J. Attitudes toward termination of pregnancy and associated risk behaviours in drug-injecting women. In: Mélica, F. (ed.) *AIDS and Reproduction*. Basel: Karger, 1992, 55-60.
24. Ross, M.W. Mental health issues of the worker with AIDS or HIV infection. In: Diamant, L. (ed.), *Homosexual Issues in the Workplace*. Washington DC: Hemisphere, 1993.
25. Ross, M.W., & Kelaher, M.A. Knowledge, attitudes and behaviour in heterosexual men and women: the research evidence. In: Sherr, L.(ed.), *Heterosexual Aspects of AIDS*. London: Harwood, 1993, 253-262.
26. Ross, M.W. and McLaws, M-L. The theory of reasoned action: predicting AIDS-preventive behaviour in homosexual men. In: Terry D.J., Gallois, C., & McCamish M.M. (eds.), *The*

Theory of Reasoned Action: Its Application to AIDS-Preventive Behaviour. Oxford: Pergamon, 1993, 81-92.

27. McLaws, M-L., Oldenburg, B., & Ross, M.W. Measurement issues involved in the application of the theory of reasoned action to HIV-preventive behaviour. In: Terry, D.J., Gallois, C., & McCamish M.M. (eds.), *The Theory of Reasoned Action: Its Application to AlDS-Preventive Behaviour*. Oxford: Pergamon, 1993, 169-184.
28. Ross, M.W. AIDS and the new public health. In: Waddell, C., & Petersen, A. (eds.) *Just Health*. Melbourne: Churchill Livingstone, 1994, 323-335.
29. Bennett, L., Ross, M.W., & Kelaher, M.A. The impact of working with HIV/AIDS on health care professionals: dimensions and measurement. In: van Dis, H., & van Dongen, E. (eds), *Burnout in HIV/AIDS Health Care and Support: Impact for Professionals and Volunteers*. Amsterdam: Amsterdam University Press, 1993, 11-21.
30. Bennett, L., Kelaher, M.A., & Ross, M.W. Burnout and coping in HIV/AIDS health care professionals. In van Dis, H., & van Dongen, E. (eds), *Burnout in HIV/AIDS Health Care and Support: Impact for Professionals and Volunteers*. Amsterdam: Amsterdam University Press, 1993, 41-51.
31. Furner, V. & Ross, M.W. Lifestyle clues in the recognition of HIV infection: how to take a sexual history. In Stewart, G. (ed), *Could it be HIV? The Clinical Recognition of HIV Infection*. Sydney: Australasian Medical Publishing Company, 1993, 13-14.
   (*Second Edition*). Sydney: Australasian Medical Publishing Company, 1994, 13-14.
32. Carne, P.T., Ross, M.W., & Kemp, R.J. A practitioner's guide to HIV testing. In Stewart, G. (ed), *Could it be HIV? The Clinical Recognition of HIV Infection*. Sydney: Australasian Medical Publishing Company, 1993, 64-65.
   (*Second Edition*). Sydney: Australasian Medical Publishing Company, 1994, 64-65.
33. Ross, M.W. Parental rearing, borderline thought disorder and suicidal thought disorder in young adults. In: Perris C., Arrindell, W.A., & Eisemann, M. (eds), *Parenting and Psychopathology*. Chichester: Wiley, 1994, 253-265.
34. Richmond, B. & Ross, M.W. Death of a partner: responses to AIDS-related bereavement. In: Sherr, L. (ed.), *Grief and AIDS*. Chichester: Wiley, 1995, 161-179.
35. Ross, M.W., Wodak, A., Gold, J. & Miller, M.E. Unterschiede in der sexuallen orienterung im verhältnis zum HIV-riskoverhalten bei intravenös drogenabhängigen. In: Haeberle, E.J & Gindorf, R. (eds), *Bisexualitäten: Ideologie und Praxis des Sexualkontaktes mit beiden Geschlechtern*. Stuttgart: Gustav Fischer Verlag, 1994, 310-319.
36. Stowe, A., Ross, M.W., Wodak, A., Thomas, G.V., & Larson, S.A. Significant relationships and social supports of injecting drug users and their implications for HIV/AIDS services. In: Bor, R., & Elford, J. (eds), *The Family and HIV*. London: Cassell, 1994, 129-140.
37. Ross, M.W., Fernández-Esquer, M.E. & Seibt, A. Understanding across the sexual orientation gap: sexuality as culture. In: Landis, D. & Bhagat, R. (eds), *Handbook of Intercultural Training (2nd edn)*. Beverly Hills: Sage, 1995, 414-430.
38. Ross, M.W., & Ryan, L. The little deaths: perceptions of HIV, sexuality and quality of life in gay men. In: Ross, M.W. (ed.) *HIV/AIDS and Sexuality*. New York: Harrington Park Press, 1995, 1-20.
39. Ross, M.W. Societal reaction and homosexuality: acculturation, life events stressors and social supports as mediators of response to homonegative attitudes. In: Rothblum, E.D. & Bond, L.A. (eds), *Preventing Heterosexism and Homophobia*. Thousand Oaks: Sage, 1996, 205-218.

40. Brimlow, D.L. , & Ross, M.W. HIV-related communication and power in women injecting drug users. In: Roth, N.L. & Fuller, L.K. (eds), *Women and AIDS: Negotiating Safer Practices, Care and Representation*. New York: Haworth Press, 1997, 71-80.
41. Ross, M.W. Counselling per i comportamenti omosessuali a rischio. In: Bellotti, G.G. & Bellani, M.L. (eds), *Il Counselling nell'Infezione da HIV e nell'AIDS*. Milano: McGraw-Hill Libri Italia, 1997, 299-309.
42. Ross, M.W., & Brimlow, D.L. Counselling di gruppo nell'infezione da HIV. In: Bellotti, G.G. & Bellani, M.L. (eds), *Il Counselling nell'Infezione da HIV e nell'AIDS*. Milano: McGraw-Hill Libri Italia, 1997, 503-512.
43. Ross, M.W., and Heitman, E. Hoitoon sopeutuminen ja antiretroviraalinen hoito. In: Stålström, O., Lindblom, B., and Frazier, M. (eds), *AIDS-Työn Aikakauskirja* 1/97: Suomen AIDS-Tukikeskus, Helsinki, 1997, 28-34.
44. Coleman, E., Gooren, L., and Ross, M.W. Teorías sobre la transposición de género: crítica y surgencias para anhondar en la investigación. In: Nieto, J.A. (ed.), *Transexualidad, transgenerismo y cultura: Antropología, identidad y género*. Madrid, Spain: Talasa Ediciones S.L., 1998.
45. Heitman, E. and Ross, M.W. Ethical issues in the use of new treatments for HIV. In: Ostrow, D.G. & Kalichman, S. C. (eds), *Mental Health and Behavioral Issues in New HIV Therapies*. New York: Plenum Press, 1999, 113-135.
46. Ross, M.W., Wodak, A., Gold, J, and Miller, M.E. Differences in sexual orientation in relation to HIV-risk behavior among intravenous drug addicts. In: Haeberle, E.J., and Gindorf, R. (eds), *Bisexualities: The Ideology and Practice of Sexual Contact with Both Men and Women*. New York: Continuum, 1998, 234-242.
47. Ross, M.W. Psychological perspectives on sexuality and sexually transmitted diseases. In: Holmes, K.K., Mårdh, P.A., Sparling, P.F., Lemon, S.M., Stamm, W.E., Piot, P. and Wasserheit, J.N. (eds), *Sexually Transmitted Diseases (3rd edn)*. New York: McGraw-Hill, 1999, 107-113.
48. Ross, M.W. and Nilsson Schönnesson, L. HIV/AIDS and sexuality. In: Szuchman, L.T. and Muscarella, F. *Psychological Perspectives on Human Sexuality*. New York: Wiley, 2000, 383-415.
49. Ross, M.W. and Kelly, J.A. Interventions to reduce HIV transmission in homosexual men. In Peterson, J.L. and DiClemente, R.J. (eds), *Handbook of HIV Prevention*. New York: Plenum, 2000, 201-216.
50. Ross, M.W., and Paul, J.P. Beyond gender: the basis of sexual attraction in bisexual men and women. In: Rodríguez Rust, P.C. (ed.), *Bisexuality in the United States: A Social Science Reader*. New York: Columbia University Press, 2000, 92-98.
51. Griffith, K. and Ross, M.W. HIV/AIDS in the workplace. In: Diamant, L. and Lee, J.A. (eds), *The Psychology of Sex, Gender and Jobs: Issues and Solutions*. Westport: Greenwood, 2001, 185-210.
52. Ross, M.W. and Kauth, M.R. Men who have sex with men and the Internet: Emerging clinical issues and their management. In: Cooper, A. (ed.,), *Sex and the Internet: A Guidebook for Clinicians*. New York: Brunner Routledge, 2002, 47-69.
53. Williams, M., A. Klovdahl, S. Timpson, M.W. Ross, A. Bowen, and K. Keel. The social network of an HIV/STD core group of drug-using men who exchange sex for money: Preliminary results. *The Network Paradigm in Research on Drug Abuse, HIV, and other Blood-Borne and Sexually Transmitted Infections: New Perspectives, Approaches, and Applications*. Bethesda: National Institute on Drug Abuse, 2002, 15-20.
54. Williams M., A. Bowen, M.W. Ross, S. Timpson, and K. Keel. A multilevel model of condom use intention. *Strategies to Improve the Replicability, Sustainability, and Durability of HIV Prevention Interventions for Drug Users*. Bethesda: National Institute on Drug Abuse, 2003, 45-50.

55. Ross, M.W. Behavioural and counselling aspects of sexually transmitted infections. In: Kumar, B., Gupta S, Eds. *Sexually Transmitted Infections*. New Delhi: Elsevier, 2005, 75-82.
56. Ross, M.W., and Wells, A. Homosexuality, bisexuality and sexual orientation. In: Kumar, B., Gupta, S., Eds. *Sexually Transmitted Infections*. New Delhi: Elsevier, 2005, 941-945.
57. Dawson, A.G., Ross, M.W., Henry, D., and Freeman, A. Evidence of risk in "barebacking" men who have sex with men: Cases from the Internet. *Barebacking: Psychosocial and public health approaches*. New York: Haworth Press, 2006, 73-83.
58. Ross, M.W., Danawi, H., Mizwa, M.B., Cogan, L., Klein, M., Magongo, R.G., and Kganaka, M.C. Psychosocial aspects of HIV/AIDS: Adults. In: Baylor Pediatric AIDS Initiative (ed.): *HIV Curriculum for the Health Professional*. Houston: Baylor College of Medicine, 2005, 311-329.
59. Useche, B. and Ross, M.W. Public health perspectives in sexual health. In: Owens, A. and Tepper, M. (eds), *Handbook of Sexual Health*, 2007, 1-22.
60. Ross, M.W. Psychological perspectives on sexuality, STDs and HIV. In: Holmes, K.K., Mårdh, P.A., Sparling, P.F., Lemon, S.M., Stamm, W.E., Piot, P. and Wasserheit, J.N. (eds), *Sexually Transmitted Diseases (4th edn)*. New York: McGraw-Hill, 2008, 137-148.
61. Ross, M.W. Sexuality, social theory and methods, and the Internet. In: Træen, B. and Lewin, B. (eds), *Sexology in context*, Bergen: Universitetsforlaget, 2008, 295-327.
62. Daneback, K. and Ross, M.W. The complexity of Internet sexuality. In: *Sexual Dysfunction II - Beyound the Brain Body Connection*. Balon, R. (Ed.). Karger Basel, 2011.
63. Ross, M.W. and Nilsson Schönnesson, L. Behavioral and counseling aspects of sexually transmitted infections (including HIV). In: Kumar, B., Gupta S, Eds. *Sexually Transmitted Infections (2nd edn)*. New Delhi: Elsevier, 2011, 99-105.
64. Arrivillaga, M., Ross, M.W. and Varela, M.T. Socioeconomic trajectories across the life course and HIV/AIDS adherence behaviors among affected women. Capitulo por invitación. In: C. H. García, Ballester, R. & Piña, J.L. (Eds.) Chronic Diseases and Medication-Adherence Behaviors: Psychological Research in Ibero-America. New York: Nova Science Publishers, Inc., 2013. ISBN: 978-1-61470-639-7.
65. Rosser, B. R. S., Hunt, S. L., Capistrant, B. D., Kohli, N., Konety, B. R., Mitteldorf, D., Ross, M. W., Talley, K. M., & West, W. (2018). Understanding prostate cancer in gay, bisexual, and other men who have sex with men and transgender women. In J. M. Ussher, J. Perz, & B. R. S. Rosser (eds.), *Gay & bisexual men living with prostate cancer: From diagnosis to recovery* (pp. 12 – 37). New York: Harrington Park Press. doi:10.17312/harringtonparkpress/2018.06.gbmlpc.001
66. West, W., Rosser, B. R. S., Capistrant, B. D., Torres, B., Konety, B. R., Mitteldorf, D., Ross, M. W., & Talley, K. M. (2018). The effects of radiation therapy for prostate cancer on gay and bisexual men's experiences of mental health, sexual functioning and behavior, sexual identity, and relationships. In J. M. Ussher, J. Perz, & B. R. S Rosser (Eds.), *Gay & bisexual men living with prostate cancer: From diagnosis to recovery* (pp. 218-231). New York: Harrington Park Press. doi:10.17312/harringtonparkpress/2018.06.gbmlpc.013

<u>Books</u>

1. Ross, M.W. *The Married Homosexual Man: A Psychological Study*. London: Routledge and Kegan Paul, 1983.
2. Ross, M.W. (ed.) *Homosexuality and Social Sex Roles*. New York: Haworth Press, 1983.
3. Ross, M.W. (ed.) *Homosexuality, Masculinity and Femininity*. New York: Harrington Park Press, 1985.
4. Walters, W.A.W., & Ross, M.W. (eds.) *Transsexualism and Sex Reassignment*. Melbourne: Oxford University Press, 1986.

5. Ross, M.W. *Psychovenereology: Personality and Lifestyle Factors in Sexually Transmitted Diseases in Homosexual Men*. New York: Praeger, 1986.
6. Ross, M.W. (ed.) *The Treatment of Homosexuals with Mental Health Disorders*. New York: Harrington Park Press, 1988.
7. Ross, M.W. (ed.) *Psychopathology and Psychotherapy in Homosexuality*. New York: Haworth Press, 1988.
8. Ross, M.W., & Channon-Little, L.C. *Discussing Sexuality: A Guide for Health Practitioners*. Sydney: MacLennan and Petty, 1991.
   Italian translation as: Ross, M.W., & Channon-Little, L.C. *Anamnesi, Diagnosi e Counselling in Sessuologia: Disfunzioni sessuali. Malattie Trasmissibili Sessualemente*. Milano: Mediserve, 1992.
9. Bennett, L., Miller, D., & Ross, M.W. (eds.) *Health Workers and AIDS: Research, Intervention and Current Issues in Burnout and Response*. London: Harwood Academic Publishers, 1995.
10. Ross, M.W. (ed.) *HIV/AIDS and Sexuality*. New York: Harrington Park Press, 1995.
11. Lewis, L.A., & Ross, M.W. *A Select Body: The Gay Dance Party Subculture and the HIV/AIDS Pandemic*. London: Cassell, 1995.
12. Nilsson Schönnesson, L. and Ross, M.W. *Coping with HIV Infection: Psychological and existential Responses in Gay Men*. New York: Plenum Press, 1999.
13. Ross, M.W., Channon-Little, L.C., and Rosser, B.R.S. *Sexual Health Concerns: Interviewing and History Taking for Health Practitioners*. Sydney: MacLennan and Petty/ Philadelphia: FA Davis, 1999.
14. Ross, M.W. *Health and Health Promotion in Prisons (With a foreword by Lord Ramsbotham, former HM Chief Inspector of Prisons)*. London: Routledge, 2013.

Government Report

1. Coleman, E., Hyde, J.S., and Ross, M.W. (eds). *The Surgeon General's Call to Action to Promote Sexual Health and Responsible Sexual Behavior*. Washington DC: DHHS, 2001.

Appendix 5: Declaration of Confidentiality

DECLARATION OF CONFIDENTIALITY AND DATA PRIVACY

(to be signed by all persons involved in the 1st Global LGBT Foundation and UNAIDS Survey on Happiness, Sex and Quality of life)

I will be bound by all the terms and conditions of the confidentiality undertaking signed by the duly designated representative of my research entity and will use the dataset indicated in the research proposal in accordance with the terms of use attached to the confidentiality undertaking.

I understand that I must treat all data related to the LGBTI Global Survey in accordance with the EU Directive 95/46/EC and as amended, replaced or superseded from time to time, including by the EU General Data Protection Regulation 2016/679 (GDPR)

I will:
(a) use the dataset only for the purposes specified in the research proposal;
(b) safeguard the dataset and any usernames and passwords associated with it;
(c) ensure that any results of analyses will not be disclosive or potentially disclosive in conjunction with other publicly available information;
(d) acknowledge the dataset and its source in any research report or publication and also state that the results and conclusions are mine and not those of the LGBT Foundation, UNAIDS, Aix-Marseille University or University of Minnesota;
(e) provide the LGBT Foundation and UNAIDS with references to publications and other research reports based on this dataset;
(f) preserve the confidentiality of information pertaining to identifiable individuals, households and/or organisations, such as those using the email address provided in the survey to exercise their right of withdrawal;
(g) submit the final complete output of my work for the confidentiality check to the competent LGBT Foundation or UNAIDS staff (in case of access to secure use files);
(h) destroy the dataset and any data or variables derived from it at the end of the research period specified in the research proposal and sign a declaration to the effect that it has been ensured that all data have been destroyed;
(i) abide by any other conditions notified to me by LGBT Foundation and UNAIDS (e.g. guidelines for publication);
(j) inform the LGBT Foundation and UNAIDS immediately about any breach of the confidentiality rules laid down in the confidentiality undertaking or in the terms of use of confidential data for scientific purposes.

I will not:
(a) use the data (scientific use files) outside the premises of my research entity;
(b) allow non-authorised users to access the dataset (authorized users are named in the research proposal);
(c) use the data for research purposes before it is checked for confidentiality by the LGBT Foundation and UNAIDS (in case of access to secure use files)
(d) remove the data or any part of it (in case of access to secure use files);
(e) attempt to link the data to other (including public) datasets, whether or not provided by the LGBT Foundation and UNAIDS, if not expressly agreed;
(f) attempt to identify any individual record (individual, household, business, etc.) in the dataset, or claim to have done so;

(g) release or publish any information or results which identify any individual record or may lead to the identification of any individual record.

I certify that I have read all of the above clauses, that I understand that I am accountable for correct and responsible use of the data and data access system, and that I understand that if I fail to comply with these clauses, my access to the dataset will be withdrawn and I will be liable to any other sanctions that may be determined by my research entity or are specified in the applicable civil or penal law.

Name: …………………………………Signature: ………………………..Date: ……………………

References

[1] Hatzenbuehler ML, Phelan JC, Link BG. Stigma as a fundamental cause of population health inequalities. Am J Public Health 2013; 103:813-21.
[2] Ha H, Risser JM, Ross MW, Huynh NT, Nguyen HT. Homosexuality-related stigma and sexual risk behaviors among men who have sex with men in Hanoi, Vietnam. Arch Sex Behav 2015; 44:349-56.
[3] Jeffries WLt, Marks G, Lauby J, Murrill CS, Millett GA. Homophobia is associated with sexual behavior that increases risk of acquiring and transmitting HIV infection among black men who have sex with men. AIDS and Behavior 2013; 17:1442-53.
[4] Arreola S, Santos GM, Beck J, Sundararaj M, Wilson PA, Hebert P, Makofane K, Do TD, Ayala G. Sexual stigma, criminalization, investment, and access to HIV services among men who have sex with men worldwide. AIDS and Behavior 2015; 19:227-34.
[5] Lorenc T, Marrero-Guillamon I, Llewellyn A, Aggleton P, Cooper C, Lehmann A, Lindsay C. HIV testing among men who have sex with men (MSM): systematic review of qualitative evidence. Health Educ Res, 2011:834-46.
[6] Adebajo SB, Eluwa GI, Allman D, Myers T, Ahonsi BA. Prevalence of internalized homophobia and HIV associated risks among men who have sex with men in Nigeria. Afr J Reprod Health 2012; 16:21-8.
[7] Wei C, Yan H, Yang C, Raymond HF, Li J, Yang H, Zhao J, Huan X, Stall R. Accessing HIV testing and treatment among men who have sex with men in China: a qualitative study. AIDS Care 2013; 26.
[8] Risher K, Adams D, Sithole B, Ketende S, Kennedy C, Mnisi Z, Mabusa X, Baral SD. Sexual stigma and discrimination as barriers to seeking appropriate healthcare among men who have sex with men in Swaziland. J Int AIDS Soc 2013; 16.
[9] Andrinopoulos K, Hembling J, Guardado ME, de Maria Hernandez F, Nieto AI, Melendez G. Evidence of the negative effect of sexual minority stigma on HIV testing among MSM and transgender women in San Salvador, El Salvador. AIDS and Behavior 2015; 19:60-71.
[10] Gu J, Lau JT, Wang Z, Wu AM, Tan X. Perceived empathy of service providers mediates the association between perceived discrimination and behavioral intention to take up HIV antibody testing again among men who have sex with men. PLoS One 2015; 10.
[11] Ross MW, Berg RC, Schmidt AJ, Hospers HJ, Breveglieri M, Furegato M, Weatherburn P. Internalised homonegativity predicts HIV-associated risk behavior in European men who have sex with men in a 38-country cross-sectional study: Some public health implications of homophobia. BMJ Open 2013b; 3.
[12] McLeroy KR, Bibeau D, Steckler A, Glanz K. An ecological perspective on health promotion programs. Health education quarterly 1988; 15:351-77.
[13] Link BG, Phelan JC. Conceptualizing stigma. Annu Rev Sociol 2001; 27:363-85.
[14] Hatzenbuehler ML. Structural stigma and the health of lesbian, gay, and bisexual populations. Current Directions in Psychological Science 2014c; 23:127-32.
[15] Hatzenbuehler ML, Bränström R, Pachankis JE. Societal-level explanations for reductions in sexual orientation mental health disparities; Results from a ten-year, population-based study in Sweden. Stigma and Health, 2016.
[16] Stangl AL, Lloyd JK, Brady LM, Holland CE, Baral S. A systematic review of interventions to reduce HIV-related stigma and discrimination from 2002 to 2013: how far have we come? , 2013.
[17] White Hughto JM, Reisner SL, Pachankis JE. Transgender stigma and health: A critical review of stigma determinants, mechanisms, and interventions. Social Science & Medicine 2015; 147:222-31.
[18] Baral SD, Poteat T, Strömdahl S, Wirtz AL, Guadamuz TE, Beyrer C. Worldwide burden of HIV in transgender women: a systematic review and meta-analysis. The Lancet Infectious Diseases 2013; 13:214-22.
[19] Lamontagne E, d'Elbée M, Ross MW, Carroll A, Plessis Ad, Loures L. A socioecological measurement of homophobia for all countries and its public health impact. European Journal of Public Health 2018; 28:967-72.
[20] UNAIDS. Miles to go: Closing gaps, breaking barrier, righting injustices. 2018.
[21] Pachankis JE, Hatzenbuehler ML, Hickson F, Weatherburn P, Berg RC, Marcus U, Schmidt AJ. Hidden from health: structural stigma, sexual orientation concealment, and HIV across 38 countries in the European MSM Internet Survey. AIDS 2015; 29:1239-46.
