# Supplementary material for: Assessment and determinants of depression and anxiety on a global sample of sexual and gender diverse people at high risk of HIV: a public health approach
Source: BMC Public Health. 2024 Jan 18;24:215. doi: 10.1186/s12889-023-17493-8 (PMC10795213; doi:10.1186/s12889-023-17493-8)
Supplement: Supplementary file 2 — Additional file 2: Supplement S1. Final sample per country and per sexual and gender identity. S2. Parallel regression assumption. Supplement S3. Tests for the exclusion of participants who did not inform their HIV status from the multinomial regressions. Supplement S4. Multinomial logistic regressions per sexual and gender-diverse categories. [file 12889_2023_17493_MOESM2_ESM.pdf]

Supplement S1: Final sample per country and per sexual and gender identity

Country	*Total*		*Gay men*		Bisexual		Transfeminine		Queer or Questioning	
	N	%	N	%	N	%	N	%	N	%
Afghanistan	67	0.06%	16	0.02%	27	0.18%	6	0.13%	18	0.13%
Albania	24	0.02%	13	0.02%	3	0.02%	1	0.02%	7	0.05%
Algeria	376	0.35%	167	0.22%	93	0.63%	18	0.40%	98	0.68%
Andorra	40	0.04%	30	0.04%	3	0.02%	1	0.02%	6	0.04%
Angola	174	0.16%	81	0.11%	28	0.19%	20	0.45%	45	0.31%
Antigua and Barbuda	19	0.02%	10	0.01%	4	0.03%			5	0.03%
Argentina	329	0.30%	255	0.34%	39	0.26%	4	0.09%	31	0.22%
Armenia	122	0.11%	75	0.10%	18	0.12%	5	0.11%	24	0.17%
Aruba	23	0.02%	15	0.02%	2	0.01%	1	0.02%	5	0.03%
Australia	717	0.66%	596	0.80%	65	0.44%	24	0.54%	32	0.22%
Austria	53	0.05%	41	0.05%	5	0.03%	3	0.07%	4	0.03%
Azerbaijan	178	0.16%	110	0.15%	26	0.17%	4	0.09%	38	0.27%
Bahamas, The	62	0.06%	36	0.05%	15	0.10%	3	0.07%	8	0.06%
Bahrain	127	0.12%	53	0.07%	34	0.23%	5	0.11%	35	0.24%
Bangladesh	108	0.10%	60	0.08%	20	0.13%	10	0.22%	18	0.13%
Barbados	6	0.01%	4	0.01%	2	0.01%				
Belarus	841	0.78%	638	0.85%	84	0.56%	18	0.40%	101	0.71%
Belgium	421	0.39%	316	0.42%	45	0.30%	20	0.45%	40	0.28%
Belize	33	0.03%	21	0.03%	3	0.02%	3	0.07%	6	0.04%
Benin	181	0.17%	67	0.09%	35	0.24%	35	0.78%	44	0.31%
Bhutan	38	0.04%	15	0.02%	10	0.07%	4	0.09%	9	0.06%
Bolivia	122	0.11%	76	0.10%	28	0.19%	1	0.02%	17	0.12%
Bosnia and Herzegovina	8	0.01%	2	0.00%	3	0.02%	1	0.02%	2	0.01%
Botswana	26	0.02%	17	0.02%	7	0.05%	1	0.02%	1	0.01%
Brazil	13066	12.05%	10434	13.96%	1491	10.03%	83	1.86%	1058	7.39%

Country	*Total*		*Gay men*		Bisexual		Transfeminine		Queer or Questioning	
	N	%	N	%	N	%	N	%	N	%
Brunei	12	0.01%	9	0.01%	2	0.01%			1	0.01%
Bulgaria	77	0.07%	61	0.08%	7	0.05%	1	0.02%	8	0.06%
Burkina Faso	171	0.16%	56	0.07%	44	0.30%	32	0.72%	39	0.27%
Burma	146	0.13%	101	0.14%	13	0.09%	13	0.29%	19	0.13%
Burundi	16	0.01%	12	0.02%	1	0.01%	1	0.02%	2	0.01%
Cabo Verde	3	0.00%	2	0.00%					1	0.01%
Cambodia	117	0.11%	65	0.09%	15	0.10%	5	0.11%	32	0.22%
Cameroon	125	0.12%	56	0.07%	27	0.18%	15	0.34%	27	0.19%
Canada	960	0.89%	791	1.06%	94	0.63%	20	0.45%	55	0.38%
Central African Republic	36	0.03%	19	0.03%	7	0.05%	2	0.04%	8	0.06%
Chad	42	0.04%	16	0.02%	12	0.08%	1	0.02%	13	0.09%
Chile	427	0.39%	339	0.45%	35	0.24%	7	0.16%	46	0.32%
China	7000	6.46%	4514	6.04%	1088	7.32%	585	13.11%	813	5.67%
Colombia	647	0.60%	465	0.62%	71	0.48%	22	0.49%	89	0.62%
Costa Rica	483	0.45%	396	0.53%	49	0.33%	5	0.11%	33	0.23%
Cote d'Ivoire	187	0.17%	86	0.12%	39	0.26%	18	0.40%	44	0.31%
Croatia	89	0.08%	60	0.08%	14	0.09%	2	0.04%	13	0.09%
Cuba	43	0.04%	36	0.05%	2	0.01%	2	0.04%	3	0.02%
Curacao	1	0.00%	1	0.00%						
Cyprus	178	0.16%	114	0.15%	33	0.22%	5	0.11%	26	0.18%
Czechia	235	0.22%	172	0.23%	24	0.16%	18	0.40%	21	0.15%
Democratic Republic of the Congo	329	0.30%	57	0.08%	41	0.28%	133	2.98%	98	0.68%
Denmark	134	0.12%	115	0.15%	13	0.09%	3	0.07%	3	0.02%
Djibouti	7	0.01%	3	0.00%	1	0.01%			3	0.02%
Dominica	3	0.00%	2	0.00%	1	0.01%				
Dominican Republic	204	0.19%	137	0.18%	25	0.17%	10	0.22%	32	0.22%

Country	*Total*		*Gay men*		Bisexual		Transfeminine		Queer or Questioning	
	N	%	N	%	N	%	N	%	N	%
Ecuador	209	0.19%	130	0.17%	28	0.19%	22	0.49%	29	0.20%
Egypt	626	0.58%	372	0.50%	102	0.69%	44	0.99%	108	0.75%
El Salvador	149	0.14%	101	0.14%	15	0.10%	9	0.20%	24	0.17%
Equatorial Guinea	1	0.00%							1	0.01%
Eritrea	1	0.00%	1	0.00%						
Estonia	113	0.10%	80	0.11%	17	0.11%	8	0.18%	8	0.06%
Eswatini	64	0.06%	35	0.05%	6	0.04%	11	0.25%	12	0.08%
Ethiopia	141	0.13%	81	0.11%	27	0.18%	10	0.22%	23	0.16%
Fiji	107	0.10%	58	0.08%	8	0.05%	15	0.34%	26	0.18%
Finland	85	0.08%	59	0.08%	12	0.08%	5	0.11%	9	0.06%
France	6737	6.22%	5787	7.74%	598	4.02%	29	0.65%	323	2.25%
Gabon	107	0.10%	54	0.07%	34	0.23%	4	0.09%	15	0.10%
Gambia, The	2	0.00%	1	0.00%	1	0.01%				
Georgia	296	0.27%	185	0.25%	51	0.34%	12	0.27%	48	0.34%
Germany	501	0.46%	396	0.53%	45	0.30%	8	0.18%	52	0.36%
Ghana	140	0.13%	84	0.11%	24	0.16%	15	0.34%	17	0.12%
Greece	115	0.11%	77	0.10%	16	0.11%	9	0.20%	13	0.09%
Grenada	2	0.00%	2	0.00%						
Guatemala	167	0.15%	125	0.17%	23	0.15%	5	0.11%	14	0.10%
Guinea	7	0.01%	4	0.01%	1	0.01%			2	0.01%
Guinea-Bissau	1	0.00%	1	0.00%						
Guyana	108	0.10%	67	0.09%	21	0.14%	9	0.20%	11	0.08%
Haiti	209	0.19%	96	0.13%	47	0.32%	22	0.49%	44	0.31%
Holy See	7	0.01%	4	0.01%	1	0.01%	1	0.02%	1	0.01%
Honduras	146	0.13%	84	0.11%	25	0.17%	10	0.22%	27	0.19%
Hong Kong	262	0.24%	205	0.27%	26	0.17%	2	0.04%	29	0.20%

Country	*Total*		*Gay men*		Bisexual		Transfeminine		Queer or Questioning	
	N	%	N	%	N	%	N	%	N	%
Hungary	52	0.05%	40	0.05%	3	0.02%	2	0.04%	7	0.05%
Iceland	25	0.02%	14	0.02%	6	0.04%	3	0.07%	2	0.01%
India	6294	5.81%	2828	3.78%	1241	8.34%	501	11.23%	1724	12.03%
Indonesia	2926	2.70%	1781	2.38%	700	4.71%	122	2.73%	323	2.25%
Iran	1748	1.61%	1082	1.45%	286	1.92%	106	2.38%	274	1.91%
Iraq	171	0.16%	115	0.15%	21	0.14%	8	0.18%	27	0.19%
Ireland	141	0.13%	100	0.13%	17	0.11%	4	0.09%	20	0.14%
Israel	131	0.12%	100	0.13%	15	0.10%			16	0.11%
Italy	413	0.38%	352	0.47%	36	0.24%	5	0.11%	20	0.14%
Jamaica	95	0.09%	51	0.07%	17	0.11%	6	0.13%	21	0.15%
Japan	509	0.47%	417	0.56%	43	0.29%	10	0.22%	39	0.27%
Jordan	209	0.19%	128	0.17%	37	0.25%	11	0.25%	33	0.23%
Kazakhstan	621	0.57%	400	0.54%	94	0.63%	28	0.63%	99	0.69%
Kenya	177	0.16%	110	0.15%	34	0.23%	17	0.38%	16	0.11%
Kiribati	6	0.01%	4	0.01%			2	0.04%		
Korea, North	4	0.00%	4	0.01%						
Kosovo	14	0.01%	5	0.01%	4	0.03%	1	0.02%	4	0.03%
Kuwait	112	0.10%	64	0.09%	21	0.14%	8	0.18%	19	0.13%
Kyrgyzstan	166	0.15%	112	0.15%	27	0.18%	5	0.11%	22	0.15%
Laos	152	0.14%	81	0.11%	19	0.13%	9	0.20%	43	0.30%
Latvia	81	0.07%	66	0.09%	6	0.04%	1	0.02%	8	0.06%
Lebanon	206	0.19%	147	0.20%	18	0.12%	5	0.11%	36	0.25%
Lesotho	51	0.05%	29	0.04%	4	0.03%	11	0.25%	7	0.05%
Liberia	34	0.03%	15	0.02%	11	0.07%	5	0.11%	3	0.02%
Libya	223	0.21%	98	0.13%	49	0.33%	17	0.38%	59	0.41%
Liechtenstein	2	0.00%	1	0.00%					1	0.01%

Country	*Total*		*Gay men*		Bisexual		Transfeminine		Queer or Questioning	
	N	%	N	%	N	%	N	%	N	%
Lithuania	101	0.09%	76	0.10%	10	0.07%	5	0.11%	10	0.07%
Luxembourg	21	0.02%	20	0.03%					1	0.01%
Macau	19	0.02%	15	0.02%	4	0.03%				
Macedonia	123	0.11%	62	0.08%	25	0.17%	5	0.11%	31	0.22%
Madagascar	5	0.00%	4	0.01%	1	0.01%				
Malawi	49	0.05%	18	0.02%	15	0.10%	7	0.16%	9	0.06%
Malaysia	836	0.77%	558	0.75%	110	0.74%	55	1.23%	113	0.79%
Maldives	7	0.01%	5	0.01%	1	0.01%			1	0.01%
Mali	146	0.13%	66	0.09%	27	0.18%	21	0.47%	32	0.22%
Malta	48	0.04%	36	0.05%	6	0.04%	3	0.07%	3	0.02%
Marshall Islands	17	0.02%	8	0.01%	1	0.01%	5	0.11%	3	0.02%
Mauritania	114	0.11%	51	0.07%	15	0.10%	13	0.29%	35	0.24%
Mauritius	144	0.13%	75	0.10%	30	0.20%	11	0.25%	28	0.20%
Mexico	6306	5.82%	4867	6.51%	709	4.77%	68	1.52%	662	4.62%
Micronesia	8	0.01%	3	0.00%	3	0.02%	1	0.02%	1	0.01%
Moldova	140	0.13%	95	0.13%	16	0.11%	8	0.18%	21	0.15%
Monaco	1	0.00%							1	0.01%
Mongolia	2	0.00%	2	0.00%						
Montenegro	12	0.01%	8	0.01%	1	0.01%	1	0.02%	2	0.01%
Morocco	333	0.31%	199	0.27%	41	0.28%	7	0.16%	86	0.60%
Mozambique	246	0.23%	95	0.13%	41	0.28%	36	0.81%	74	0.52%
Namibia	113	0.10%	63	0.08%	10	0.07%	25	0.56%	15	0.10%
Nauru	7	0.01%	3	0.00%			3	0.07%	1	0.01%
Nepal	126	0.12%	57	0.08%	14	0.09%	19	0.43%	36	0.25%
Netherlands	349	0.32%	272	0.36%	37	0.25%	5	0.11%	35	0.24%
New Zealand	192	0.18%	154	0.21%	25	0.17%	3	0.07%	10	0.07%

Country	*Total*		*Gay men*		Bisexual		Transfeminine		Queer or Questioning	
	N	%	N	%	N	%	N	%	N	%
Nicaragua	24	0.02%	14	0.02%	3	0.02%	2	0.04%	5	0.03%
Niger	12	0.01%	8	0.01%			1	0.02%	3	0.02%
Nigeria	277	0.26%	170	0.23%	51	0.34%	24	0.54%	32	0.22%
Norway	97	0.09%	73	0.10%	8	0.05%	4	0.09%	12	0.08%
Oman	57	0.05%	21	0.03%	17	0.11%	7	0.16%	12	0.08%
Pakistan	186	0.17%	111	0.15%	35	0.24%	11	0.25%	29	0.20%
Palau	6	0.01%	4	0.01%	1	0.01%	1	0.02%		
Palestinian Territories	27	0.02%	12	0.02%	5	0.03%	1	0.02%	9	0.06%
Panama	151	0.14%	109	0.15%	19	0.13%	3	0.07%	20	0.14%
Papua New Guinea	35	0.03%	13	0.02%	6	0.04%	9	0.20%	7	0.05%
Paraguay	89	0.08%	66	0.09%	9	0.06%	4	0.09%	10	0.07%
Peru	284	0.26%	211	0.28%	35	0.24%	8	0.18%	30	0.21%
Philippines	310	0.29%	167	0.22%	49	0.33%	17	0.38%	77	0.54%
Poland	254	0.23%	169	0.23%	36	0.24%	10	0.22%	39	0.27%
Portugal	196	0.18%	163	0.22%	20	0.13%	3	0.07%	10	0.07%
Qatar	66	0.06%	35	0.05%	12	0.08%	2	0.04%	17	0.12%
Republic of the Congo	171	0.16%	96	0.13%	31	0.21%	15	0.34%	29	0.20%
Romania	76	0.07%	51	0.07%	14	0.09%	6	0.13%	5	0.03%
Russia	12250	11.30%	9300	12.44%	1255	8.44%	303	6.79%	1392	9.72%
Rwanda	168	0.15%	78	0.10%	29	0.19%	39	0.87%	22	0.15%
Saint Kitts and Nevis	1	0.00%			1	0.01%				
Saint Lucia	8	0.01%	6	0.01%	2	0.01%				
Saint Vincent and the Grenadines	3	0.00%	2	0.00%	1	0.01%				
Samoa	23	0.02%	7	0.01%	2	0.01%	6	0.13%	8	0.06%
San Marino	2	0.00%	1	0.00%					1	0.01%
Saudi Arabia	1595	1.47%	854	1.14%	360	2.42%	71	1.59%	310	2.16%

Country	*Total*		*Gay men*		Bisexual		Transfeminine		Queer or Questioning	
	N	%	N	%	N	%	N	%	N	%
Senegal	57	0.05%	19	0.03%	25	0.17%	6	0.13%	7	0.05%
Serbia	129	0.12%	83	0.11%	24	0.16%	8	0.18%	14	0.10%
Seychelles	3	0.00%	3	0.00%						
Sierra Leone	6	0.01%	2	0.00%	1	0.01%	1	0.02%	2	0.01%
Singapore	178	0.16%	145	0.19%	18	0.12%	2	0.04%	13	0.09%
Sint Maarten	8	0.01%	8	0.01%						
Slovakia	15	0.01%	12	0.02%					3	0.02%
Slovenia	79	0.07%	59	0.08%	10	0.07%	2	0.04%	8	0.06%
Solomon Islands	4	0.00%	1	0.00%			2	0.04%	1	0.01%
Somalia	2	0.00%	1	0.00%					1	0.01%
South Africa	768	0.71%	519	0.69%	94	0.63%	36	0.81%	119	0.83%
South Korea	252	0.23%	168	0.22%	35	0.24%	9	0.20%	40	0.28%
South Sudan	2	0.00%	1	0.00%					1	0.01%
Spain	635	0.59%	522	0.70%	69	0.46%	8	0.18%	36	0.25%
Sri Lanka	106	0.10%	64	0.09%	16	0.11%	5	0.11%	21	0.15%
Sudan	26	0.02%	17	0.02%	4	0.03%	1	0.02%	4	0.03%
Suriname	117	0.11%	65	0.09%	32	0.22%	4	0.09%	16	0.11%
Sweden	167	0.15%	125	0.17%	20	0.13%	7	0.16%	15	0.10%
Switzerland	270	0.25%	236	0.32%	18	0.12%	2	0.04%	14	0.10%
Syria	150	0.14%	89	0.12%	21	0.14%	11	0.25%	29	0.20%
Taiwan	2287	2.11%	1718	2.30%	267	1.80%	87	1.95%	215	1.50%
Tajikistan	33	0.03%	10	0.01%	10	0.07%	3	0.07%	10	0.07%
Tanzania	191	0.18%	84	0.11%	31	0.21%	27	0.61%	49	0.34%
Thailand	4817	4.44%	3011	4.03%	475	3.19%	72	1.61%	1259	8.79%
Timor-Leste	11	0.01%	6	0.01%	1	0.01%	3	0.07%	1	0.01%
Togo	22	0.02%	11	0.01%	2	0.01%	1	0.02%	8	0.06%

Country	*Total*		*Gay men*		Bisexual		Transfeminine		Queer or Questioning	
	N	%	N	%	N	%	N	%	N	%
Tonga	20	0.02%	9	0.01%	1	0.01%	8	0.18%	2	0.01%
Trinidad and Tobago	14	0.01%	8	0.01%	3	0.02%			3	0.02%
Tunisia	173	0.16%	105	0.14%	22	0.15%	7	0.16%	39	0.27%
Turkey	8157	7.53%	4639	6.21%	1591	10.70%	597	13.38%	1330	9.28%
Turkmenistan	9	0.01%	6	0.01%	1	0.01%			2	0.01%
Tuvalu	7	0.01%	5	0.01%	1	0.01%			1	0.01%
Uganda	124	0.11%	63	0.08%	24	0.16%	17	0.38%	20	0.14%
Ukraine	3395	3.13%	2521	3.37%	452	3.04%	70	1.57%	352	2.46%
United Arab Emirates	440	0.41%	268	0.36%	78	0.52%	23	0.52%	71	0.50%
United Kingdom	1626	1.50%	1356	1.81%	156	1.05%	34	0.76%	80	0.56%
United States of America	2706	2.50%	1955	2.62%	400	2.69%	149	3.34%	202	1.41%
Uruguay	96	0.09%	61	0.08%	17	0.11%	7	0.16%	11	0.08%
Uzbekistan	183	0.17%	117	0.16%	33	0.22%	5	0.11%	28	0.20%
Vanuatu	9	0.01%	4	0.01%			3	0.07%	2	0.01%
Venezuela	150	0.14%	116	0.16%	13	0.09%	7	0.16%	14	0.10%
Vietnam	442	0.41%	282	0.38%	29	0.19%	16	0.36%	115	0.80%
Yemen	59	0.05%	35	0.05%	9	0.06%	3	0.07%	12	0.08%
Zambia	107	0.10%	66	0.09%	17	0.11%	9	0.20%	15	0.10%
Zimbabwe	97	0.09%	75	0.10%	8	0.05%	5	0.11%	9	0.06%
Missing	708	0.65%	441	0.59%	116	0.78%	48	1.08%	103	0.72%
Total	108389	100%	74730	100%	14872	100%	4461	100%	14326	100%

S2: Parallel regression assumption

We performed the Brant test of parallel regression assumption for the model. Table S2 below shows that the parallel assumption is violated for most variables, apart from the measures of HIV status as well as stigma and discrimination in healthcare centres. The parallel assumption is a key requirement before performing an ordinal logistic regression. Therefore, we opted for a multinomial logistic regression model as it is generally considered an extension of ordinal logistic model.

Table S2: Brant test

Variables	chi2	p>chi2
**All**	**583.72**	**0.000**
1.AgeCat (young (18-24)	4.53	0.104
3.AgeCat (older adult, 35+)	12.02	0.002
1.EducationCat (No education/primary)	4.43	0.109
2.EducationCat (Secondary education)	9.10	0.011
1.SES (Struggling on present income)	18.27	0.000
3.SES (Living comfortably on present income)	34.06	0.000
2.HIV status (positive)	1.10	0.577
3.HIV status (I don't know)	6.44	0.040
4.HIV status (I don't want to answer)	1.17	0.556
Family support	51.46	0.000
Social support network from friend	24.43	0.000
Homophobic reactions	11.73	0.003
Homophobia workplace	21.49	0.000
S&D healthcare	5.67	0.059
Asia and Pacific dummy	55.41	0.000
Easter Europe and Central Asia dummy	16.24	0.000
Sub-Saharan Africa dummy	7.37	0.025
Latin America and Caribbean dummy	154.77	0.000
Middle East and North Africa dummy	21.63	0.000

Supplement S3: Tests for the exclusion of participants who did not inform their HIV status from the multinomial regressions

The survey enabled participants to skip any question they felt uncomfortable with, or did not want to answer. Missing responses are encoded accordingly in the dataset. The study considered those participants who informed all the variables in this study. We did not impute missing variables.

Considering the number of participants who preferred not to inform their HIV status, we tested below whether this selection could create a systematic bias on the dependent variables (PHQ-4).

We compare here the participants who informed and those who did not inform their HIV status for each score of the dependent variable (PHQ-4).

Table S3.1 Number of participants who informed their HIV status, per severity of symptoms of anxiety and depression.

Symptoms of anxiety and depression	FOR INDIVIDUALS WITH COMPLETE QUESTIONNAIRES		
	Freq.	Percent	Cum.
None	33,153	29.69	29.69
Mild	43,988	39.39	69.08
Moderate	19,808	17.74	86.82
Severe	14,715	13.18	100.00
Total	111,664	100.00	

Table S3.2 Number of participants who did not inform their HIV status, per severity of symptoms of anxiety and depression.

Symptoms of anxiety and depression	FOR INDIVIDUALS WITH MISSING RESPONSE ON HIV STATUS		
	Freq.	Percent	Cum.
None	6,158	31.65	31.65
Mild	7,402	38.04	69.69
Moderate	3,428	17.62	87.31
Severe	2,470	12.69	100.00
Total	19,458	100.00	

Table S3.2 describes the severity score of anxiety and depression symptoms of those participants who did not inform their HIV status. For example, 6 158 is the number of respondents that answered the PHQ-4 that have no symptoms of anxiety and depression and did not answer the HIV status question.

It appears that the patterns of respondents are comparable between the two samples (29.69 – 31.65 / 39.39 – 38.04 / 17.74 – 17.62 / 13.18 – 12.69). Therefore, there is no evidence of a lack of representativeness induced by missing data.

Supplement S4: Multinomial logistic regressions per sexual and gender-diverse categories

Table S4.1: Gay cisgender men and intersex

Gay cisgender men and intersex	Mild symptoms				Moderate symptoms				Severe symptoms			
	RRR	p-value	95% Conf. Interval		RRR	p-value	95% Conf. Interval		RRR	p-value	95% Conf. Interval	
**Age group:**												
**Young (18-24)**	1.142	0.000	1.08	1.207	1.298	0.000	1.214	1.388	1.33	0.000	1.234	1.433
**Adult (25-34)**	(base)				(base)				(base)			
**Older adults (35+)**	0.697	0.000	0.665	0.73	0.55	0.000	0.517	0.586	0.531	0.000	0.494	0.572
**Education**												
**No education/primary**	1.004	0.957	0.866	1.164	1.132	0.175	0.946	1.353	1.233	0.035	1.015	1.497
**Secondary education**	1.061	0.021	1.009	1.117	1.202	0.000	1.129	1.279	1.276	0.000	1.191	1.367
**Higher education**	(base)				(base)				(base)			
**Economic vulnerability**												
**Struggling on present income**	1.43	0.000	1.347	1.519	2.026	0.000	1.891	2.171	3.184	0.000	2.956	3.43
**Neither comfortable on present income**	(base)				(base)				(base)			
**Living comfortably on present income**	0.616	0.000	0.589	0.644	0.521	0.000	0.49	0.555	0.48	0.000	0.444	0.519
**HIV status**												
**HIV-negative**	(base)				(base)				(base)			
**HIV-positive**	1.022	0.497	0.96	1.087	1.12	0.006	1.033	1.214	1.117	0.018	1.019	1.225
**I don't know**	1.178	0.000	1.114	1.246	1.25	0.000	1.167	1.339	1.278	0.000	1.184	1.379
**I don't want to answer**	0.91	0.089	0.817	1.015	0.999	0.993	0.87	1.148	0.934	0.404	0.795	1.096
**Social support network**												
**Social support: family**	0.826	0.000	0.811	0.842	0.762	0.000	0.745	0.78	0.745	0.000	0.726	0.764
**Social support: friends**	0.755	0.000	0.738	0.772	0.636	0.000	0.619	0.653	0.538	0.000	0.523	0.553
**Stigma and discrimination**												
**Homophobic reactions**	1.502	0.000	1.436	1.571	1.87	0.000	1.758	1.99	2.206	0.000	2.045	2.38
**Homophobia at the work place**	1.427	0.000	1.33	1.53	1.719	0.000	1.586	1.863	1.866	0.000	1.712	2.034
**Avoiding healthcare**	1.034	0.548	0.927	1.154	1.221	0.002	1.077	1.384	1.477	0.000	1.296	1.684
**Continent dummies**												
**Asia**	0.608	0.000	0.572	0.645	0.497	0.000	0.459	0.539	0.459	0.000	0.418	0.50449
**Eastern Europe**	0.985	0.626	0.925	1.048	0.825	0.000	0.762	0.894	0.899	0.022	0.821	0.985
**Sub-Saharan Africa**	0.718	0.000	0.636	0.811	0.564	0.000	0.486	0.655	0.462	0.000	0.390	0.548
**Latin America**	0.659	0.000	0.623	0.697	0.64	0.000	0.595	0.688	0.765	0.000	0.704	0.831

**Middle east**	0.902	0.107	0.796	1.022	1.159	0.038	1.008	1.333	1.541	0.000	1.332	1.782
**Constant**	9.333	0.000	8.262	10.542	8.124	0.000	7.028	9.391	7.463	0.000	6.361	8.755
**N**	60.162											
**Log likelihood**	-74186.9											
**Likelihood Ratio**	chi2(39) = 14940.62											
**Prob > chi2**	0.0000											

Table S4.2 Bisexual cisgender men and intersex

Bisexual cisgender men and intersex	Mild symptoms				Moderate symptoms				Severe symptoms			
	RRR	p-value	95% Conf. Interval		RRR	p-value	95% Conf. Interval		RRR	p-value	95% Conf. Interval	
**Age group:**												
**Young (18-24)**	1.28	0.000	1.14	1.437	1.568	0.000	1.361	1.806	1.638	0.000	1.393	1.926
**Adult (25-34)**	(base)				(base)				(base)			
**Older adults (35+)**	0.726	0.000	0.647	0.813	0.619	0.000	0.531	0.723	0.568	0.000	0.471	0.684
**Education**												
**No education/primary**	0.937	0.649	0.709	1.239	1.314	0.097	0.952	1.815	1.284	0.181	0.891	1.851
**Secondary education**	1.093	0.105	0.982	1.216	1.227	0.002	1.075	1.4	1.242	0.005	1.067	1.445
**Higher education**	(base)				(base)				(base)			
**Economic vulnerability**												
**Struggling on present income**	1.52	0.000	1.335	1.731	1.981	0.000	1.703	2.305	3.135	0.000	2.647	3.712
**Neither comfortable on present income**	(base)				(base)				(base)			
**Living comfortably on present income**	0.657	0.000	0.591	0.73	0.527	0.000	0.456	0.609	0.53	0.000	0.44	0.637
**HIV status**												
**HIV-negative**	(base)				(base)				(base)			
**HIV-positive**	1.207	0.053	0.997	1.461	1.384	0.007	1.091	1.756	1.295	0.071	0.978	1.716
**I don't know**	1.186	0.004	1.057	1.331	1.361	0.000	1.183	1.567	1.429	0.000	1.218	1.677
**I don't want to answer**	1.018	0.877	0.817	1.268	0.996	0.975	0.749	1.324	0.67	0.037	0.461	0.975
**Social support network**												
**Social support: family**	0.863	0.000	0.829	0.9	0.816	0.000	0.775	0.858	0.705	0.000	0.665	0.747
**Social support: friends**	0.806	0.000	0.769	0.844	0.699	0.000	0.662	0.739	0.623	0.000	0.586	0.662
**Stigma and discrimination**												
**Homophobic reactions**	1.69	0.000	1.531	1.865	2.117	0.000	1.856	2.414	2.456	0.000	2.092	2.883
**Homophobia at the work place**	1.495	0.000	1.26	1.775	1.548	0.000	1.268	1.889	1.828	0.000	1.475	2.265
**Avoiding healthcare**	1.001	0.991	0.799	1.255	1.287	0.054	0.995	1.664	1.355	0.033	1.025	1.791
**Continent dummies**												
**Asia**	0.594	0.000	0.519	0.678	0.616	0.000	0.519	0.731	0.599	0.000	0.489	0.733
**Eastern Europe**	0.85	0.034	0.732	0.988	0.714	0.001	0.584	0.872	0.839	0.136	0.666	1.057
**Sub-Saharan Africa**	0.923	0.487	0.738	1.156	0.728	0.025	0.552	0.96	0.604	0.002	0.44	0.83

**Latin America**	0.622	0.000	0.54	0.716	0.717	0.000	0.6	0.857	0.814	0.05	0.662	1
**Middle east**	0.889	0.282	0.716	1.102	1.166	0.229	0.908	1.497	1.175	0.257	0.889	1.552
**Constant**	4.631	0.000	3.616	5.93	2.82	0.000	2.092	3.801	2.966	0.000	2.12	4.151
**N**	11.158											
**Log likelihood**	-13159.9											
**Likelihood Ratio**	chi2(39) = 2468.25											
**Prob > chi2**	0.0000											

Table S4.3: Transfeminine people

Transfeminine people	Mild symptoms				Moderate symptoms				Severe symptoms			
	RRR	p-value	95% Conf. Interval		RRR	p-value	95% Conf. Interval		RRR	p-value	95% Conf. Interval	
**Age group:**												
**Young (18-24)**	0.981	0.887	0.759	1.27	1.121	0.436	0.841	1.496	1.224	0.194	0.902	1.66
**Adult (25-34)**	(base)				(base)				(base)			
**Older adults (35+)**	0.649	0.001	0.501	0.84	0.45	0.000	0.329	0.617	0.456	0.000	0.325	0.639
**Education**												
**No education/primary**	0.945	0.794	0.62	1.443	1.294	0.294	0.8	2.095	1.178	0.531	0.705	1.97
**Secondary education**	0.781	0.031	0.624	0.977	0.961	0.766	0.742	1.245	0.874	0.337	0.664	1.151
**Higher education**	(base)				(base)				(base)			
**Economic vulnerability**												
**Struggling on present income**	1.486	0.004	1.135	1.945	1.962	0.000	1.458	2.641	2.87	0.000	2.097	3.926
**Neither comfortable on present income**	(base)				(base)				(base)			
**Living comfortably on present income**	0.602	0.000	0.47	0.772	0.496	0.000	0.363	0.677	0.526	0.000	0.371	0.745
**HIV status**												
**HIV-negative**	(base)				(base)				(base)			
**HIV-positive**	0.981	0.92	0.675	1.426	1.356	0.155	0.891	2.064	1.672	0.021	1.081	2.587
**I don't know**	1.211	0.156	0.93	1.578	1.526	0.005	1.132	2.055	1.407	0.033	1.029	1.926
**I don't want to answer**	1.132	0.564	0.742	1.727	1.056	0.831	0.64	1.741	0.951	0.853	0.557	1.623
**Social support network**												
**Social support: family**	0.803	0.000	0.736	0.876	0.74	0.000	0.67	0.816	0.663	0.000	0.597	0.737
**Social support: friends**	0.923	0.113	0.836	1.019	0.764	0.000	0.685	0.851	0.672	0.000	0.6	0.752
**Stigma and discrimination**												
**Homophobic reactions**	2.001	0.000	1.564	2.559	3.743	0.000	2.703	5.185	3.465	0.000	2.442	4.917
**Homophobia at the work place**	1.109	0.448	0.849	1.448	1.125	0.437	0.836	1.515	1.183	0.289	0.867	1.615
**Avoiding healthcare**	0.886	0.475	0.635	1.235	1.025	0.893	0.71	1.48	1.376	0.096	0.945	2.005
**Continent dummies**												
**Asia**	0.782	0.09	0.589	1.039	0.618	0.005	0.443	0.862	0.78	0.166	0.548	1.109
**Eastern Europe**	0.992	0.968	0.681	1.446	1.217	0.355	0.803	1.846	1.177	0.475	0.752	1.843
**Sub-Saharan Africa**	1.057	0.759	0.743	1.504	0.651	0.035	0.437	0.97	0.331	0.000	0.209	0.522

**Latin America**	0.696	0.079	0.465	1.042	0.395	0.000	0.239	0.653	0.759	0.267	0.466	1.236
**Middle east**	1.054	0.837	0.637	1.744	1.191	0.529	0.691	2.05	1.864	0.025	1.08	3.218
**Constant**	4.979	0.000	2.951	8.4	3.601	0.000	1.981	6.544	4.763	0.000	2.552	8.892
**N**	3.063											
**Log likelihood**	-3689.75											
**Likelihood Ratio**	chi2(39) = 793.71											
**Prob > chi2**	0.0000											

Table S4.4: Queer/questioning cisgender men and intersex

Queer/questioning cisgender men and intersex	Mild symptoms				Moderate symptoms				Severe symptoms			
	RRR	p-value	95% Conf. Interval		RRR	p-value	95% Conf. Interval		RRR	p-value	95% Conf. Interval	
**Age group:**												
**Young (18-24)**	1.224	0.002	1.078	1.39	1.439	0.000	1.243	1.667	1.407	0.000	1.198	1.652
**Adult (25-34)**	(base)				(base)				(base)			
**Older adults (35+)**	0.803	0.001	0.707	0.911	0.654	0.000	0.558	0.767	0.574	0.000	0.479	0.688
**Education**												
**No education/primary**	0.962	0.748	0.757	1.221	0.961	0.784	0.724	1.276	1.062	0.695	0.787	1.433
**Secondary education**	0.94	0.302	0.837	1.057	1.009	0.894	0.88	1.158	1.028	0.723	0.884	1.195
**Higher education**	(base)				(base)				(base)			
**Economic vulnerability**												
**Struggling on present income**	1.315	0.000	1.141	1.514	1.729	0.000	1.476	2.026	2.421	0.000	2.043	2.868
**Neither comfortable on present income**	(base)				(base)				(base)			
**Living comfortably on present income**	0.624	0.000	0.553	0.704	0.529	0.000	0.454	0.616	0.449	0.000	0.373	0.541
**HIV status**												
**HIV-negative**	(base)				(base)				(base)			
**HIV-positive**	1.175	0.073	0.985	1.402	1.176	0.132	0.952	1.453	1.228	0.083	0.973	1.548
**I don't know**	1.177	0.012	1.037	1.336	1.254	0.003	1.08	1.455	1.308	0.001	1.11	1.541
**I don't want to answer**	1.023	0.823	0.837	1.251	1.004	0.977	0.781	1.29	1.165	0.277	0.885	1.535
**Social support network**												
**Social support: family**	0.843	0.000	0.805	0.884	0.761	0.000	0.721	0.803	0.719	0.000	0.678	0.762
**Social support: friends**	0.833	0.000	0.791	0.877	0.724	0.000	0.683	0.768	0.623	0.000	0.586	0.663
**Stigma and discrimination**												
**Homophobic reactions**	1.734	0.000	1.548	1.942	2.333	0.000	2.015	2.7	2.715	0.000	2.281	3.23
**Homophobia at the work place**	1.229	0.01	1.051	1.437	1.669	0.000	1.406	1.982	1.913	0.000	1.596	2.293
**Avoiding healthcare**	0.929	0.461	0.763	1.131	0.908	0.397	0.727	1.135	1.221	0.087	0.972	1.534
**Continent dummies**												
**Asia**	0.615	0.000	0.526	0.719	0.588	0.000	0.488	0.709	0.482	0.000	0.391	0.594
**Eastern Europe**	1.008	0.935	0.839	1.21	1.035	0.75	0.836	1.281	1.068	0.578	0.847	1.346
**Sub-Saharan Africa**	1.108	0.418	0.865	1.418	0.825	0.191	0.619	1.1	0.457	0.000	0.327	0.637

**Latin America**	0.649	0.000	0.542	0.776	0.67	0.000	0.543	0.827	0.744	0.011	0.593	0.933
**Middle east**	0.939	0.626	0.73	1.209	1.158	0.305	0.875	1.532	1.433	0.014	1.074	1.912
**Constant**	5.429	0.000	4.056	7.266	4.546	0.000	3.255	6.348	5.144	0.000	3.585	7.38
**N**	10.295											
**Log likelihood**	-12462.9											
**Likelihood Ratio**	chi2(39) = 793.71											
**Prob > chi2**	0.0000											
